# TRFill: synergistic use of HiFi and Hi-C sequencing enables accurate assembly of tandem repeats for population-level analysis

**DOI:** 10.1186/s13059-025-03685-5

**Published:** 2025-07-28

**Authors:** Huaming Wen, Jinbao Yang, Xianjia Zhao, Xingbin Wang, Jiawei Lei, Yanchun Li, Wenjie Du, Dongxi Li, Yun Xu, Stefano Lonardi, Weihua Pan

**Affiliations:** 1 School of Computer Science and Technology, University of Science and Technology of China, Hefei, 230027 China; 2 State Key Laboratory of Genome and Multi-Omics Technologies, Shenzhen Branch, Guangdong Laboratory for Lingnan Modern Agriculture, Genome Analysis Laboratory of the Ministry of Agriculture and Rural Affairs, Agricultural Genomics Institute at Shenzhen, Chinese Academy of Agricultural Sciences, Shenzhen, 518120 China; 3 Department of Computer Science and Engineering, University of California, Riverside, CA 92521 USA; 4 College of Computer Science and Technology, Taiyuan University of Technology, Taiyuan, 030024 China

**Keywords:** Genome assembly, Gap filling, Reference-guided genome assembly, Tandem repeats, Segmental duplications

## Abstract

The highly repetitive content of eukaryotic genomes, including long tandem repeats, segmental duplications, and centromeres, makes haplotype-resolved genome assembly hard. Repeat sequences introduce gaps or mis-joins in the assemblies. We introduce TRFill, a novel algorithm that can close the gaps in a draft chromosome-level assembly using exclusively PacBio HiFi and Hi-C data. Experimental results on human centromeres and tomato subtelomeres show that TRFill can improve the completeness and correctness of about two-thirds of the tandem repeats. We also show that the improved completeness of subtelomeric tandem repeats in the tomato pangenome enables a population-level analysis of these complex repeats.

**Supplementary Information:**

The online version contains supplementary material available at 10.1186/s13059-025-03685-5.

## Background

The genome sequence of the vast majority of the eukaryotic species stored in GenBank, EMBL, and other genomic repositories is not complete, due to the difficulties in assembling complex repetitive regions such as segmental duplications, tandem, and interspersed repeats. Repetitive regions in eukaryotic genomes often contain important functional or regulatory elements [1]. For instance, some studies have shown that differences in centromere length between parents can be a contributing factor to chromosomal triploidy in offspring, such as in Down syndrome [2, 3]. Other have demonstrated that loss of heterozygosity (LOH) mutations in human leukocyte antigens (HLA) genes, which are located within a complex repetitive region on chromosome 6, occur in approximately 40% of non-small cell lung cancers and are strongly associated with tumor immune evasion [4]. It is also well established that centromeres and other complex repetitive regions play critical roles in maintaining chromosome structure and stability [5].

The lack of a telomere-to-telomere (T2T) assembly for some of these species has hampered functional, structural, and evolutionary studies of repetitive sequences [6, 7]. Moreover, assembly gaps have been shown to introduce inaccuracies in downstream analyses, such as data contamination [8] and false-positive variant calls [9]. Thanks to recent advances in long-read sequencing technologies, especially the development of PacBio HiFi and Oxford Nanopore ultra-long reads (ONT UL), the completeness of the eukaryotic reference genomes has improved. Several new assembly algorithms have been developed [10–13], which has led to production of T2T or near-T2T genomes for many species [14–17].

Although the sequencing technology and software tools for generating a complete T2T genome assembly are available, there are several limiting factors preventing life scientists from achieving a T2T error-free assembly for their species of interest. Off-the-shelf assemblers for HiFi data like hifiasm [10], hiflye [11], hicanu [12], and verkko [13] can typically assemble only 60–80% of eukaryotic genomes depending on the repeat content. Increasing the genome contiguity of the assembly often requires genome-wide maps (optical, genetic, or physical maps) or proximity-ligation data (Hi-C, linked reads) and time-consuming manual curation. Increasing the genome completeness (i.e., filling the gaps) also involves collecting additional sequencing data and manual intervention to prevent the gap-filling process to introduce assembly mis-joins. In general, gap filling is well studied (see, e.g., Figbird [18], RFfiller [19], DENTIST [20]) but still considered a challenging problem.

With the exception of a few model organisms (e.g., human, mouse, yeast), it is unrealistic to expect life scientists to invest large amounts of time to manually curate assemblies to obtain a T2T gap-free error-free genome (or pangenome) for their species of interest. The cost of finishing these assemblies can be prohibitive (see Additional file 1 for a detailed case analysis). T2T assemblies often require ONT UL which are expensive to generate. Without ONT UL, long repetitive regions cannot be spanned, leading to assemblies with gaps [16, 21, 22]. In many pangenome sequencing projects, only Illumina Hi-C data and either PacBio HiFi, PacBio CLR, or standard Oxford Nanopore data are usually available [23–25]. With the introduction of the PacBio Revio sequencing instrument on the market, the price of PacBio HiFi has reduced much faster than ONT UL and other types of long-read sequencing. We expect that the combination of PacBio HiFi and Illumina Hi-C will be the most popular choice for building large pangenomes in the near future. However, popular assembly pipelines for HiFi + Hi-C data (e.g., hifiasm + 3D-DNA) are still unable to resolve repetitive regions without manual curation. The inability to obtain T2T assemblies for many important species has led to a limited number of population-level studies of repetitive regions [26]. An automatic algorithm is needed to address the problem of filling the gaps in chromosome-level assemblies using only HiFi and Hi-C data. Here we report on a method called TRFill that can locally resolve complex tandem repeats in either haploid or diploid assemblies by taking advantage of (1) HiFi reads, (2) Hi-C reads, and (3) a chromosome-level or T2T genome for a different individual of the same species. TRFill includes a series of algorithmic innovations for the accurate recall of reads that belong to the repetitive region, generating contigs from unitigs, and determining contig positions and correct phasing. The performance of TRFill was tested on the alpha satellite repeats in human centromeres and the tandem repeats in tomato subtelomeres.

## Results

### Overview of TRFill algorithm

**Fig. 1:**
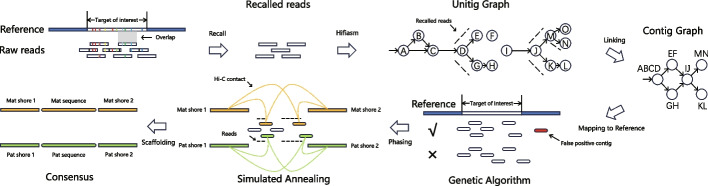
An illustration of the reference-based assembly method proposed here; raw reads are mapped to the reference genome; reads that belong to the region of interest are selected; a unitig graph is built on the recalled reads using hifiasm; the unitig graph is simplified into a contig graph; contigs are ordered using a genetic algorithm; phasing is carried out using Hi-C via a simulated annealing algorithm; an improved assembly is generated

As said above, TRFill uses the reference assembly to identify the reads that belong to the repetitive region and it assembles them locally. We assume that all the reads that belong to the repetitive region are highly similar to each other, but sufficiently different from other repetitive regions. Here we will show that this assumption holds for at least two types of tandem repeats, namely alpha satellite arrays in human centromeres and tomato subtelomeric tandem repeats. The pipeline of TRFill algorithm is shown in Fig. 1 and Additional file 2: Fig. S1.

In the first step, TRFill aligns all the reads to the reference genome and determines the set of reads that belong to the regions of interest. To recall as many reads as possible, TRFill allows reads to only partially align with the genome. To remove reads that originate from other repetitive regions, TRFill performs a hypothesis test by comparing the *k*-mer distributions of the reads to the *k*-mer distribution of the reference.

In the second step, TRFill produces a unitig graph from the set of recall reads using hifiasm (see examples of the unitig graphs in Additional file 2: Fig. S2). TRFill generates a contig-level assembly graph from the unitig graph produced by hifiasm (Additional file 2: Fig. S3 shows two examples in which hifiasm’s contigs are incomplete). Then, TRFill uses a traversal of the unitig graph to pair incoming edges with outgoing edges in each node; based on the pairing, it removes spurious edges on each unitig according to the numbers of supporting HiFi reads. If there is an edge connecting two nodes, as well as a longer path connecting the same two nodes (both supported by enough reads), TRFill removes the single edge and generates the contig from the longer path.

In the third step, TRFill removes spurious contigs assembled from reads that were wrongly recalled and establishes the order of the remaining contigs. These two tasks are challenging because of fragmented alignments between contigs and the reference, and the possible presence of alternative alignments of the same contigs to the reference (due to the repetitive nature of these sequences). TRFill employs several techniques to solve these problems. First, an alignment score for each possible alignment position of the contigs against the reference is calculated via dynamic programming. TRFill creates chains of aligned fragments on the contig and the corresponding aligned fragments on the reference to produce the longest aligned subsequence between the contig and the reference. The length of the longest aligned subsequence is the *alignment score*. All the alignment positions with the highest alignment scores are selected as initial candidates, and then a genetic algorithm is used to select the optimal alignment positions from the pool of candidates. The objective of the genetic algorithm is to cover the repetitive region on the reference exactly once (in case of a haploid assembly) or twice (for a diploid). In each step of the genetic algorithm, the coverage is progressively improved by either removing a contig, or adding a contig, or by switching the alignment position.

After determining the positions of contigs with respect to the reference genome, TRFill carries out the phasing step, in which it assigns contigs to one of the two haplotypes (for diploid genomes). In this last step, TRFill uses a simulated annealing algorithm that (1) maximizes the number of HiFi reads linking contigs from the same haplotype, (2) maximizes the number of Hi-C read pairs linking contigs (and the genomic regions flanking the repetitive region) from the same haplotype, and (3) minimizes the length difference between the reconstructed sequences between the two haplotypes.

### Experiments on human alpha satellite arrays

In our first test, we measured TRFill’s performance on reconstructing the alpha satellite sequences in human centromeres. The alpha satellite sequences constitute approximately 3% of human genome and are composed of tandem repeats with repeat units (monomers) of ~ 171 bp; multiple copies of these monomers can form a higher-order repeat unit of ~ 2 kbp [27]. In many genomes, the alpha satellites are one of the longest uninterrupted tandem repeats.

We decided to assemble the alpha satellites of the diploid human genome HG002 using the human haploid assembly CHM13 as the reference. The assembly of HG002 generated by the Human Pangenome Reference Consortium still contains many gaps [28]. Despite the fact that HG002 is not telomere-to-telomere, we believe it is one of the most accurate assemblies for a human diploid genome. We therefore used it as our “ground truth.”

To establish a baseline, we first assembled the HiFi reads (36 × coverage) and Hi-C reads (69 × coverage) for HG002 into a haplotype-resolved chromosome-level assembly following a commonly used de novo assembly pipeline, namely (i) contig assembly using hifiasm, (ii) scaffolding using 3D-DNA, and (iii) manual inspection of the Hi-C contact map to resolve possible mis-joins. We focused our attention on the assembly of the centromeres. We excluded acrocentric chromosomes (Chr13, Chr14, Chr15, Chr21, Chr22) because in these chromosomes the rDNA sequences are next to the centromeres, which makes the task of identifying the starting or ending positions of the alpha satellite regions nearly impossible. Experimental results in Additional file 3: Table S1 shows that the hifiasm + 3D-DNA (called original thereafter) pipeline was capable of assembling the alpha satellite regions for three centromeres (out of 36 non-acrocentric centromeres) with a completeness of 95% or higher (see Methods for details on how to measure completeness and correctness). Among the 33 centromeres with incomplete alpha satellite sequences, there were 19 with completeness lower than 50% and ten with completeness lower than 20%. In addition, the correctness of alpha satellite sequences was also poor: there were only 17 centromeres with a correctness higher than 90%, and there were four centromeres with a correctness lower than 20%.

**Fig. 2:**
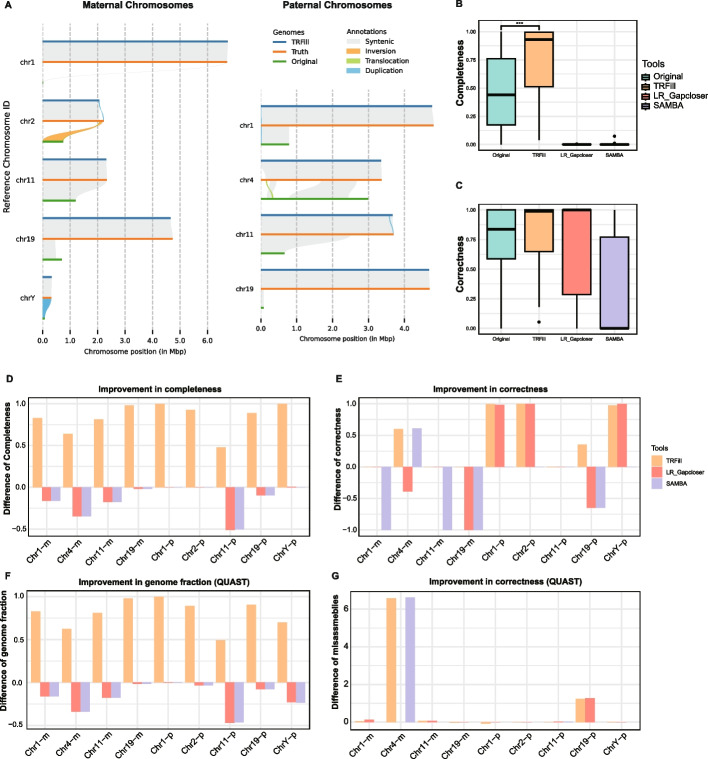
Validation on human centromeric alpha satellite sequences. **A** SyRI synteny plots for the alpha satellite sequences between the hifiasm + 3D-DNA assemblies (original), the TRFill assemblies, and the HG002 assemblies (truth) for nine human chromosomes (left: maternal, right: paternal). **B** Comparing the completeness of the original assemblies, TRFill, LR_Gapcloser, and SAMBA for the alpha satellite sequence of 36 human centromeres; the boxplots show the minimum, maximum, and median values. **C** Comparing the correctness of the original assemblies, TRFill, LR_Gapcloser, and SAMBA for the alpha satellite sequence of 36 human centromeres. **D** Comparing the completeness improvement of TRFill, LR_Gapcloser, and SAMBA with respect to the original assemblies on nine human centromeres. **E** Comparing the correctness improvement of TRFill, LR_Gapcloser, and SAMBA with respect to the original assemblies on nine human centromeres. **F** Comparing the genome fraction improvement of TRFill, LR_Gapcloser, and SAMBA with respect to the original assemblies on nine human centromeres (based on QUAST). **G** Comparing the misassembly density of TRFill, LR_Gapcloser, and SAMBA with respect to the original assemblies on nine human centromeres; the misassembly density is calculated as the number of misassembly reported by QUAST divided by the QUAST genome fraction

We then used TRFill to assemble the alpha satellite sequences for each centromere using the CHM13 T2T assembly as the reference. The position of each alpha satellite region on the HG002 assembly was obtained by lifting the coordinates of the alpha satellite sequences from the CHM13 assembly into the HG002 assembly. Experimental results in Additional file 3: Table S1 shows that TRFill improved the assemblies of the alpha satellite sequences for almost two-thirds of the centromeres in terms of completeness and correctness (namely, 11 out of 18 for maternal and 12 out of 18 for paternal). The completeness of the 23 centromeres improved by 55% on average and in 15 of them TRFill achieved a completeness higher than 95% (Additional file 2: Figs. S4–S8). At the same time, TRFill either improved or maintained the completeness of all the alpha satellites. The overall completeness and correctness of the 36 tested centromeres were significantly improved by TRFill when considering their distributions (Fig. 2B, C). To evaluate the computational requirements of TRFill, we recorded CPU time, wall time, RAM usage, and storage consumption for the analysis of chromosome 1 (see Additional file 3: Table S2).

An apparent weakness of our approach is that we relied on HG002 to identify which centromeres have improved. If we did not have the ground truth, it seems it would be difficult to decide when to pick TRFill assembly over the hifiasm + 3D-DNA assembly. In Methods, we show that one can use the R_AQI and S_AQI scores by CRAQ obtained solely by mapping the original reads to the assemblies (no reference genome is necessary), as well as the HiFi read coverage to classify improved centromeres. We devised two criteria based on these scores, namely *strictly-improved* and *loosely-improved*. The strictly-improved criteria identified nine centromeres that potentially improved (5 maternal and 4 paternal) while the loosely-improved condition identified 25 centromeres (11 maternal and 14 paternal) that potentially improved. Indeed, Fig. 2D, E, F, and G show that TRFill improved the completeness and correctness for all those centromeres labeled “strictly-improved.” Additional file 2: Fig. S9 and Additional file 2: Fig. S10 show that TRFill improved the assemblies for the majority of centromeres labeled as “loosely-improved.” In Fig. 2A, we generated synteny plots between the TRFill assemblies, the ground truth (HG002), and the original assemblies (CHM13) for the nine centromeres labeled as strictly-improved. Observe that although the TRFill assemblies contain imperfections such as collapsed sequence in Chr 20, false-positive sequences in Chr3, and missing sequence missing in Chr 4, they are distinctly better than the original CHM13 assemblies. For instance, on Chr 1 and Chr 11, the alpha satellite sequences assembled on two different haplotypes have significantly different lengths, but they both match the “ground truth” perfectly. Additional file 2: Fig. S11 shows the synteny analysis for the 25 centromeres labeled as loosely-improved. Similar observations were made for the centromeres assembled under this condition.

We also compared TRFill with two state-of-the-art long-read-based gap-filling tools, namely LR_Gapcloser and SAMBA [29]. The results in Fig. 2B–G and Additional file 2: Figs. S5–S10 show that the assembly completeness for LR_Gapcloser and SAMBA is close to zero, and much lower than the original hifiasm + 3D-DNA assemblies. Although the accuracy is high for some of the alpha satellite sequences, it is not meaningful considering the extremely low completeness. TRFill clearly outperforms both tools on these inputs. We believe that LR_Gapcloser and SAMBA are not effective for filling gaps induced by long tandem repeats because they carry out the gap filling from two shores. This progressive extension process can easily fail on tandem repeat because of the high similarity between the repeat units.

To further evaluate the impact of sequencing depth on the performance of TRFill, we subsampled the HiFi and Hi-C reads to various depths and reassembled the centromeric satellite sequences on chromosome 19. The results in Additional file 3: Table S3 show that when the HiFi read depth is no less than 36 × and the Hi-C depth is no less than 17 ×, the accuracy and completeness of the assembly produced by TRFill remain high and comparable to assemblies obtained using the full dataset. However, when the HiFi read depth drops below 18 ×, the assembly quality drops significantly.

### Experiments on tomato subtelomeric repeats

In the second set of experiments, we tested TRFill on the subtelomeric tandem repeat *SolSTE181* in the tomato genome, whose repeat unit is about 181 bp long. For the homozygous tomato Heinz1706 (*Solanum lycopersicum*), we had 38 × coverage of HiFi reads and 48 × coverage of ONT UL reads (N50 of 94,580 bp); for the tomato TS2 (*Solanum lycopersicum*), we had 40 × coverage of HiFi reads and 37 × of ONT UL reads (N50 of 52,704 bp); for the tomato TS281 (*Solanum lycopersicum *var.* cerasiforme*), we had 44 × coverage of HiFi reads and 40 × of ONT UL reads (N50 of 53,839 bp). We also had about 130 × of Hi-C Illumina reads for the three genomes. The Hi-C data for Heinz1706 and the HiFi data for the three genomes was obtained from NCBI SRA BioProject PRJNA733299 and PRJNA756391 [30]. All the other data was generated as part of this study.

First, we assembled the three genomes using hifiasm on the HiFi and ONT UL reads, followed by a scaffolding step using 3D-DNA on Hi-C reads, and manual curation based on the Hi-C contact maps. We used the assembly of Heinz1706 as the reference, while the assemblies of TS2 and TS281 were used as the “ground truth.” Additional file 3: Table S4 shows the tandem repeats of 19 subtelomeres that had been successfully assembled in Heinz1706.

**Fig. 3:**
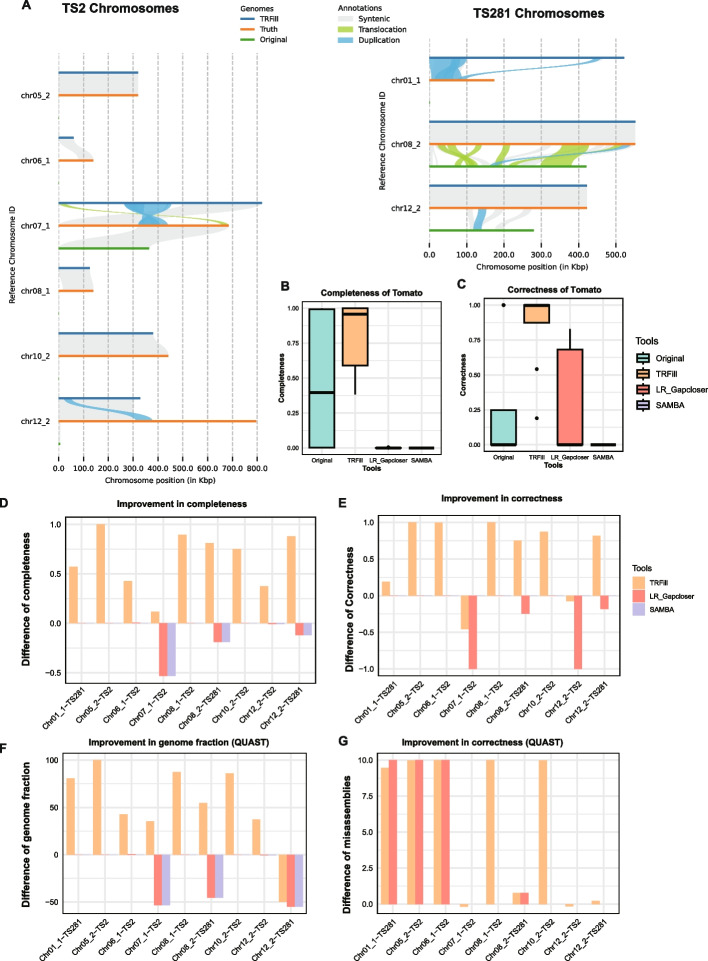
Assembly results on nine subtelomeric tandem repeats in a synthetic tomato diploid genome (using TS2 and TS281 as two haplotypes). **A** Synteny plots between the original assemblies, the TRFill assemblies, and the “ground truth.” **B** Distribution of completeness and correctness of the original assemblies, TRFill assemblies, LR_Gapcloser assemblies, and SAMBA assemblies; boxplots show the minimum, maximum, and median values in the distributions. **C** Comparing the completeness improvement of TRFill, LR_Gapcloser, and SAMBA with respect to the original assemblies. **D** Comparing the correctness improvement of TRFill, LR_Gapcloser, and SAMBA with respect to the original assemblies. **E** Comparing the genome fraction improvement of TRFill, LR_Gapcloser, and SAMBA with respect to the original assemblies (based on QUAST). **F** Comparing the misassembly density of TRFill, LR_Gapcloser, and SAMBA with respect to the original assemblies; the misassembly density is calculated as the number of misassembly reported by QUAST divided by the QUAST genome fraction

Second, we compared these assemblies with the assemblies of TS2 and TS281 published in Zhou et al. [30]. These two reference assemblies were produced using hifiasm for contig generation and RagTag [33] for scaffolding. We compared the subtelomeric tandem repeats in these two assemblies with their respective ground truth and with the high-quality Heinz1706 reference genome. We determined that 12 of the 19 subtelomeres in TS2 had tandem repeats with low completeness (< 98%) or low correctness (< 98%); in TS281 only three of the 19 subtelomeres in TS281 had tandem repeats with low completeness (< 98%) or low correctness (< 98%) (Additional file 3: Table S5A). We decided to use the reference genome as a guide for the reassembly of the tandem repeats in these 12 + 3 subtelomeres using TRFill. As shown in Fig. 3A, C, and Additional file 3: Table S6A, TRFill improved the quality of 11 of the 15 subtelomeres in either completeness and correctness (while the other criteria did not decrease) or both. On the 11 subtelomeres, the completeness and correctness improved by 27% and 12% on average, respectively (Fig. 3B, C, Additional file 3: Table S6A). As we did for human centromeres, we determined that 6 subtelomeres (4 on TS2 and 2 on TS281) were labeled strictly-improved and 19 subtelomeres (11 on TS2 and 8 on TS281) were labeled loosely-improved, out of the 38 subtelomeres tested. TRFill indeed improved the completeness and QUAST [31, 32] genome fraction for the 6 subtelomeres labeled strictly-improved, achieving a higher correctness in most of them (Additional file 2: Fig. S12). For the 19 subtelomeres labeled loosely-improved, TRFill produced assemblies with higher or comparable completeness and similar correctness compared to the original assemblies (Additional file 2: Fig. S13). CPU time, wall time, RAM usage, and storage consumption for processing chromosome 1 for both TS281 and TS2 are presented in Additional file 3: Table S2.

Third, we measured the ability of TRFill to carry out phasing. We started by creating a synthetic diploid tomato genome where TS2 was used as the maternal haplotype and TS281 was used as the paternal haplotype. The HiFi and Hi-C data for the synthetic diploid genome was generated by merging the data sets for TS2 and TS281. Additional Hi-C reads between the maternal and paternal chromosomes were added to simulate inter-chromosomal interactions (details in Methods). These data sets were assembled into a haplotype-resolved chromosome-level assembly using hifiasm for contig assembly/phasing, 3D-DNA-based scaffolding, and Hi-C map-based manual curation. In this assembly, there were in total 28 subtelomeres (17 on TS2 and 11 on TS281) with comparatively low-quality tandem repeats (completeness < 98% or correctness < 98%) while the corresponding subtelomeres in the reference contained high-quality tandem repeats (Additional file 3: Table S5B). After reassembly with TRFill, the tandem repeats in 15 of these 28 subtelomeres were improved in either completeness or correctness, and neither of these metrics ever decreased. On the 15 subtelomeres, the average completeness and correctness were improved for 53% and 57%, respectively (Fig. 3B, Additional file 2: Figs. S14–S17, Additional file 3: Table S6B). We also used the “strict” and “loose” criteria defined in the previous section to determine which subtelomere were potentially improved without using the ground truth. Based on these criteria, nine subtelomeres were labeled strictly-improved (6 on TS2 and 3 on TS281) and 28 were labeled loosely-improved (13 on TS2 and 15 on TS281). Observe that TRFill indeed improved the completeness and correctness for almost all the strictly-improved subtelomeres (Fig. 3C–F). For the 28 loosely-improved subtelomeres, TRFill achieved higher (or comparable) completeness and correctness for most of them (Additional file 2: Fig. S18).

In Fig. 3A, synteny plots were produced between the ground truth and the subtelomeric regions before and after TRFill reassembly (see also Additional file 2: Figs. S19–S22). Observe that the TRFill reassemblies have stronger synteny with the ground truth than the original assemblies, despite a few structural variations. Notably, the tandem repeats in some subtelomeres (e.g., chr05_2 and chr08_1) were missing from the synteny plots before reassembly, but TRFill was able to reassemble them with high completeness and correctness.

Population-level analysis of *SolSTE181* sequences exhibits the local law of sequence similarity of tandem repeats.

After the extensive validation of our method on synthetic data reported above, TRFill was used to assemble de novo the subtelomeric tandem repeats of 29 tomato genomes (see Additional file 3: Table S7 for a list of the accessions). We used as a reference the 19 subtelomeric tandem repeats in the Heinz1706 assembly. Out of the 29*19 = 551 subtelomeres, TRFill improved the length of tandem repeats in 494 (90%) of the cases. The cumulative length of all tandem repeats increased from ~ 167 to ~ 252 kbp. We updated 494 subtelomeric repeats with the TRFill assemblies and kept the original sequences for the remaining 48 (Additional file 3: Table S8). TRFill assemblies were much longer than the original assembly and in more than half of the cases they contained within an exact copy of the original assemblies. When we compared the TRFill assemblies to the contigs in Zhou et al. (which were not included in the chromosome-level assemblies), we found that more than 32 Mbp aligned to those contigs. This indicates that TRFill was able to reconstruct sequences missing from the original assemblies.

**Fig. 4:**
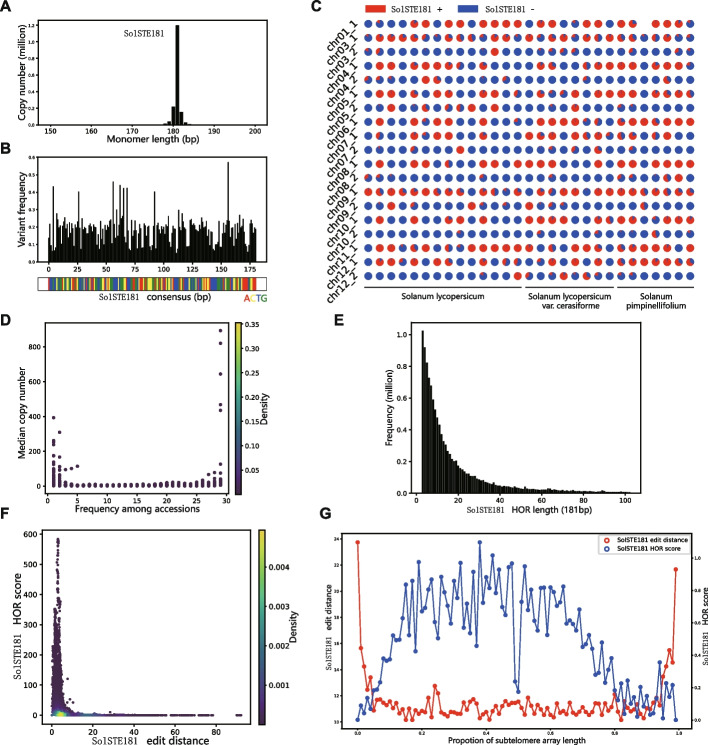
General population-level analysis on *SolSTE181* monomers and HORs. **A** Frequency histogram representing the distribution of *SolSTE181* monomers.** B** Variant frequency of each of the 181 nucleotides in *SolSTE181* consensus monomer, where the variant frequency is defined as (1.0 − the frequency of most frequent base). The DNA content of the *SolSTE181* consensus is shown below the graph (red: A; yellow: C; blue: T; green: G). **C** Pie charts showing the proportions of *SolSTE181* monomers on the forward (red, +) and reverse (blue, −) strands respectively in 19 subtelomeres (rows) by 29 genomes (columns). **D** Median SolSTE181 copy number plotted against the number of accessions in which they were found. **E** Length distribution of HORs (where the length is the number of 181 bp monomers contained in the HOR). **F** Density plot of *SolSTE181* HOR scores versus edit distances from the *SolSTE181* consensus, across all accessions. **G** Plot of *SolSTE181* HOR scores (blue) and edit distances (red) along all subtelomeric regions (scaled 0.0–1.0 accordingly)

Given the improved subtelomeric repeats, we performed a population-level analysis on the *SolSTE181* repeat units along the lines of the analysis on the alpha satellite array *AthCEN178* in *Arabidopsis thaliana* [26]. First, we determined that the monomer is a repeat unit of about 181 bp. Exactly 1,677,830 monomers were identified in total (Fig. 4A). We observed that (i) a significant proportion of the monomers (149,012) were unique to a single accession, (ii) the monomers that were unique to a single accession had high copy numbers (a similar observation was reported in the *AthCEN178* study) (Fig. 4D), and (iii) the monomers shared by almost all accessions also had high copy numbers (which was not reported for *AthCEN178*). A consensus monomer was built from all monomers and the average variant frequency of each base was obtained (Fig. 4B). While the *AthCEN178* monomer had a higher variant frequency in the first half of the monomer than the second half, the variant frequency in the *SolSTE181* monomer was relatively uniform. In general, the average variant frequency of *SolSTE181* monomer was significantly higher than that of *AthCEN178*, indicating a lower stability of subtelomeres compared to centromeres. In addition, strong strand biases were observed among *SolSTE181* monomers (Fig. 4C).

We also carried out an analysis on HORs. We defined an HOR as a tandem duplication of at least three consecutive monomers, each with no more than five substitution or single-base insertions/deletions. Based on this definition, a total of ~ 12.1 million HORs were found. The length distribution of these HORs is shown in Fig. 4E. The most frequent HOR, composed of three monomers (~ 543 bp), was observed more than a million times. To quantify the repetitive nature of HORs, we defined the *HOR score* of a monomer as the number of HORs to which the monomer belonged divided by the total number of monomers on its subtelomere. We also recorded the edit distance of each monomer from the consensus monomer of its subtelomere. The HOR scores are illustrated in Fig. 4F, and the edit distances are shown in Fig. 4G. Observe that most monomers had low HOR scores and edit distances. Monomers with high HOR scores and edit distances represented the most repetitive monomers or monomers that have undergone significant mutations. When we examined these two scores across the subtelomeres (Fig. 4G), the central regions of the subtelomeres showed the greatest amount of repetition but appeared to be relatively stable in terms of sequence variations.

**Fig. 5:**
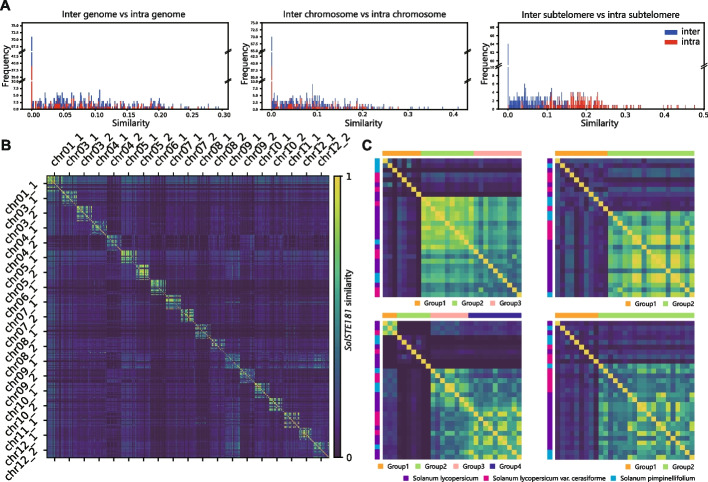
Monomer-based population-level analysis of *SolSTE181* sequence similarity. **A** Sequence-similarity analysis of *SolSTE181* between inter- (blue) and intra- (red) genome, chromosome and subtelomeric regions.** B** Sequence-similarity analysis between all pairs of *SolSTE181* sequences in 551 subtelomeres. **C** Diagonal submatrices of the matrix in **B** corresponding to the sequence similarity between 29 subtelomeres at the same position in all the accessions (the order of accessions was switched so that the accessions in the same group were next to each other column-wise; the horizontal colored bars represent the similarity groups, while the vertical colored bar represents the three taxonomic groups)

Finally, a sequence analysis was performed on the monomers of *SolSTE181*. We compared the sequence similarity (i) between the intra-genome monomers and inter-genome monomers, (ii) between intra-chromosome and inter-chromosome monomers in the same genomes, and (iii) between intra-subtelomere and inter-subtelomere monomers in the same genomes. There were no significant differences in the sequence similarity for (i) and (ii). However, the intra-subtelomere monomers had much higher similarity than the inter-subtelomere monomers (iii) (Fig. 5A). This observation suggested that the similarity between the monomers of *SolSTE181* is not uniformly distributed and strongly depends on the locations of the monomers. In fact, *SolSTE181* monomers can be partitioned into non-overlapping local regions in which the local monomers are significantly more similar. A previous study had similar findings on human centromeres [27]. We called this “*the local law of sequence similarity for tandem repeats*.” To study the sequence similarity of subtelomeres across tomato genomes, in Fig. 5B we calculated the monomer similarity between all pairs of subtelomeric regions in the 29 tomato accessions. We observed that the subtelomeres of closely related genomes exhibited higher sequence similarity than the different subtelomeres (even in the same genome). We also observed that the sequence similarity between subtelomeres is not related to the evolutionary distance between the tomato accessions (Fig. 5C). This indicates that the concept of “local region” should be used for a population of closely related accessions rather than a single genome, and the *local law of sequence similarity for tandem repeats* may also hold at the population-level. Since Additional file 2: Fig. S23 on the centromeric alpha satellite sequences of human CHM13 and HG002 genomes points to similar finding, we speculate that the *local law of sequence similarity for tandem repeats* may be a general law for tandem repeats.

## Discussion

Traditional population-level genome analyses start from aligning the reads obtained from sequencing the population under study to a high-quality reference genome. The position of the mapped reads can be used by a reference-based assembler (e.g., RagTag [33]) to generate assemblies for all the individuals in the population, drastically simplifying the assembly process. However, this method (i) introduces a reference-based bias in the assemblies and (ii) often fails to correctly assemble contigs in regions with high structural variation or high repetitive content. Due to these weaknesses, recent large-scale eukaryotic pangenomes have been built de novo, i.e., without relying on a reference genome. The de novo method has a much higher cost: it requires multiple types of sequencing data, higher depth of coverage, and time-consuming manual curation. There is a need for genome assembly methods which can use lower-coverage long reads and take advantage of the reference genome to generate high-quality assemblies for different individuals or accessions in a population. In this study, we designed an assembler that responds to these needs. Our method uses the reference genome to recall the reads for a de novo reassembly of targeted genomic regions. We showed that TRFill can significantly improve the completeness and correctness of tandem repeats such as human centromeric alpha satellite arrays and tomato subtelomeric tandem repeats (in both haploid and diploid form) using HiFi and Hi-C without any manual intervention.

Our approach assumes that there is sufficient similarity between the genomic regions that need to be reassembled and the corresponding regions on the reference to properly recall the reads. We have no doubts that this assumption holds for non-repetitive regions. For repetitive regions, however, it is not certain that the assumption holds in general. Although our method was successful on human alpha satellite arrays and tomato subtelomeric tandem repeats, additional studies on other species and other types of tandem and interspersed repeats (such as rDNA, telomere, and transposons) are needed to establish the limits of our approach. In addition, it could be worth studying the performance of our method for the assembly of non-repetitive regions with ultra-low-depth long reads.

A critical step in TRFill is the accurate recalling of the reads that belong to the genomic region of interest. TRFill assumes a linear relationship between the number $\mu$ of expected rare *k-*mer in the aligned read region and the number $y$ of observed rare *k*-mer in the aligned reference region, namely $\mu =\delta y$ where parameter delta $\delta$ captures this relationship $(0<\delta \le 1)$. We obtained experimentally $\delta$ from the study of *Arabidopsis thaliana* data. Although the chosen $\delta$ worked well for human centromeres and tomato subtelomeres, it is possible that choosing a specific value of $\delta$ for each genomic region could improve the read recalling process. A method for determining $\delta$ for each region would use the flanking sequence around the region of interest. Similarly, the TRFill parameter that represents the HiFi sequencing error rate could be more accurately estimated for each genome by taking advantage of the correctly assembled sequences in the non-repetitive regions.

Although TRFill significantly improved the assembly quality on human and tomato data, we believe there is room for improvement in some algorithmic and technical details. We are confident that by integrating advanced techniques used in the state-of-the-art long-read assemblers (e.g., hifiasm) in future versions of TRFill, the correctness, completeness, speed, and space efficiency can be significantly improved.

Observe that TRFill uses the reference genome in the process of recalling the reads but also in determining the order/orientation of contigs during scaffolding. Using the reference genome should help improve the scaffolding in regions when few HiFi and Hi-C are present (in regions with a large number of HiFi and Hi-C links, the order and orientation of the contigs is mostly decided by the reads). However, using the reference for scaffolding can introduce errors. Assembly errors could be introduced in those rare cases when the structural variations in the genome to reassemble are longer than the contig size. To address the tradeoff between correctness and contiguity, it could be worth studying an adaptive strategy to adjust the importance of the reference genome according to the number and reliability of HiFi and Hi-C links.

While TRFill improved the completeness and correctness of most tandem repeats in the experiments presented above, there were a small number of examples in which the new assemblies were worse than the original ones. Since we cannot assume that TRFill is always going to improve the assemblies, it is important to have a robust method that can accurately determine when the TRFill reassemblies should be discarded. While the CRAQ-based method we proposed appears to be sufficiently accurate, better evaluation tools that can detect poorly assembled genomic regions are needed.

In this study, we used high-quality reference genome to evaluate the accuracy of the TRFill assemblies. Some of these reference genomes were generated by authoritative consortia, while others were produced as part of this study. All reference genomes were obtained using multi-platform high-depth sequencing approach combined with extensive manual curation, ensuring a high level of assembly quality. Nevertheless, these reference genomes are unlikely to be 100% accurate and complete. Therefore, additional data types may be considered to further validate the quality of the TRFill assemblies. For example, some studies have shown that optical maps can be used to validate the assembly of complex repetitive regions [34].

Although HiFi + Hi-C is the predominant sequencing combination in most recent eukaryotic genome projects, sequencing technologies are rapidly evolving, and alternative approaches will emerge. Additional work will be needed to enable TRFill to support a broader range of data types, including long-read technologies such as ONT Duplex and Simplex, and new chromatin conformation capture technologies such as Dovetail Omni-C, Dovetail Micro-C, PacBio CiFi, and ONT Pore-C.

Overall, the primary application domain for TRFill is in constructing high-quality pangenomes for species that already have a high-quality (T2T or near-T2T) reference genome. For example, with the availability of the haploid human T2T genome CHM13, TRFill can be applied to the large-scale construction of human diploid pangenomes. This will enable the accurate resolution of tandem repeat sequences and complex repetitive regions with relatively low sequencing depth and minimal manual effort. More generally, we believe that TRFill will enhance the completeness and accuracy of genome assemblies for the pangenomes of major eukaryotic species. Furthermore, TRFill will advance studies on the evolution of the repetitive regions, which have become an area of growing interest in recent years [35]. For instance, studies have explored centromere sequence evolution in species such as humans [36], *Arabidopsis thaliana* [26], and cotton [37]. In addition, complex repetitive regions in cancer genomes have been found to harbor important somatic structural variants [38]. We are confident that TRFill and its future revisions will facilitate new scientific discoveries for complex repetitive genomic regions and significantly improve our understanding of the genome architecture of eukaryotes.

## Conclusions

This study introduced a novel strategy for achieving high-quality, population-scale genome assemblies by leveraging a T2T-level reference genome to guide the assembly of other genomes from the same species. Unlike the traditional reference-based approaches that directly align raw sequencing reads to the reference, our method uses the reference solely to retrieve candidate reads from target regions, which are subsequently assembled de novo. This strategy reduces assembly complexity while preserving accuracy in structurally variable regions. Our TRFill algorithm accurately fills assembly gaps using only PacBio HiFi and Hi-C data, without relying on the costly ONT UL reads. Our experiments on human centromeric satellite sequences and subtelomeric regions in tomato showed that TRFill is capable to successfully reconstruct large tandem repeats that are typically unresolved in draft assemblies. Additional experiments showed that TRFill can enhance the sequence completeness of subtelomeric regions in a real tomato pangenome. These improved assemblies enabled a systematic and detailed analysis of the similarity of repetitive elements in the pangenome.

## Methods

We use the term *reference* to indicate the high-quality genome assembly guiding TRFill and the term *query* to refer to the genome to assemble. We use the term *target area of interest* (TOI) to indicate the region that needs to be reassembled in the query genome. We use the term *reference TOI* to denote the corresponding region on the reference genome. The input to TRFill is (i) a reference genome, (ii) a chromosome-level query genome assembled using HiFi and Hi-C reads using a standard pipeline, e.g., hifiasm + 3D-DNA, (iii) HiFi and Hi-C reads for the query genome, and (iv) the starting and ending positions of TOI on the query genome. The output of TRFill is the reassembled sequence of the TOI in the query genome. TRFill operates in four phases: (1) recall all the HiFi reads that belong to the reads for the TOI; (2) assemble these recalled reads into contigs; (3) determine the contig positions and orientations using the reference; and (4) determine the phasing of the contigs. The TRFill pipeline is shown in Fig. 1 and Additional file 2: Fig. S1.

### Read calling

TOIs are identified using the synteny between reference and query, which can be obtained using SyRI [39]. More specifically, the shortest continuous region on the reference that covers the TOI on the query is selected as the reference TOI. All HiFi reads are aligned to the reference TOI via winnomap2 [40] using the “-x map-pb” option. Any HiFi read which is partially aligned to the reference TOI is selected into a candidate set. Due to the expected difference between the reference and the query, it is not necessary to have the entire read aligned.

A hypothesis test is performed to remove as many false-positive reads from the candidate set as possible. The hypothesis is based on the presence of rare *k*-mers (in this study, we use 21-mers appearing less than 4 times in the whole reference genome). To obtain these rare *k*-mers, Jellyfish [41] is run to obtain the frequency of all the *k*-mers on the reference. For each read in the candidate set, we focus on its alignment with TOI on reference, and check if the rare *k*-mers in the aligned region in the reference also occur in the aligned region in the query. More formally, for an alignment $A$ between the aligned region $R$ on reference and the aligned region $Q$ on read, let $Y$ and $X$ ($X\le Y$) be the number of rare *k*-mers in $R$ and the number of rare *k*-mers in both $R$ and $Q$, respectively. We want to check how close are the value ($x$) of $X$ to the value ($y$) of $Y$. We assume $X$ to be normally distributed, i.e., $X\sim N(\mu ,{\sigma }^{2})$, where $\mu$ is the number of rare *k*-mers appearing in both $R$ and the corresponding region in the real query genome, and ${\sigma }^{2}$ is the sequencing error rate. Since the query genome and $\mu$ are unknown, we further assume that $\mu$ is a function of $y$ if $R$ and $Q$ are homologous (i.e., $R$ and $Q$ are from the same tandem repeat in two different individuals, such as the centromeres of the same chromosome in two different genomes). Since $\mu$ is always smaller than or equal to $y$ by definition, we can assume that $\mu =\delta y (0\le \delta <1)$. While parameter $\delta$ is affected by many factors such as the difference in sequence composition between reference and query and the similarity in sequence composition within the TOI for both reference and query, for simplicity, we assume $\delta$ to be constant. Since ${\sigma }^{2}$ is determined by the sequencing technology and only HiFi reads are used in this study, ${\sigma }^{2}$ is also assumed to be constant. With these assumptions in mind, the density function of $X$ can be described as

$${f}_{X}\left(x\right)=\frac{{e}^{-\frac{{(x- \delta y)}^{2}}{2{\sigma }^{2}}}}{\sqrt{2\pi }\sigma }.$$

Both $\delta$ and ${\sigma }^{2}$ can have a drastic effect on the performance of TRFill. In this study, the values of $\delta$ and ${\sigma }^{2}$ were estimated from the centromeric regions of *Arabidopsis thaliana*. A near-T2T-level assembly of the genome of *Arabidopsis thaliana* called ColCEN (accession Col-0) with almost complete centromeric regions was used as the reference. HiFi reads from the centromeric regions of accession Ler-0 [26] of *Arabidopsis thaliana* were aligned to the centromeric sequences of ColCEN. To obtain the HiFi reads from each centromere of Ler-0, we only kept the reads with high-quality alignments against the Ler-0 assembly (which contains almost complete centromeres). We denote by ${A}_{i}$, $1\le i\le N$ the set of alignments between HiFi reads from the centromeric regions of accession Ler-0 and the centromeric regions of ColCEN. Each ${A}_{i}$ has associated variables ${R}_{i}$, ${Q}_{i}$, ${X}_{i}$, ${Y}_{i}$, ${x}_{i}$, and ${y}_{i}$ defined as we did above. The probabilistic density function of ${X}_{i}$ can be described as

$${f}_{{X}_{i}}\left({x}_{i}\right)=\frac{{e}^{-\frac{{({x}_{i}- \delta {y}_{i})}^{2}}{2{\sigma }^{2}}}}{\sqrt{2\pi }\sigma }.$$

By combining the probabilistic density functions of all ${X}_{i}$ ($1\le i\le N$), the log likelihood function is obtained as

$$ln f\left(\delta , {\sigma }^{2}\right)=-\frac{nln \left(2\pi {\sigma }^{2}\right)}{2}-\frac{{\sum }_{i=1}^{N}{\left({x}_{i}- \delta {y}_{i}\right)}^{2}}{2{\sigma }^{2}}.$$

By maximizing the log likelihood function, we can obtain a closed form for $\delta$ and ${\sigma }^{2}$

$$\delta =\frac{{\sum }_{i=1}^{N}{y}_{i}{x}_{i}}{{\sum }_{i=1}^{N}{y}_{i}^{2}}$$

$${\sigma }^{2}=\frac{{\sum }_{i=1}^{n}({x}_{i}- {\delta y}_{i})}{N}.$$

Using these max likelihood estimates of $\delta$ and ${\sigma }^{2}$, a two-sided test can be performed on each alignment $A$ (with associated variables $R$, $Q$, $X$, $Y$, $x$, and $y$ defined above). Let ${Z}_{\frac{\alpha }{2}}$ be the value of a normal distribution at a certain significance level (e.g., $\alpha =0.05$). If $x$ falls within $\left[\delta y-{Z}_{\frac{\alpha }{2}}\sigma , \delta y+{Z}_{\frac{\alpha }{2}}\sigma \right]$, we recall the read. Otherwise, the read is a false positive and should be discarded. When a read has multiple alignments to the reference TOI, we perform a test for all alignments and recall the read as long as one alignment passes the test.

### Contig assembly

After recalling HiFi reads, we assemble them into contigs. A unitig-level assembly graph is built from the reads with hifiasm. The default settings are used for a diploid genome, while option “-l0” is used for a haploid genome. Although hifiasm also provides a contig-level assembly graph, in some cases the contigs assembled directly from hifiasm have low completeness (see Additional file 2: Fig. S3). Instead, TRFill generates contigs from the unitig graph.

The HiFi reads are aligned to the unitig graph using winnowmap2 [35]. The number of supporting reads for each edge of the graph is counted. A modified breadth-first search (BFS) is used to traverse the unitig graph from zero-indegree nodes. A BFS-traversal graph, containing the same vertices as the unitig graph and all the edges in the unitig graph traversed by the modified BFS, is constructed, as follows. At each branching node *v*, rules (1–4) are followed. (1) We allow node *v* to be visited $\lfloor (id+od)/2\rfloor$ times (instead of just once), where $id$ and $od$ are the in-degree and out-degree of $v$, respectively; (2) for a successor node $w$ of $v$, if there exists a longer path from $v$ to $w$ in which $o$ is the direct successor node of $v$, $o$ is chosen next rather than $w$ (i.e., edge $v\to w$ is seen as a transitive edge). Figure 1 shows a simple example with nodes $A,B,C$ in unitig graph. Observe that there is a chance for $w$ to be visited from $v$, next time *v* is being visited. In case (2) does not apply, (3) among all the successors of $v$ with enough supporting reads, the one with the largest number of supporting reads is chosen for visiting for this visit of $v$ (see nodes $M,O,N$ in unitig graph of Fig. 1 for an example). Similarly, there is a chance for other successors to be visited from $v$, next time $v$ is being visited. (4) If no successor of $v$ has enough supporting reads, all successors of $v$ are chosen for this visit of $v$ (see $D,E,G$ in unitig graph of Fig. 1 for an example). The BFS-traversal graph obtained by this modified BFS includes the order of the nodes visited and the corresponding timestamps. A node visited more than once will appear multiple times in the BFS-traversal graph with different order numbers.

A depth first search (DFS) is then used to traverse the BFS-traversal graph to generate contigs. As we did previously, each node is allowed to be visited $\lfloor (id+od)/2\rfloor$ times, where $id$ and $od$ are the in-degree and out-degree. respectively. At each branching node $v$, among all the successors of $v$ such that their maximum number of visits has not been reached, we choose the one with the earliest timestamp in the BFS-traversal. We also mark where the graph needs to be cut after finishing the traversal to generate independent contigs. There is, however, a special situation when the graph can be solved. Additional file 2: Fig. S24A shows an example in which $E\to A\to B\to C\to A\to D$ is a path that can generate a contig, although $A$ is a branching node. To distinguish this case from other unsolvable situations, when a node is revisited and a loop is generated, we remove the cutting flag at that node. In the example, although $A$ is marked to be cut after its first visit, the flag is removed at the second visit and we can generate the contig $E\to A\to B\to C\to A\to D$. It is also possible to have an unsolvable branching node nested within a solvable loop. Additional file 2: Fig. S24B shows an example in which $B$ is an unsolvable node and will be cut after the traversal while the other part of the loop is solvable, thus three contigs, namely $E\to A\to B, C\to A\to D$, and $F$, can be generated.

After the DFS traversal, the traversal graph is cut at the branching nodes determined by the following rules (1–3). Let $v$ be a branching node. (1) If $v$ has one predecessors and multiple successors, the outgoing edges of $v$ are cut and the incoming edge is kept. (2) If $v$ has multiple predecessors and one successor, the incoming edges of $v$ are cut and the outgoing edge is kept. (3) If $v$ has multiple predecessors and multiple successors, all the incoming edges and outgoing edges of $v$ are cut.

### Determining the position and orientation of contigs

After obtaining the contigs, TRFill determines the contig positions and orientations relative to the reference. First, the contigs are mapped to the reference TOI with winnowmap2 and filtered out if they have a poor alignment. A contig is discarded if either (1) its alignment to the reference TOI is shorter than 10% of its lengths, or (2) the total length its alignment to the reference TOI is less than twice the total length its alignments to other regions on reference genome.

TRFill then determines the positions and orientations of the remaining contigs according to their alignments to the reference. This step is challenging due to the fragmented alignments caused by the differences between the reference genome and the query genome and the multiple alignments due to the highly repetitive sequences. We framed this problem in a combinatorial optimization model. Let us assume that a contig is mapped to the reference TOI along a set of fragmented alignments. For each alignment, we define an aligned pair defined by (reference region, contig region) as an *element*. By sorting the elements for a given contig by their starting positions on the contig, we obtain a sorted sequence ${L}_{c}[1\dots N][1\dots N]$ of elements for the contig. On reference side, we check all subregions of the TOI with a length equal to the contig length starting from the element aligned to ${L}_{c}[1]$. For each of these subregions, a sequence ${L}_{r}[1\dots M]$ of elements on the reference side is obtained. The alignment score between the contig and a subregion is the length of the longest matching subsequence (see Definition 1 below) between ${L}_{c}$ and ${L}_{r}$ where the “matching” in LMS represents the “alignment” between elements.

### Definition 1 (longest matching subsequence)

Input: a sequence $A[1\dots N]$ of elements, a sequence $B[1\dots M]$ of elements, and a matching function $f$ in which $f(x, y)=True$ if element $x$ in $A$ matches element $y$ in $B$. Output: a subsequence $A{\prime}$ of $A$ and a subsequence $B{\prime}$ of $B$ such that (i) $|A{\prime}|$ = $|B{\prime}|$ and (ii) for any position $k (1\le k\le |A{\prime}|)$, $f(x, y)=True$ for element $x$ in $A{\prime}$ element $y$ in $B{\prime}$ at the position *k*th position.

Since LMS is a variant of the longest common subsequence problem, LMS can also be solved optimally in $O(NM)$ time. This complexity, however, might be too high for some contig with very fragmented alignments. To speed up this step, we generate an approximate solution of LMS by solving the longest increasing subsequence problem (LIS) [42]. Given the sequence ${L}_{r}$ of elements on the reference, a corresponding coordinate sequence $L[1\dots M]$ of same length is built according to the sequence ${L}_{c}$ of elements on the contig. For any $j (1\le j\le M)$, $L[j]=i$ if ${L}_{r}[j]$ is aligned to ${L}_{c}[i]$. A longest increasing subsequence $LL[1\dots P]$ satisfying criteria 1 and 2 below is obtained with a binary search algorithm [43] in $O(N+M log M)$ time. The criteria are the following. (1) $LL$ is a subsequence of $L$; (2) for any choice of $p, q (1\le p<q\le P), LL[p]\le LL[q]$; (3) there exists no other subsequence of $L$ satisfying (1) and (2) with length longer than the length of $LL$. The length of the longest increasing subsequence is used as the alignment score between the contig and the subregion of the reference TOI. For each contig, we select as candidates at most seven subregions with the highest alignment score.

TRFill uses a genetic algorithm to select the optimal subset of these contigs and determine their positions. Each contig, each alignment position, and each possible combination of candidate alignment positions of contigs represent a *locus*, an *allele*, and an *individual*, respectively, in the terminology of genetic algorithms. We simulate the evolution process by carrying out *switching* operations (i.e., switch alignment position of one contig between two individuals), *variation* operations (i.e., delete or insert one contig from or to individual), and *natural selection* operations (select individuals with a probability proportional to the fitness score). The objective is to obtain individuals that maximize the objective function (also called the *adaptivity* of individuals). The objective function has two components, one related to the coverage and one related to the distance. The coverage component aims to ensure that the contigs collectively cover the entirety of the reference TOI exactly $T$ times, where $T$ represents the ploidy of the query genome (e.g., $T=2$ for a diploid genome). The distance component aims to maximize the distance between contigs to reduce the extent of overlap between them. Observe that these two objectives work against each other: covering the reference more extensively tends to bring contigs closer together.

Formally, let ${l}_{t}$ be the total length of the subregions of the reference TOI with a coverage of $t$, and ${d}_{ij} (i,j\in \left[1, C\right])$ be the distance between alignment midpoints of contig $i$ and contig $j$ on the reference TOI where $C$ represents the total number of contigs. The objective functions for haploid and diploid genomes are as follows.

$${f}_{haploid}=2\left({l}_{1}-{l}_{0}-{l}_{>2}\right)+{l}_{2}-2.5{l}_{<0}+\frac{1}{2}{\sum }_{i=1}^{C}{\sum }_{j=i+1}^{C}{d}_{ij}$$

$${f}_{diploid}=2\left({l}_{\ge 2}-{l}_{0}\right)-{l}_{1}-2.5{l}_{<0}+\frac{1}{2}{\sum }_{i=1}^{C}{\sum }_{j=i+1}^{C}{d}_{ij}$$

where ${l}_{<0}$ represents the total length of a special type of subregions with coverage 0 in which the contigs aligned to its left and right belong to the same connected component in contig-level assembly graph. Since these types of subregions should not exist, we give them a higher penalty. ${l}_{0}$ represents the total length of other subregions with coverage 0.

The genetic algorithm proceeds in the following steps:

1. Initialization: randomly generate 500 individuals
2. Termination: calculate the adaptivity (objective function) for each individual; the algorithm terminates if either the 10 individuals with the highest adaptivity have the same adaptivity or if the algorithm has been executed for a maximum number of iterations (200)
3. Natural selection: select 60 individuals from the pool of 500 according to their adaptivity (individuals with higher adaptivity are selected with higher probability)
4. Switching: select two individuals from the pool of 60 at random, and use them to generate two offspring individuals by randomly switching their alleles (the alignment position of the contig) at one same locus (contig)
5. Variation: for each of the offspring individual, add a mutation with a probability of 0.05 by either deleting one locus (contig) and its corresponding allele (the alignment position of the contig) or adding one locus (contig) and one of its allele (one of the candidate alignment positions of the contig)
6. Repeat (4–5) until 300 offspring individuals are generated, and then go to (2) for the next iteration

After the termination, TRFill selects the subset of contigs and the combination of candidate alignment positions of contigs in the subset from the individual with the highest adaptivity.

### Phasing

Once TRFill has determined the positions of contigs on the reference, the final step is to generate the sequence for the query TOI. For a haploid genome, the contigs are directly connected into scaffolds by adding a fixed number of “*N*”s. For a diploid genome, the phasing process uses HiFi and Hi-C reads (and the homology between the contigs). Since HiFi and Hi-C provide linkage information with different resolutions, TRFill uses them in different ways. HiFi reads are used to link the contigs belonging to the same haplotypes, while Hi-C reads are used to link the contigs and the genomic regions flanking the TOI in the query genome (hereafter called *shores*). To obtain allele-specific Hi-C information, we only consider Hi-C pairs with both ends on unique *k*-mers (i.e., *k*-mers appearing only once on contigs and shores, *k* = 31). Hi-C reads are positioned on the contigs and the shores according to their unique *k*-mers rather than based on sequence alignment. HiFi signals are instead obtained by aligning the HiFi reads to the contigs using winnowmap2, rather than using unique *k*-mers. We expect that the number of HiFi reads linking contigs on the same haplotypes are much higher than the ones incorrectly linking contigs on different haplotypes. We also expect that contigs with higher homology should belong to the same haplotype with lower probability. The *homology coefficient* between each pair of contigs is computed from the number of shared *k*-mers. If the set of all k-mer in contig $i$ is ${S}_{i}$, and the *homology coefficient* between contig $i$ and contig $j$ is ${h}_{i j}=\frac{\left|{S}_{i} \wedge {S}_{j}\right|}{min(\left|{S}_{i}\right|,\left|{S}_{j}\right|)}$. The *global homology coefficient* $h$ is the average of all non-zero values of ${h}_{i j}$.

Given the HiFi and Hi-C signals and the homology coefficients, we set up a combinatorial optimization framework to solve the phasing problem. Let ${L}_{i}$ be the length of sequence $i$ (contig or shore); $\underline{H}$ be the average value of the homology coefficients for all pairs of contigs; ${MAT}_{shore}$, ${PAT}_{shore}$, ${MAT}_{contig}$, ${PAT}_{contig}$ be the sets of shores and contigs assigned to the maternal and paternal haplotypes (collapsed contigs will appear in both ${MAT}_{contig}$ and ${PAT}_{contig}$); ${HIC}_{ij}, {HIFI}_{ij}, {D}_{ij}, {H}_{ij}$ be the number of Hi-C signals, the number of HiFi signals, the distance, the homology coefficient between sequence $i$ and sequence $j$, respectively. The objective function is as follows.

$$f=C_1\left(\sum\nolimits_i^{i\in{MAT}_{shore}}\sum\nolimits_j^{j\in{MAT}_{contig}}\frac{{HIC}_{ij}}{L_iL_jD_{ij}}+\sum\nolimits_i^{i\in{PAT}_{shore}}\sum\nolimits_j^{j\in{PAT}_{contig}}\frac{{HIC}_{ij}}{L_iL_jD_{ij}}\right)+C_2\left(\sum\nolimits_i^{i\in{MAT}_{contig}}\sum\nolimits_{j=i+1}^{j\in{MAT}_{contig}}\frac{{\left(\underline H-H_{ij}\right)HIFI}_{ij}}{L_iL_j}+\sum\nolimits_i^{i\in{PAT}_{contig}}\sum\nolimits_{j=i+1}^{j\in{PAT}_{contig}}\frac{{\left(\underline H-H_{ij}\right)HIFI}_{ij}}{L_iL_j}\right)-{C}_{3}\frac{|{\sum }_{i}^{{MAT}_{contig}}{L}_{i}-{\sum }_{i}^{{PAT}_{contig}}{L}_{i}|}{{L}_{TOI\_ref}}$$

The objective function has three components, weighted by parameters *C*_1_, *C*_2_, and *C*_3_. The first component rewards Hi-C signals linking the shores and contigs on the same haplotypes. The second component captures two distinct situations. When $\underline{H}-{H}_{ij}$ is positive (i.e., there is low homology between contig $i$ and $j$), then ${HIFI}_{ij}$ contributes positively to the objective function thus rewarding HiFi signals connecting the contigs in the same haplotypes. A higher value of ${H}_{ij}$ leads to a lower $\underline{H}-{H}_{ij}$ which results in a lower score of objective function, representing the negative effect on the homology. When $\underline{H}-{H}_{ij}$ is negative (i.e., there is high homology between contig $i$ and $j$), ${HIFI}_{ij}$ contributes negatively to the objective function. The structure of the second component deals with homologous contigs from different haplotypes which may have HiFi reads connecting them due to the high sequence similarity of the underlying repetitive sequence. The third component penalizes the length difference between the assembled maternal and paternal TOI sequences.

To maximize this complex objective function within an acceptable time, TRFill uses an algorithm inspired by simulated annealing. Each contig can be in one of four states: 0 (discarded), 1 (maternal), 2 (paternal), or 3 (collapsed). The algorithm proceeds as follows.

1. Initialization: randomly set the state of each contig; set the global maximum to a very large negative number
2. Termination: calculate the value of objective function; if this step has been executed for 1000 times, exit
3. Change the state of a random contig if it improves the value of objective function
4. Repeat step (3) until the value of objective function remains unchanged for 100 consecutive iterations and thus a local maximum is obtained; if the local maximum is higher than the current global maximum, set it as the new global maximum
5. Randomly change the states of a fraction of the contigs (50%) to prevent the algorithm from getting stuck from on maxima. Go to step (2)

The final assembly may include some portions of the flanking sequences (shores) because TRFill might assemble reads that overlap the shores. To correct for this issue, TRFill trims these sequences based on an alignment of the final scaffolds against the shores. Finally, the repetitive regions (TOIs) in each haplotype are replaced by the corresponding trimmed scaffolds.

### Evaluation on human centromeric alpha satellite arrays

TRFill was tested on a diploid human genome HG002 (NA24385). The HiFi data (~ 36 × coverage) and Hi-C data (~ 69 × coverage) for this sample was generated by the Human Pangenome Reference Consortium (HPRC) [28]. The corresponding haplotype-resolved high-quality assembly was used as the “ground truth.”

First, we generated a chromosome-level diploid assembly of HG002 to provide as input to TRFill. We used hifiasm (v0.19.5-r587) on HiFi and Hi-C data with default parameters to produce a haplotype-resolved assembly. We used 3D-DNA (branch 201,008) with the “-r 0” option for scaffolding, then manual curation based on Hi-C heap map via Juicebox [44].

The human haploid T2T genome (CHM13 v2.0) was used to guide our assembly of HG002. The positions of the TOIs on HG002 were lifted from the positions of centromere alpha satellite sequences on CHM13, as follows. First, the position of centromere alpha satellite sequence on each chromosome on CHM13 was determined from the downloaded annotations and the results from Tandem Repeat Finder (TRF, v4.09.1) [45]. When a chromosome of CHM13 contained multiple pieces of the alpha satellite sequences, only the longest continuous alpha satellite sequence was considered. SyRI (v1.6) with default parameters was used to build the synteny between CHM13 and the two haplotypes of HG002. The regions of HG002 that aligned to the selected alpha satellite sequences on CHM13 were chosen as the TOI. If the TOI were partially assembled in the chromosome-level assembly of HG002, we removed them and replaced them with a continuous gap for each TOI. We used TRFill, LR_Gapcloser, and SAMBA (v4.1.0) to reassemble the TOIs. TRFill used the CHM13 assembly as reference.

Several criteria were used to comparatively evaluate the quality of the assemblies TRFill, LR_Gapcloser, and SAMBA in terms of contiguity, completeness, and accuracy. First, the TOI assemblies were aligned to the ground truth using winnowmap2. Any region corresponding to a continuous alignment was considered a *correct* genomic region. We defined *completeness* as the ratio between the total length of the correct regions and the total length of the ground truth. We defined *correctness* as the ratio between the total length of correct regions and the total length of assembly. We defined the *LIS score* as the ratio between (i) the length of the longest increasing subsequence between the unique *k*-mers in the ground truth assembly and the TOI assembly and (ii) the number of unique *k*-mers in the TOI assembly (see section Determining Positions and Orientations of Contigs in Methods for the generation of LIS).

The *strictly-improved* and *loosely-improved* criteria are defined based on the R_AQI and S_AQI scores generated by CRAQ, as well as the HiFi read coverages for both the TRFill and the original assemblies. The R_AQI and S_AQI scores were obtained by running CRAQ with either the TRFill or original assembly, along with the whole-genome HiFi reads as input, using the “-x map-hifi” parameter. HiFi read coverage was calculated by aligning the whole-genome HiFi reads to the respective assemblies using winnowmap2 with default settings.

First, let R_AQI_ori, S_AQI_ori, R_AQI_new, S_AQI_new, cov_ori, and cov_new represent the R_AQI, S_AQI, and HiFi read coverage for the original and TRFill assemblies, respectively. Let cov_whole denote the average genome-wide HiFi read coverage. A gap is considered *strictly improved* if R_AQI_new > R_AQI_ori × 1.1. A gap is considered *loosely improved* if R_AQI_new > R_AQI_ori, or if the conditions (1–3) are met. Condition (1) R_AQI_new > R_AQI_ori/1.6; condition (2) S_AQI_new > S_AQI_ori; condition (3) |cov_whole – cov_new| <|cov_whole – cov_ori|.

### Evaluation on tomato subtelomeric tandem repeats

For the analysis of tomato subtelomeric tandem repeats, we built a comprehensive dataset for Heinz1706, TS2, and TS281 using publicly available data and our own sequencing. We generated ONT UL sequencing data for all three, and Illumina Hi-C sequencing for TS2 and TS281. We were able to obtain Hi-C data for Heinz1706 and HiFi data for all three on public repositories. Our sequencing sample was collected from young leaves of a tomato plant at the Agricultural Genomics Institute at Shenzhen, Chinese Academy of Agricultural Sciences (Guangdong province, China). For the ONT UL sequencing, high-molecular-weight genomic DNA selected with SageHLS HMW library system (Sage Science). DNA was processed with the Ligation sequencing 1D Kit. The library was constructed using the approach described in Wang et al. and sequenced on the Nanopore PromethION platform at the Genome Center of Grandomics (Wuhan, China). For the Hi-C sequencing, the library was constructed and sequenced using the approach described in Rao et al. and sequenced using the Illumina NovaSeqX-plus platform at the Genome Center of Grandomics (Wuhan, China). The restriction enzyme used for Hi-C library construction is DpnII from NEB.

To build the reference genome (Heinz1706) and two ground truth genomes (TS2 and TS281), we assembled them individually de novo. We used hifiasm (v0.19.5-r587) with the “-ul” and “-l0” options to generate contigs based on HiFi and ONT UL reads. We used 3D-DNA (branch 201,008) with the “-r 0” option for scaffolding using Hi-C reads.

In the experiments for the haploid genome, the subtelomeric tandem repetitive regions of TS2 and TS281 were the TOIs that were reassembled with TRFill (guided by the Heinz1706 reference genome). The chromosome-level assembly of TS2 and TS281 was obtained from SolOmics (http://solomics.agis.org.cn/tomato/ftp). These assemblies were generated exclusively from HiFi data. TRFill was used to assemble the TOIs.

In the experiments on the diploid genomes, the TS2 and TS281 haplotype assemblies were merged to produce a pseudo-diploid genome. The HiFi and Hi-C data for both TS2 and TS281 were merged to simulate data for the pseudo-diploid genome. Since the merged Hi-C data lacked the inter-chromosome read pairs between the maternal and paternal chromosomes, additional Hi-C read pairs were added into the Hi-C data set. We used sim3C [46] with default parameters to generate synthetic Hi-C reads at 130 × sequencing depth using the ground truth genome as the reference. Synthetic read pairs between maternal and paternal chromosomes were selected and added to the real Hi-C reads.

The chromosome-level assembly of the diploid genome was generated using hifiasm (v0.19.5-r587) using HiFi and Hi-C data. Scaffolding was carried out with 3D-DNA using Hi-C data.

The subtelomeric tandem repeats (query TOI regions) were identified using the reference genome. TRF (v4.09.1) was used to detect repetitive patterns within the reference sequences. We identified regions located at the edge of each chromosome that harbor 181 bp-long monomers. Not every chromosome had both pairs of subtelomeric repeats, and some chromosomes contained the 181 bp monomers not at the chromosome ends. The TOI regions on the query were identified using the synteny between the reference and the query determined by SyRI (v1.6).

### Population-level analysis of tomato subtelomeric tandem repeat units

Chromosome-level assemblies for 29 tomato genomes were downloaded from SolOmics (http://solomics.agis.org.cn/tomato/ftp). The subtelomeric tandem repetitive regions were assembled by TRFill with the same pipeline as the validation experiments of the haploid tomato genome using Heinz1706 as the reference. For each subtelomeric tandem repeat, if the TRFill assembly was longer than the original one, we kept the TRFill assembly (the original sequence was kept otherwise).

The population-level analysis used the Tandem Repeat Annotation and Structural Hierarchy (TRASH) [47]. Both monomer mode and HOR mode were used in TRASH to detect the monomers and HORs from the tandem repeat sequences, providing coordinates, orientation, and length of each repeat unit.

For the analysis of monomers and HORs, the 181 bp consensus sequence of the monomer was built by carrying out a multiple sequence alignment. A consensus monomer sequence for each subtelomere was built according to sequence alignment of all the monomers in each subtelomere (using mafft2 [48]). The global consensus sequence was generated by a multiple sequence alignment (again, using mafft2) of all subtelomere-level consensus sequences.

To analyze the sequence similarity of monomers, we carried out comparisons between (1) the intra-genome monomers and inter-genome monomers, (2) intra-chromosome and inter-chromosome monomers on the same genomes, and (3) intra-subtelomere and inter-subtelomere monomers on the same chromosomes. For each of these comparisons, a series of experiments were carried out, each of which computed the pairwise similarity between a randomly selected pair of monomers. The pairwise similarity was defined as the ratio between the number of common monomers in the two sets and the total number of monomers in both sets. For the random experiments (1) and (2), each of the two sets contained all monomers from a randomly selected subtelomere. For the random experiments in (3), each of the two sets contained one tenth of the monomers in a randomly selected subtelomere, and the two sets did not contain the same monomers for intra-experiments. The similarity calculations between all pairs of subtelomeres was defined in the same way.

## Supplementary Information

Additional file 1.

Additional file 2.

Additional file 3.

## Data Availability

The code of TRFill is available at its GitHub repository https://github.com/panlab-bioinfo/TRFill [49] and Zenodo 10.5281/zenodo.15719141 [50] under the Apache License 2.0. All sequencing data for the tomato genomes generated in this study are available in the Genome Sequence Archive (GSA; https://ngdc.cncb.ac.cn/gsa/) of the National Genomics Data Center (NGDC; https://ngdc.cncb.ac.cn/bioproject/) under BioProject accession number PRJCA031197 [51]. Specifically: The tomato TS281 Hi-C reads and ONT-UL reads are available at GSA with the accession number CRA019923 [52]. The tomato TS2 Hi-C reads and ONT-UL reads are available at GSA with the accession number CRA019767 [53]. The tomato Heinz1706 ONT-UL reads are available at GSA with the accession number CRA019706 [54]. Additionally, the public tomato pan-genomes data used in this study are available at NCBI BioProject (https://www.ncbi.nlm.nih.gov/bioproject/) with the accession numbers are PRJNA733299 [55] and PRJNA756391 [56]. The public human data used in this study are available at https://github.com/marbl/CHM13 [57] (CHM13 genome) and https://github.com/marbl/HG002/blob/main/Sequencing_data.md [58] (HG002 genome and sequence data).
