# Supplementary material for: TRFill: synergistic use of HiFi and Hi-C sequencing enables accurate assembly of tandem repeats for population-level analysis
Source: Genome Biol. 2025 Jul 28;26:227. doi: 10.1186/s13059-025-03685-5 (PMC12305924; doi:10.1186/s13059-025-03685-5)
Supplement: Supplementary file 1 — Additional file 1. [file 13059_2025_3685_MOESM1_ESM.docx]

**TRFill: synergistic use of HiFi and Hi-C sequencing enables accurate assembly of tandem repeats for population-level analysis**

**Authors**

Huaming Wen^1,2,†^, Jinbao Yang^2,†^, Xianjia Zhao^2^, Xingbin Wang^2^, Jiawei Lei^2^, Yanchun Li^4^, Wenjie Du^2^, Dongxi Li^4^, Yun Xu^1^, Stefano Lonardi^3, *^, Weihua Pan^2, *^

**Affiliations**

^1^School of Computer Science and Technology, University of Science and Technology of China, Hefei, 230027, China

^2^State Key Laboratory of Genome and Multi-omics Technologies，Shenzhen Branch, Guangdong Laboratory for Lingnan Modern Agriculture, Genome Analysis Laboratory of the Ministry of Agriculture and Rural Affairs, Agricultural Genomics Institute at Shenzhen, Chinese Academy of Agricultural Sciences, Shenzhen 518120, China

^3^Department of Computer Science and Engineering, University of California, Riverside, CA 92521, USA

^4^College of Computer Science and Technology, Taiyuan University of Technology, Taiyuan 030024, China

^†^These authors contributed equally: Huaming Wen, Jinbao Yang

*Corresponding authors

Weihua Pan - Email: panweihua@caas.cn

Stefano Lonardi - Email: stelo@cs.ucr.edu

**Case Analysis of Time and Cost in T2T Genome and Pangenome Construction**

Here, we use the haploid T2T genome CHM13, assembled by the T2T Consortium, and the near-T2T diploid genome HG002, assembled by the Human Pangenome Reference Consortium, as examples to analyze the challenges faced by small research groups in constructing large-scale T2T genomes and T2T pangenomes, in terms of both sequencing costs and workload.

First, using current sequencing prices in mainland China—which are generally lower than those in most other regions—we estimate the cost of constructing a T2T genome for the HG002 sample. The HG002 project utilized three cells of PacBio HiFi Revio sequencing, costing 3 × RMB ¥12,500 = RMB ¥37,500. For 40× ONT ultra-long (UL) reads with an N50 of 91 kb, the estimated cost is 40 × 3.1 Gb / 10 Gb × RMB ¥10,000 = RMB ¥124,000 (assuming 10 Gb of data per sequencing cell at RMB ¥10,000 per cell). For 120× Hi-C data, the cost includes RMB ¥6,000 for library preparation and 120 × 3.1 Gb × RMB ¥20 per Gb for sequencing, totaling RMB ¥13,740. A 50× WGS (short-read) dataset costs 50 × 3.1 Gb × RMB ¥20 = RMB ¥2,480. Bionano optical mapping data were also used, but the exact amount and cost are currently unknown.

Even excluding the cost of Bionano data, the total sequencing cost amounts to approximately RMB ¥177,720, or around USD $24,347. Notably, ONT UL data accounts for roughly 70% of the total cost. For a small research group, this represents a significant financial burden.

If a T2T pangenome were to be constructed across 100 individuals, the sequencing costs alone would reach USD $2,434,700—a figure that is prohibitively expensive for nearly any research group. Even for large-scale efforts like the Human Pangenome Reference Consortium, such an investment represents a substantial financial commitment.

Second, we consider the time required to construct a T2T genome. The T2T Consortium began assembling the CHM13 genome in the first half of 2019 and completed it by mid-2021, taking approximately two years. A significant portion of this time was dedicated to the manual resolution of complex repetitive regions, such as centromeres. Since CHM13 is a haploid genome, the assembly process was relatively more straightforward. In contrast, achieving T2T assembly for diploid human genomes is substantially more complex and time-consuming.

Notably, this two-year effort was carried out by a consortium comprising some of the world’s leading genome assembly experts. The fact that such a highly skilled team required so much time underscores the difficulty of this task—making it nearly infeasible for most individual biology research groups to undertake.

When scaling to a T2T pangenome involving 100 individuals, the required time and labor become almost unimaginable, posing an enormous challenge even for large consortia.
