# Supplementary material for: TRFill: synergistic use of HiFi and Hi-C sequencing enables accurate assembly of tandem repeats for population-level analysis
Source: Genome Biol. 2025 Jul 28;26:227. doi: 10.1186/s13059-025-03685-5 (PMC12305924; doi:10.1186/s13059-025-03685-5)
Supplement: Supplementary file 2 — Additional file 2. [file 13059_2025_3685_MOESM2_ESM.docx]

**TRFill: synergistic use of HiFi and Hi-C sequencing enables accurate assembly of tandem repeats for population-level analysis**

**Authors**

Huaming Wen^1,2,†^, Jinbao Yang^2,†^, Xianjia Zhao^2^, Xingbin Wang^2^, Jiawei Lei^2^, Yanchun Li^4^, Wenjie Du^2^, Dongxi Li^4^, Yun Xu^1^, Stefano Lonardi^3, *^, Weihua Pan^2, *^

**Affiliations**

^1^School of Computer Science and Technology, University of Science and Technology of China, Hefei, 230027, China

^2^State Key Laboratory of Genome and Multi-omics Technologies，Shenzhen Branch, Guangdong Laboratory for Lingnan Modern Agriculture, Genome Analysis Laboratory of the Ministry of Agriculture and Rural Affairs, Agricultural Genomics Institute at Shenzhen, Chinese Academy of Agricultural Sciences, Shenzhen 518120, China

^3^Department of Computer Science and Engineering, University of California, Riverside, CA 92521, USA

^4^College of Computer Science and Technology, Taiyuan University of Technology, Taiyuan 030024, China

^†^These authors contributed equally: Huaming Wen, Jinbao Yang

*Corresponding authors

Weihua Pan - Email: panweihua@caas.cn

Stefano Lonardi - Email: stelo@cs.ucr.edu


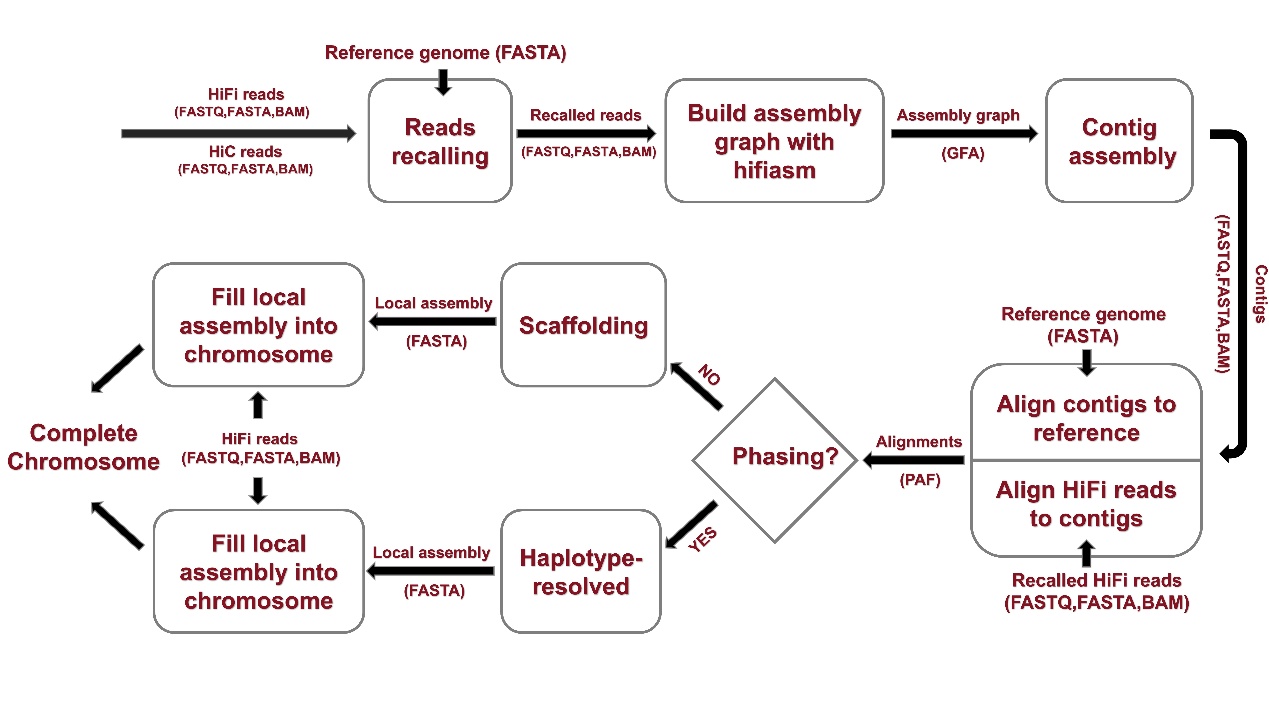


**Fig. S1** Workflow of TRFill software.


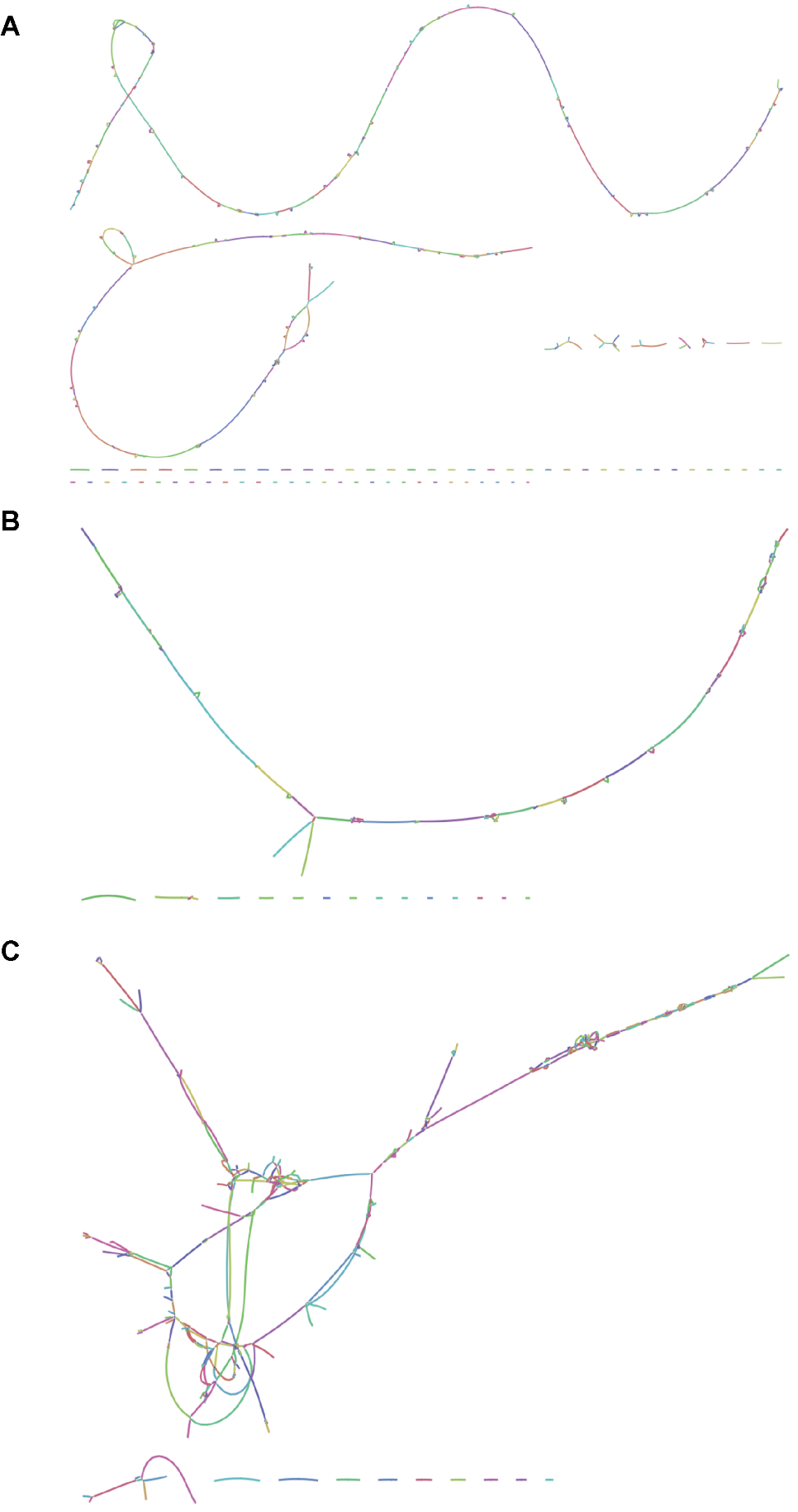


**Fig. S2** The unitig graph for HG002 produced by hifiasm (v0.19.5-r587), and visualized by Bandage (v0.8.1). **(A)** Chromosome 1 **(B)** Chromosome 16 and **(C)** Chromosome 6


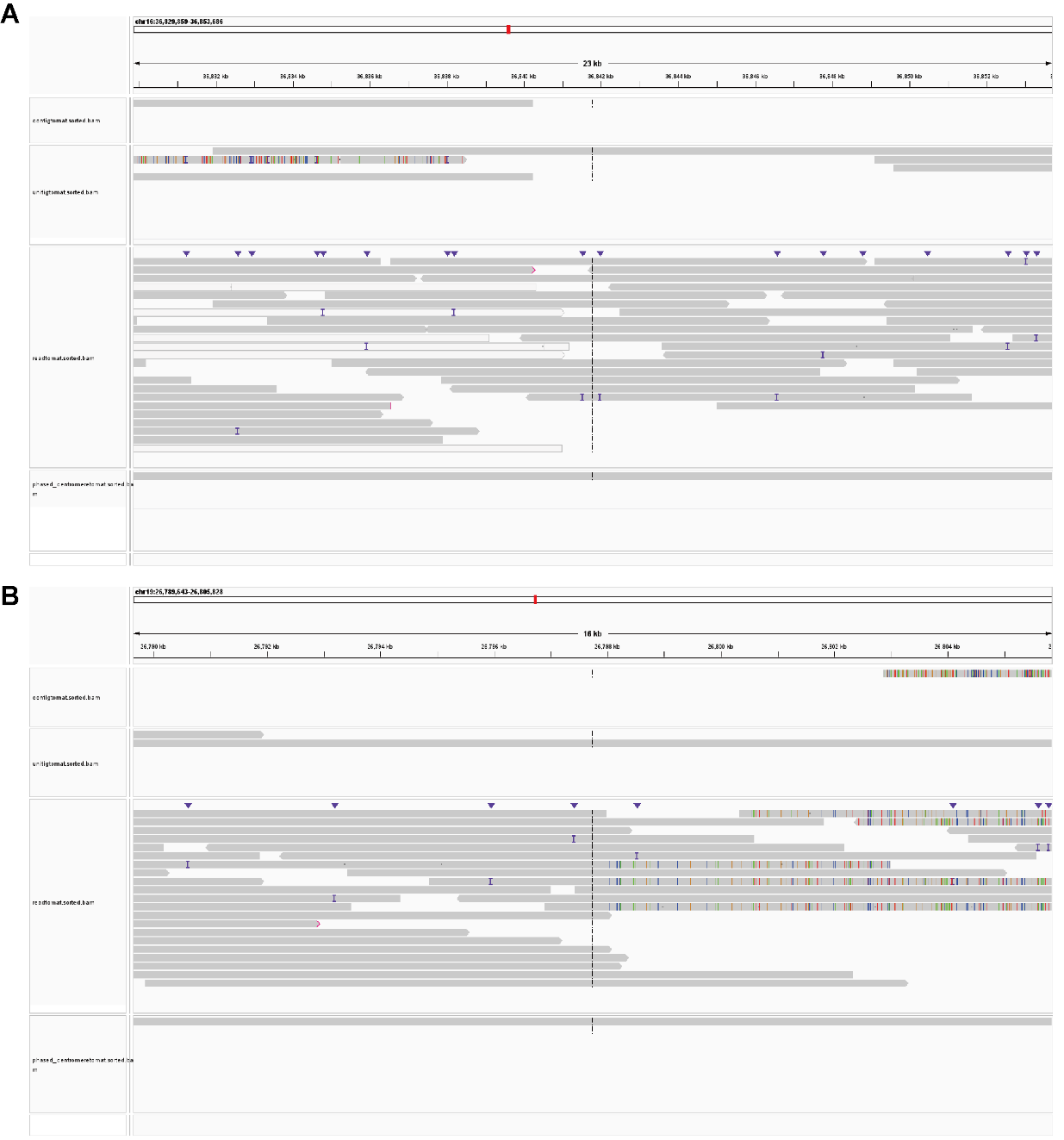


**Fig. S3** Sequence alignment against the ground truth assembly visualized using IGV (v2.13.2) on **(A)** HG002 maternal chromosome 16 36,829,859bp-36,853,686 bp and **(B)** HG002 maternal chromosome 19 26,789,643-26,805,828 bp. The first track refers to the contig graph, the second track refers to the unitig graph, the third track refers to the raw reads and the final track refers to the TRFill assembly.


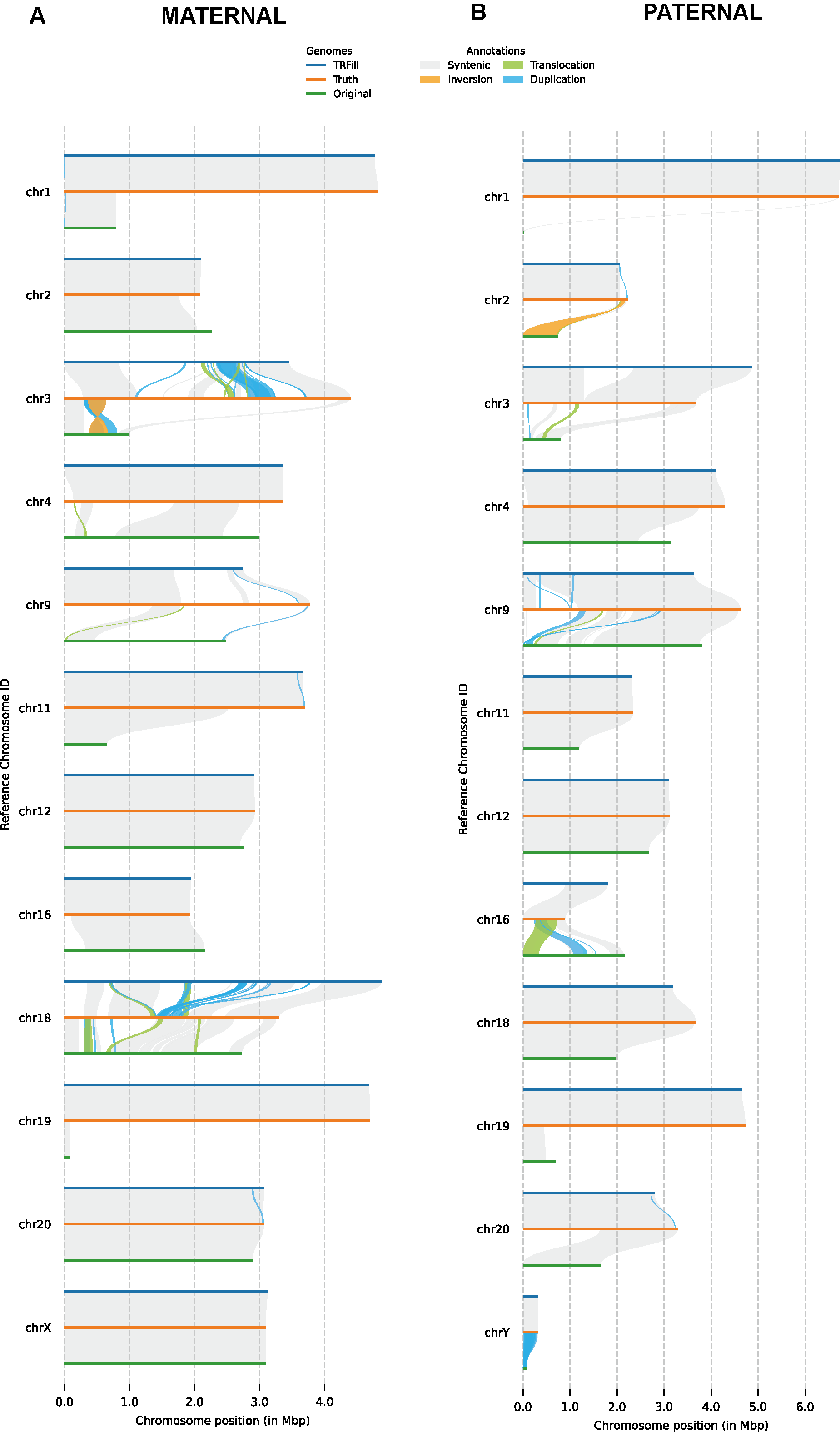


**Fig. S4** SyRI synteny plots between the hifiasm+3Ddna assemblies (original), the TRFill assemblies of alpha satellite sequences and the “ground truth” assemblies for HG002 maternal (left) and paternal (right) chromosomes; acrocentric chromosomes Chr13, Chr14, Chr15, Chr21, and Chr22 are excluded.


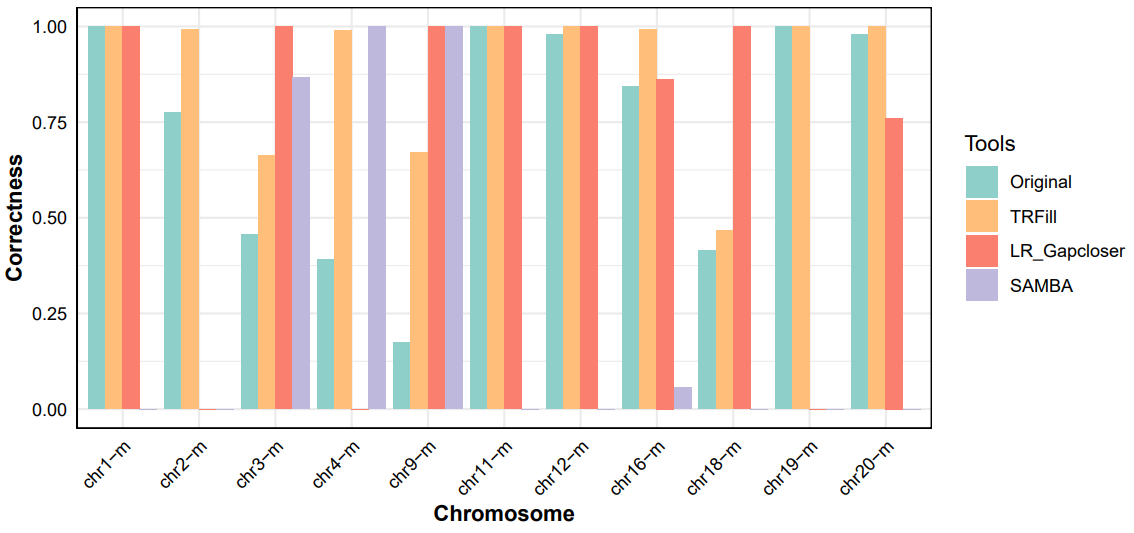


**Fig. S5** Correctness comparison between the hifiasm+3Ddna assemblies (original) and TRFill, LR_Gapcloser and SAMBA assemblies on eleven HG002 maternal chromosomes; acrocentric chromosomes Chr13, Chr14, Chr15, Chr21, and Chr22 are excluded.

**
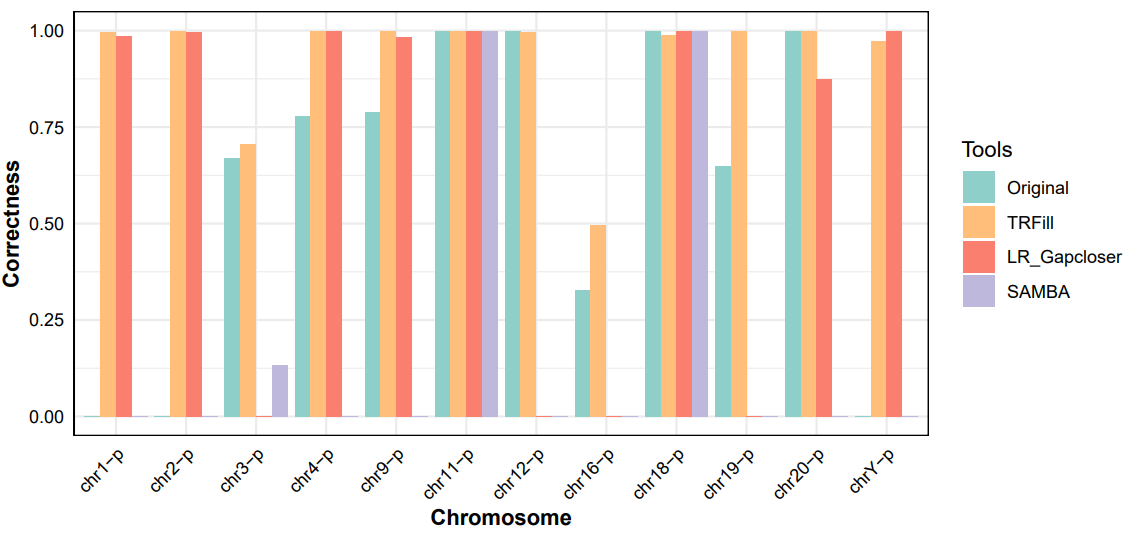
**

**Fig. S6** Correctness comparison between the hifiasm+3Ddna assemblies (original) and TRFill, LR_Gapcloser and SAMBA assemblies on twelve HG002 paternal chromosomes; acrocentric chromosomes Chr13, Chr14, Chr15, Chr21, and Chr22 are excluded.

**
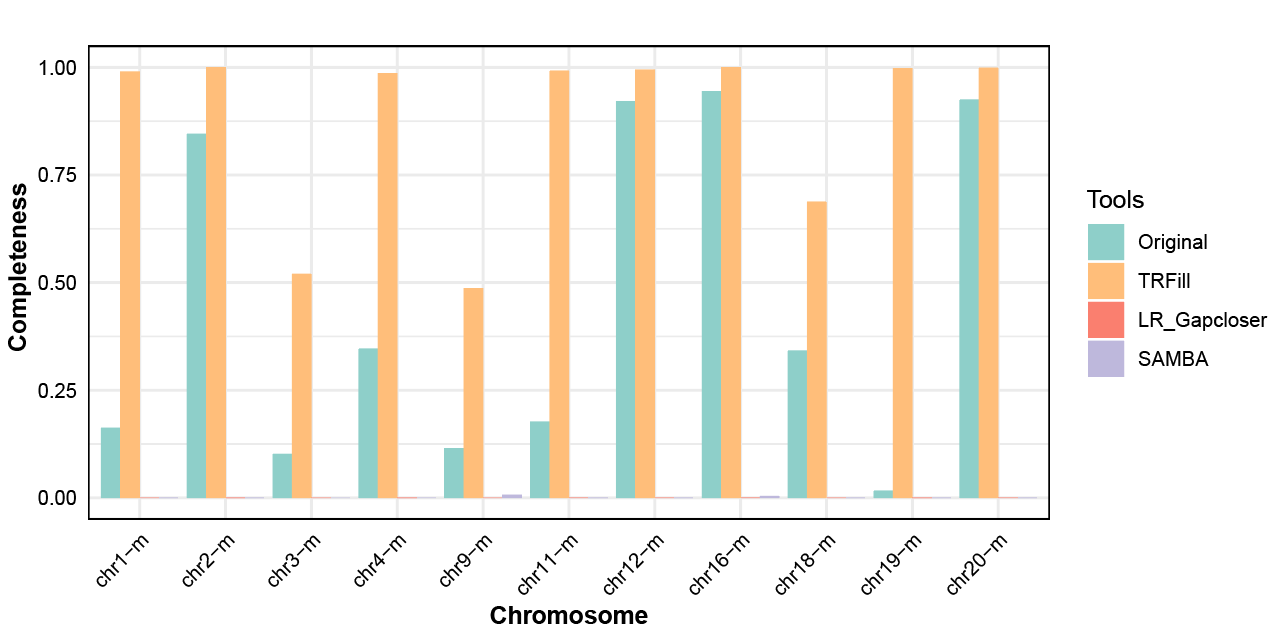
**

**Fig. S7** Completeness comparison between the hifiasm+3Ddna assemblies (original) and TRFill, LR_Gapcloser and SAMBA assemblies on eleven HG002 maternal chromosomes; acrocentric chromosomes Chr13, Chr14, Chr15, Chr21, and Chr22 are excluded.

**
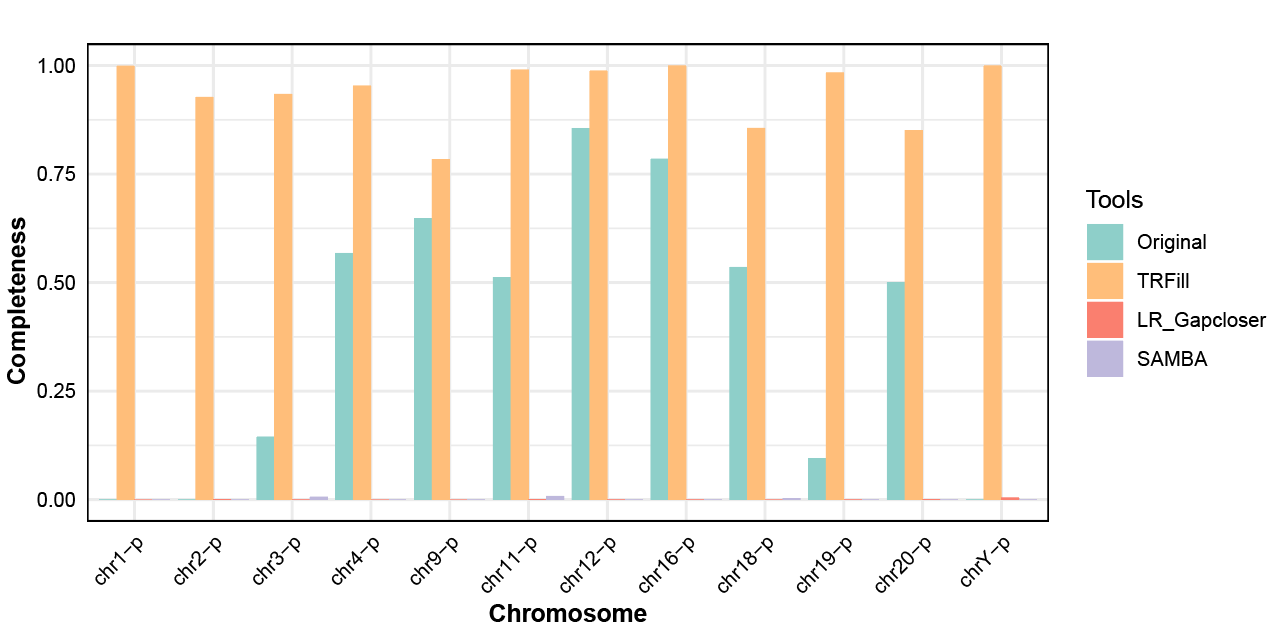
**

**Fig. S8** Completeness comparison between the hifiasm+3Ddna assemblies (original) and TRFill, LR_Gapcloser and SAMBA assemblies on the twelve HG002 paternal chromosomes; acrocentric chromosomes Chr13, Chr14, Chr15, Chr21, and Chr22 are excluded.


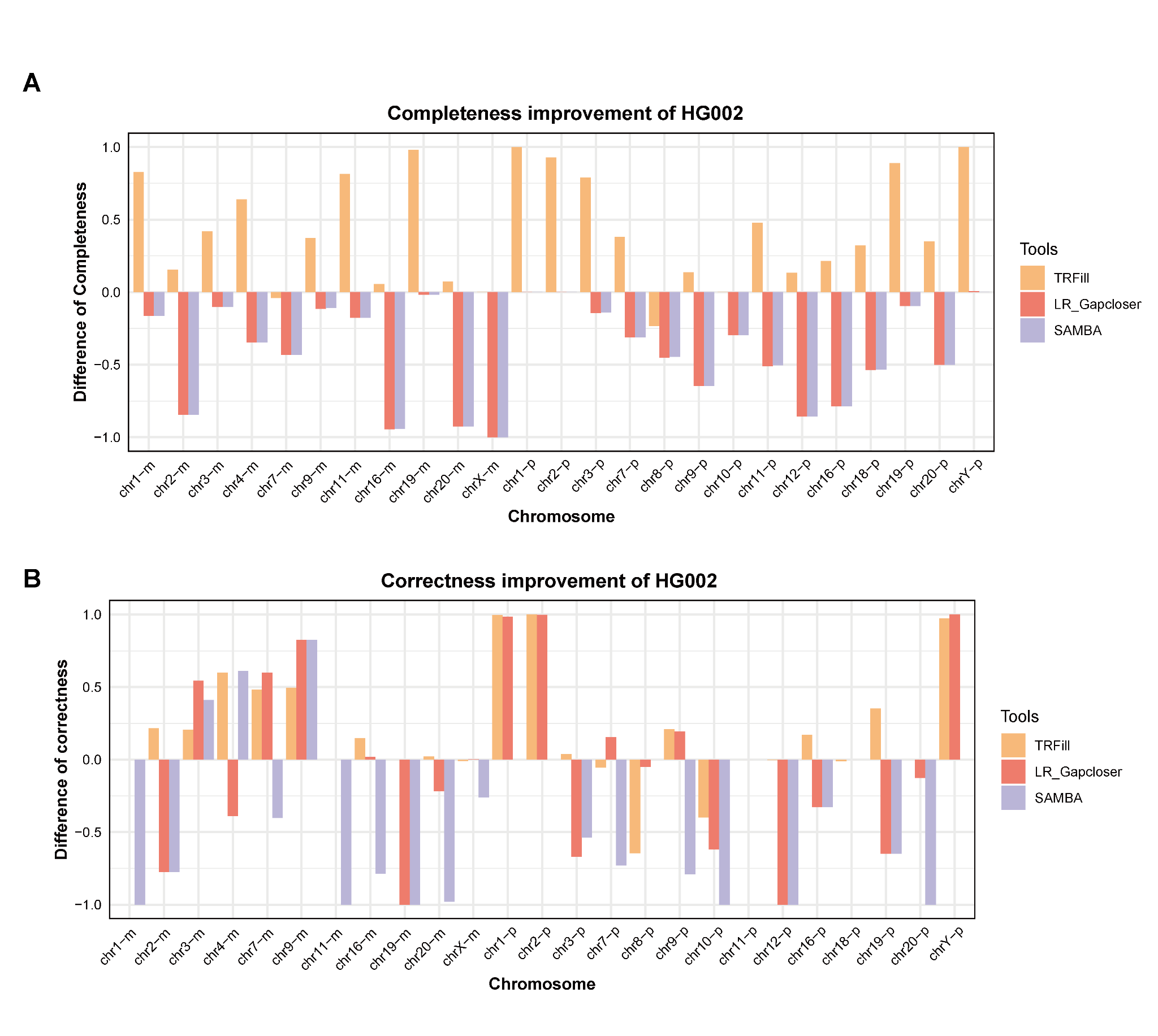


**Fig. S9** Completeness and correctness comparison between the hifiasm+3Ddna assemblies (original) and the TRFill, LR_Gapcloser and SAMBA assemblies on the loosely-identified HG002 maternal and paternal chromosomes.


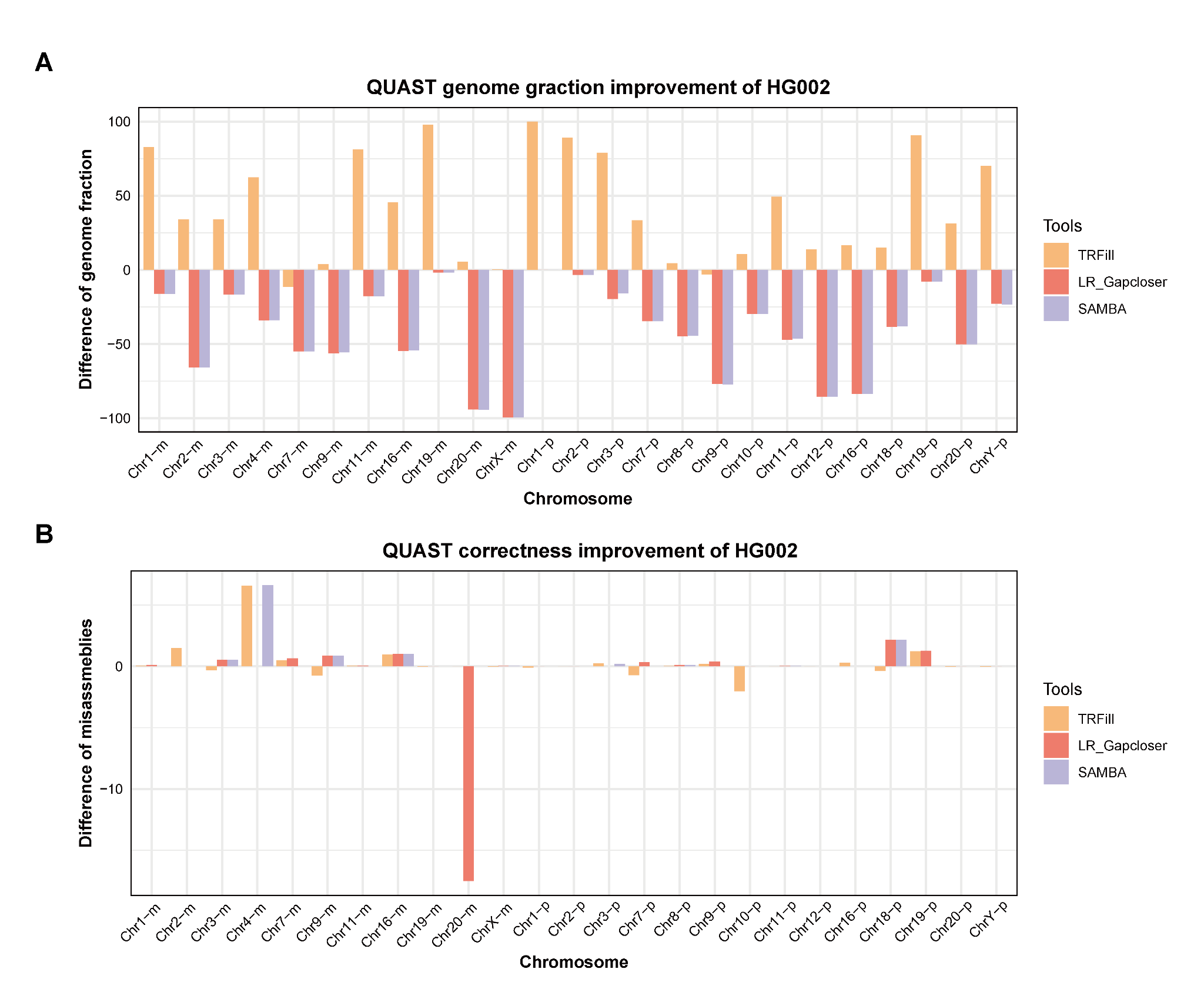


**Fig. S10** QUAST genome fraction and misassembly density comparison between the hifiasm+3Ddna assemblies (original) and TRFill, LR_Gapcloser and SAMBA assemblies on the loosely-identified HG002 maternal and paternal chromosomes.


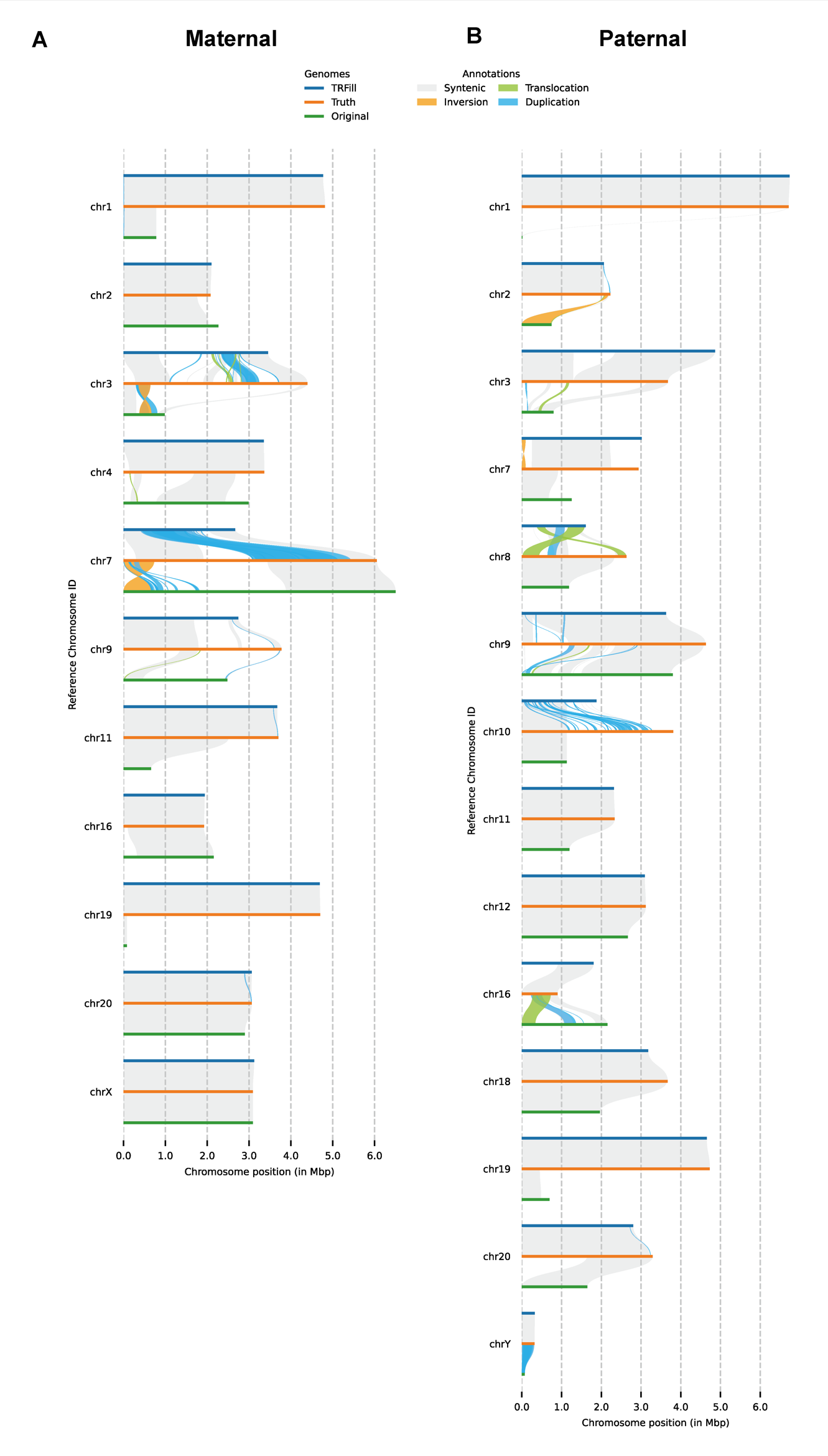


**Fig. S11** Synteny plots generated by SyRI between the hifiasm+3Ddna assemblies (original) assemblies, the assemblies after TRFill reassembly of alpha satellite sequences and the “ground truth” assemblies for the loosely-identified HG002 maternal (left) and paternal (right) chromosomes.


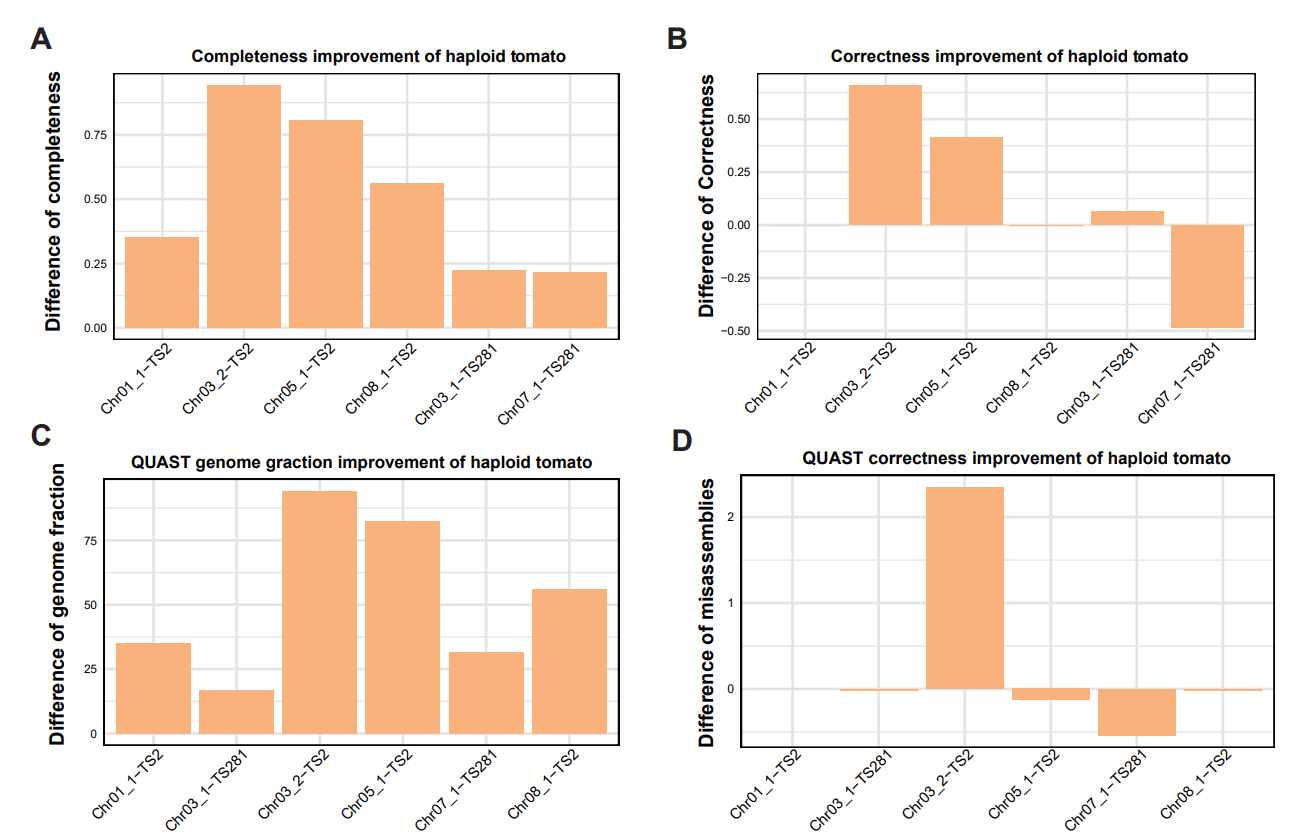


**Fig. S12** Completeness and correctness comparison between the original assemblies and the TRFill assemblies on the strictly-identified chromosomes in the haploid tomato genomes.


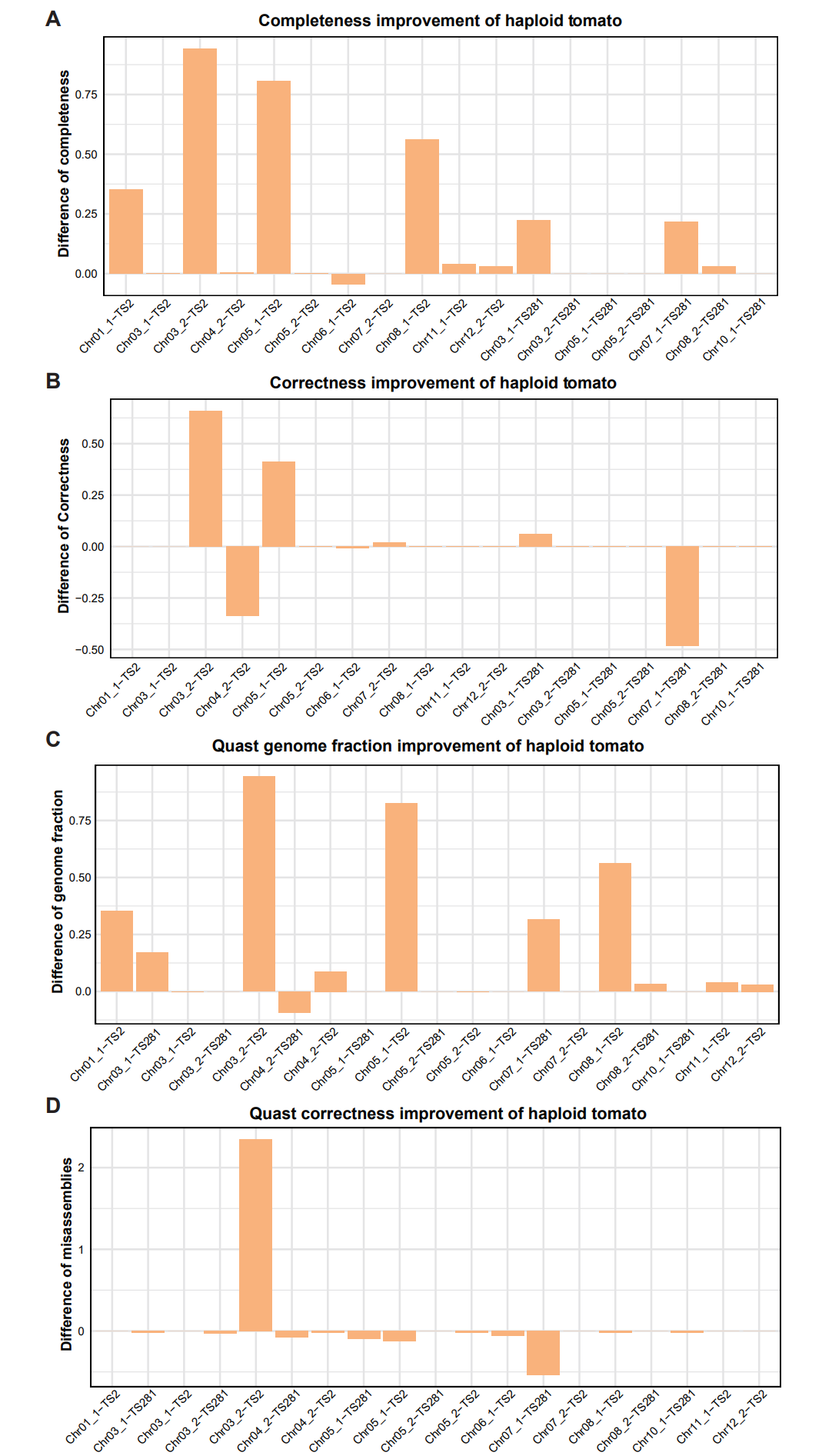


**Fig. S13** Completeness and correctness comparison between the original assemblies and the TRFill assemblies on the loosely-identified chromosomes in the haploid tomato genomes.


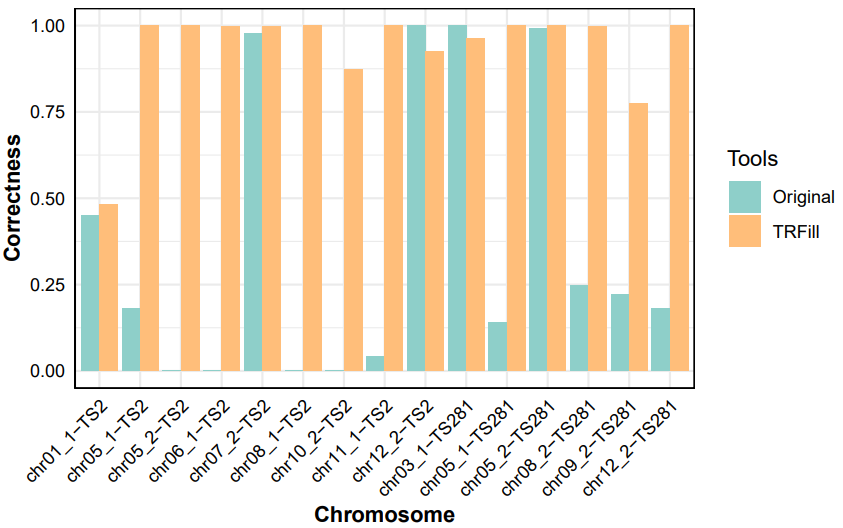


**Fig. S14** Correctness comparison between the hifiasm+3Ddna assemblies (original) and TRFill assemblies on the synthetic diploid tomato genome; only chromosomes with improved TRFill assemblies are shown.


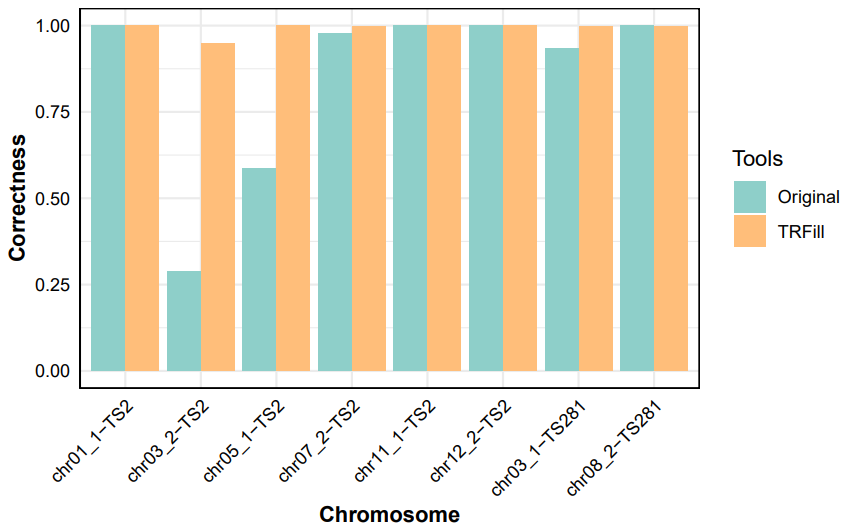


**Fig. S15** Correctness comparison between the hifiasm+3Ddna assemblies (original) and TRFill assemblies on the haploid tomato genomes; only chromosomes with improved TRFill assemblies are shown.


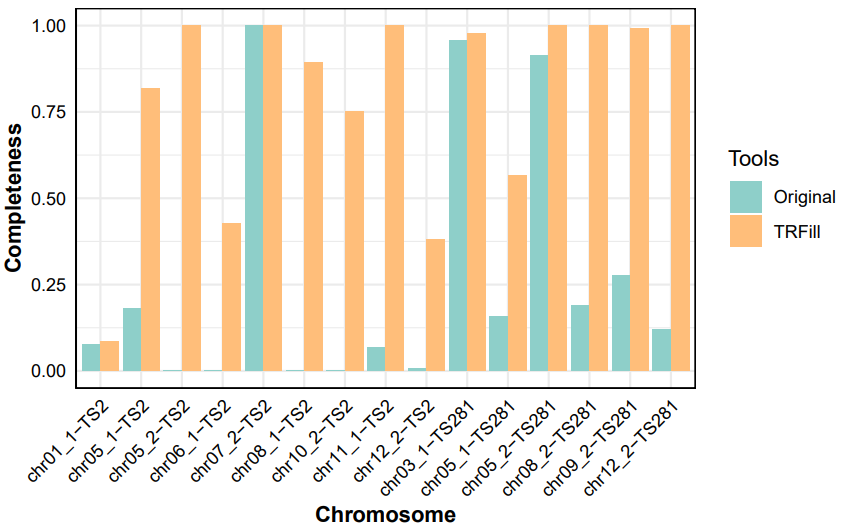


**Fig. S16** Completeness comparison between the hifiasm+3Ddna assemblies (original) and TRFill assemblies on the synthetic diploid tomato genome; only chromosomes with improved TRFill assemblies are shown.


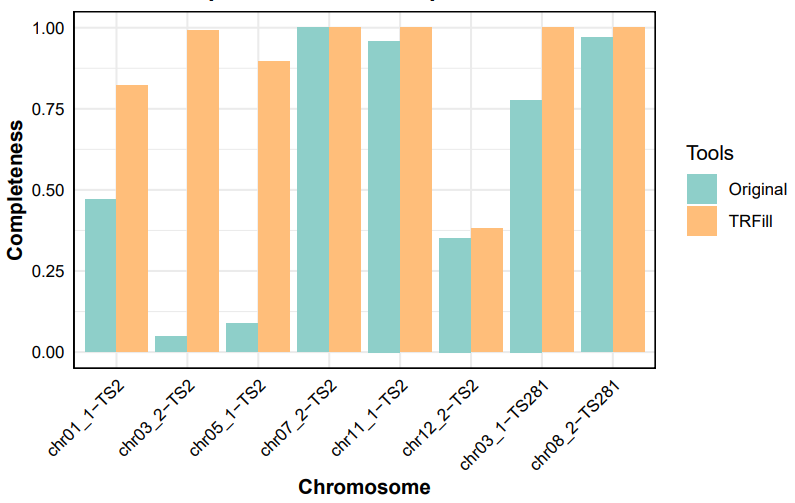


**Fig. S17** Completeness comparison between the hifiasm+3Ddna assemblies (original) and the TRFill assemblies on the haploid tomato genomes; only chromosomes with improved TRFill assemblies are shown.


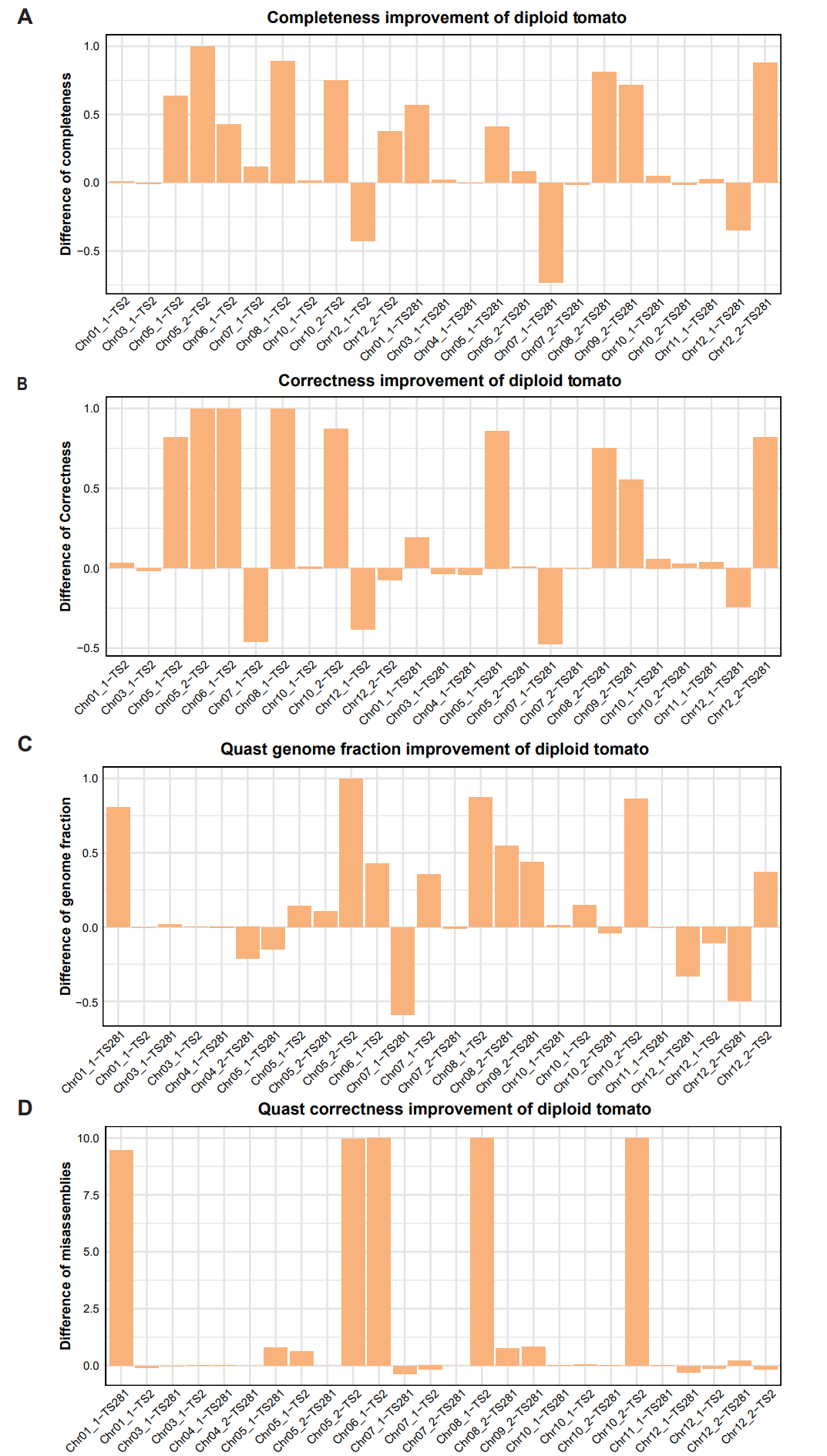


**Fig. S18** Completeness and correctness comparison between the original assemblies and TRFill assemblies on the loosely-identified chromosomes in the synthetic diploid tomato genome.


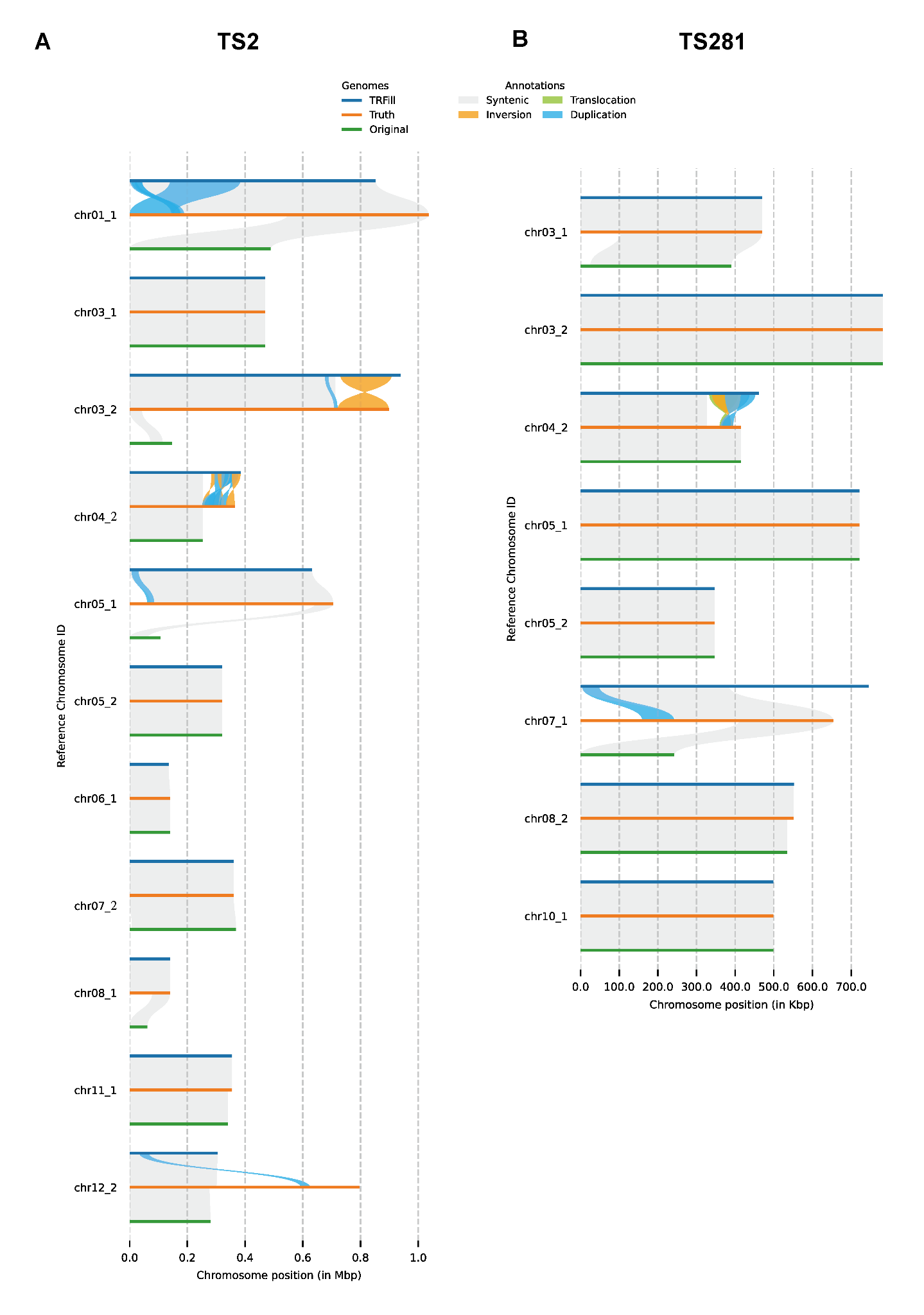


**Fig. S19** SyRI synteny plots comparing the original assemblies, the TRFill assembly of subtelomeric tandem repeats and the “ground truth” assemblies for the loosely-identified chromosomes in the tomato haploid genomes.


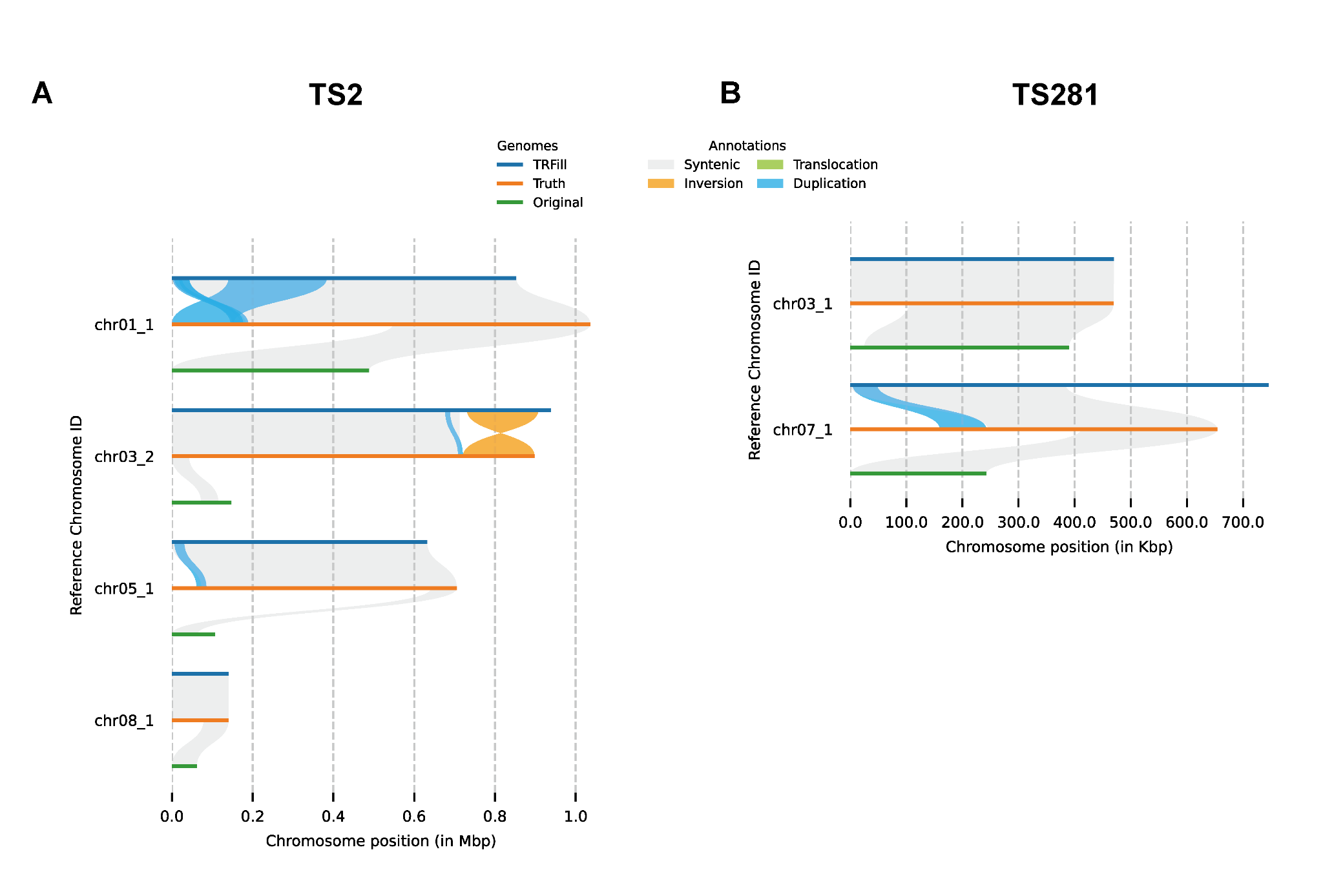


**Fig. S20** SyRI synteny plots comparing the original assemblies, the TRFill assembly of subtelomeric tandem repeats and the “ground truth” assemblies for the strictly-identified chromosomes in the tomato haploid genomes.


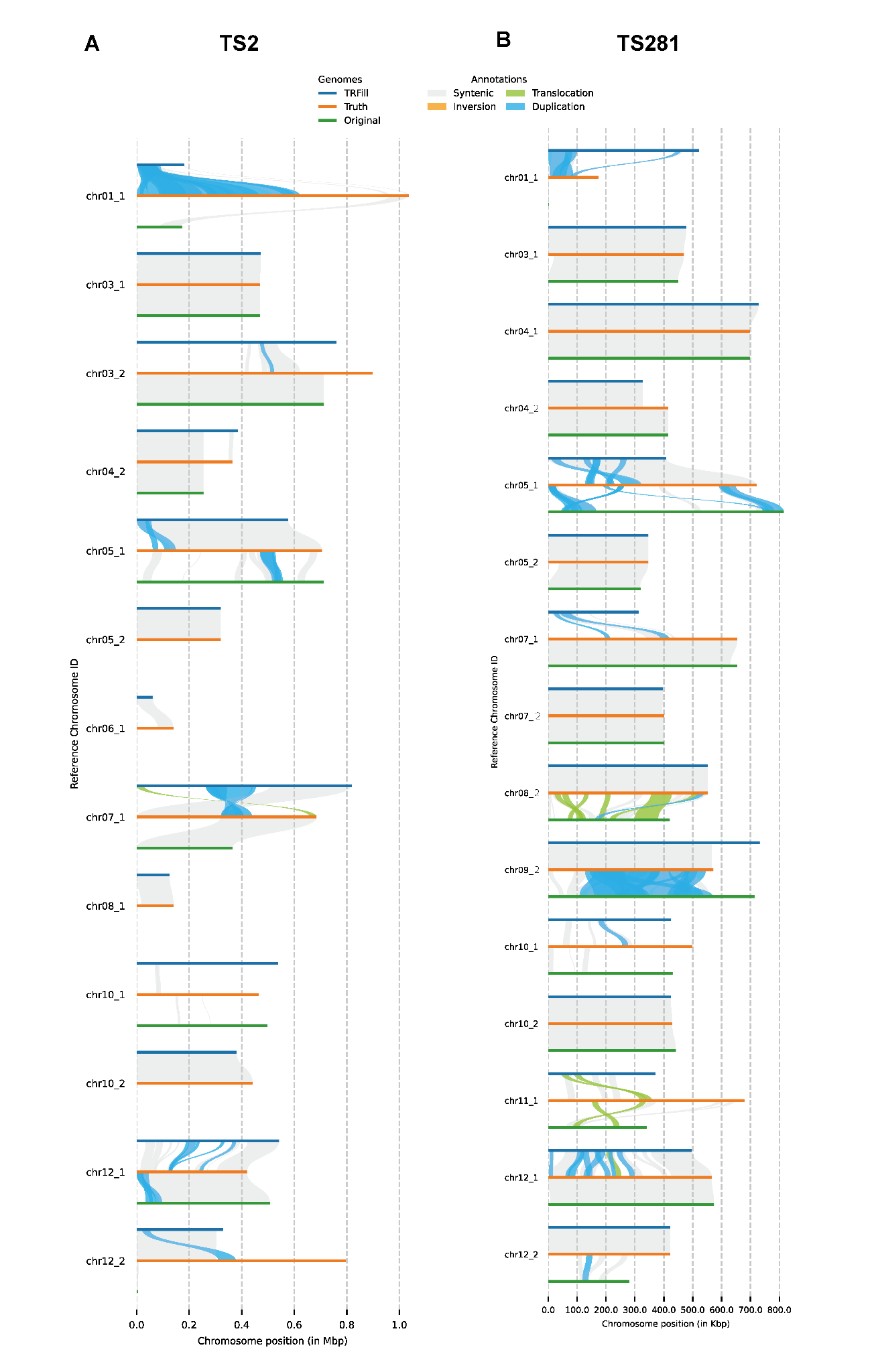


**Fig. S21** SyRI synteny plots comparing the original assemblies, the TRFill assembly of subtelomeric tandem repeats and the “ground truth” assemblies for the loosely-identified chromosomes (TS2 and TS281 as two haplotypes) in the synthetic tomato diploid genome.


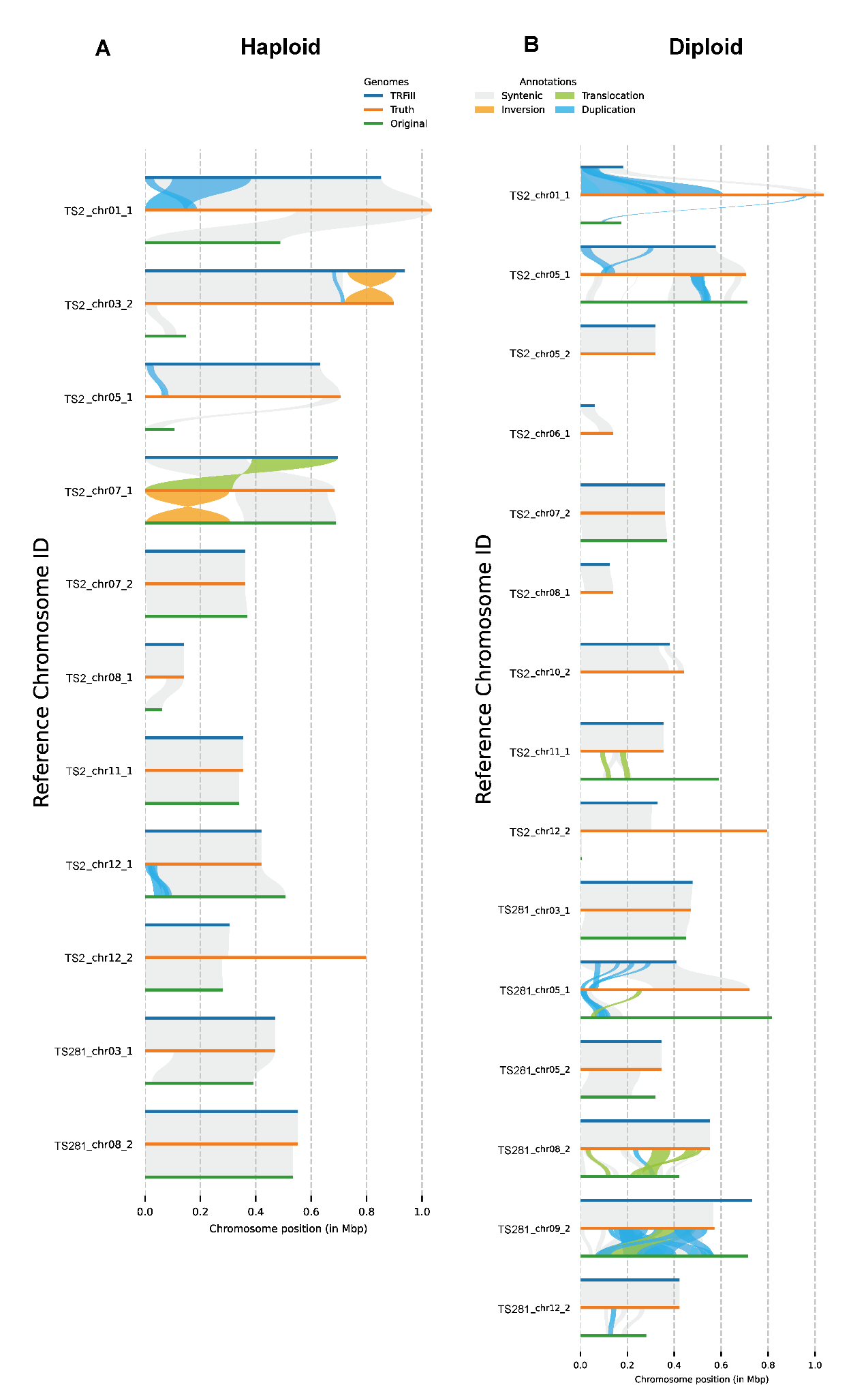


**Fig. S22** SyRI synteny plots between the hifiasm+3Ddna assemblies (original), the TRFill assembly of subtelomeric tandem repeats and the “ground truth” assemblies for tomato haploid (left) and diploid (right) genomes; only chromosomes with improved TRFill assemblies are shown.


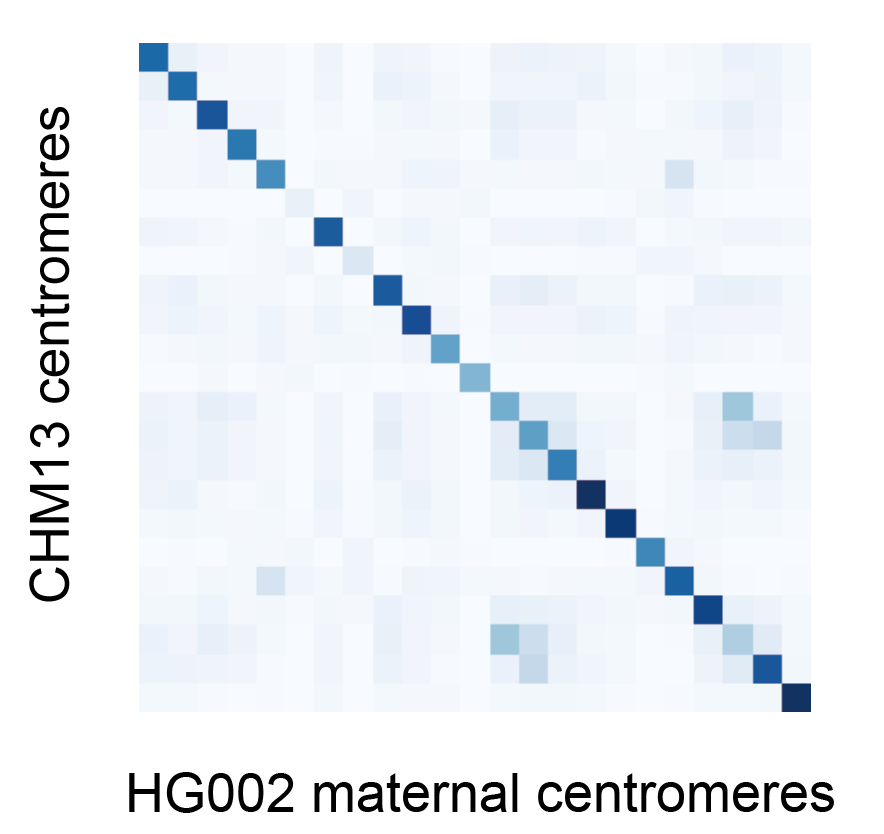


**Fig. S23** This heatmap illustrates the sequence similarity between centromeres of chromosomes in HG002 and CHM13. The diagonal represents the similarity of centromeres with the same chromosome number, with color intensity indicating the degree of sequence similarity.


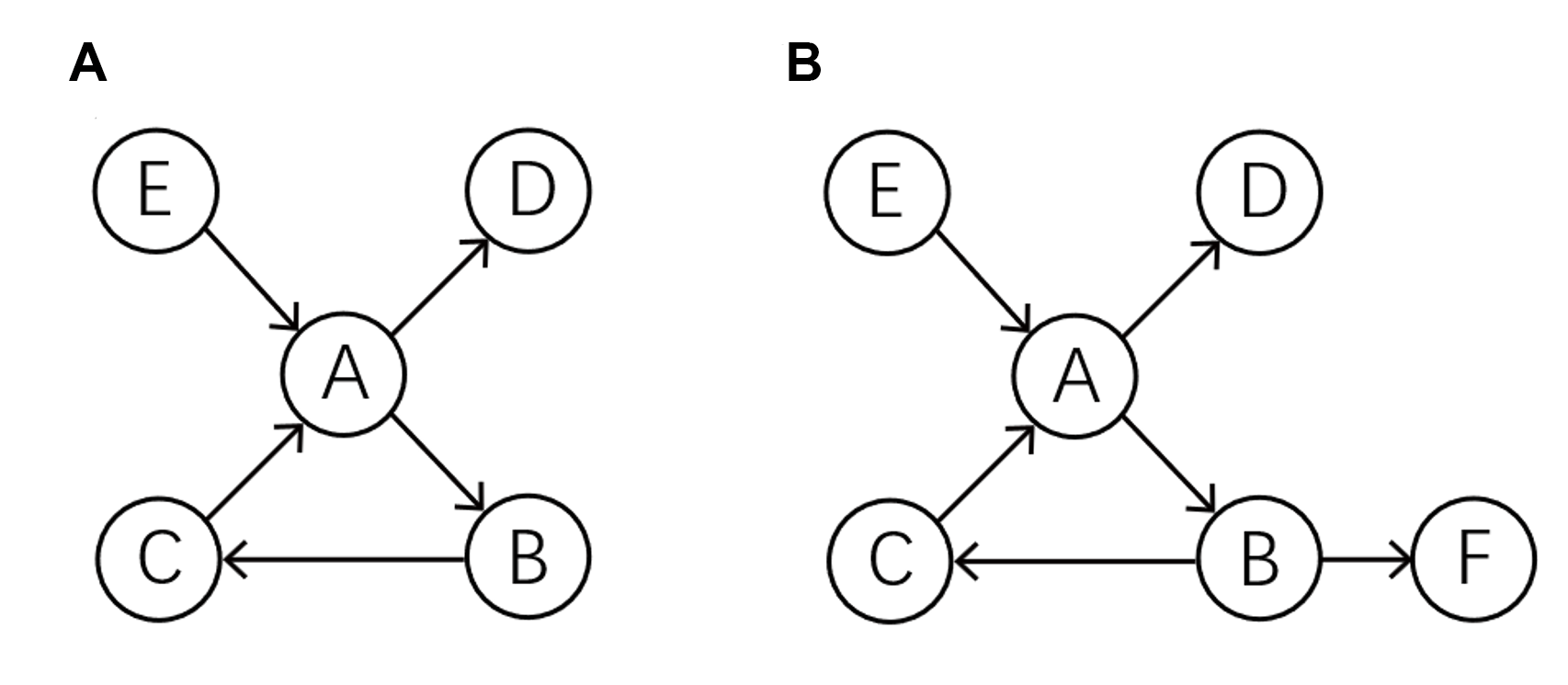


**Fig. S24** An illustration of two examples DFS may encounter during the process of assembling unitigs into contigs; (**A**) observe that even though A is a branching node, it is resolvable by visiting twice A according to the DFS timestamp, thus producing the contig $E\to A\to B\to C\to A\to D$; (**B**) while A is resolvable, B is unresolvable because it cannot be revisited later in the DFS; in this case, three contigs would be generated, namely $E\to A\to B, C\to A\to D,$ and $F$.
