# Supplementary material for: TRFill: synergistic use of HiFi and Hi-C sequencing enables accurate assembly of tandem repeats for population-level analysis
Source: Genome Biol. 2025 Jul 28;26:227. doi: 10.1186/s13059-025-03685-5 (PMC12305924; doi:10.1186/s13059-025-03685-5)
Supplement: Supplementary file 3 — Additional file 3. [file 13059_2025_3685_MOESM3_ESM.pdf]

TRFill: synergistic use of HiFi and Hi-C sequencing enables accurate assembly of tandem repeats for population-level analysis

Authors

Huaming Wen[1,2,†], Jinbao Yang[2,†], Xianjia Zhao[2], Xingbin Wang[2], Jiawei Lei[2], Yanchun Li[4], Wenjie Du[2], Dongxi Li[4], Yun Xu[1], Stefano Lonardi[3,*], Weihua Pan[2,*]

Affiliations

[1]School of Computer Science and Technology, University of Science and Technology of China, Hefei, 230027, China

[2]State Key Laboratory of Genome and Multi-omics Technologies，Shenzhen Branch, Guangdong Laboratory for Lingnan Modern Agriculture, Genome Analysis Laboratory of the Ministry of Agriculture and Rural Affairs, Agricultural Genomics Institute at Shenzhen, Chinese Academy of Agricultural Sciences, Shenzhen 518120, China

[3]Department of Computer Science and Engineering, University of California, Riverside, CA 92521, USA

[4]College of Computer Science and Technology, Taiyuan University of Technology, Taiyuan 030024, China

[†]These authors contributed equally: Huaming Wen, Jinbao Yang

*Corresponding authors

Weihua Pan － Email: panweihua@caas.cn

Stefano Lonardi - Email: stelo@cs.ucr.edu

Table S1

Maternal							
Chromosome	True length	Assembler	Correct length	Assembly length	Completeness	Correctness	LIS score
chr1	4,813,142	hifiasm+3DDNA (original)	783,214	783,285	16.27%	99.99%	1.00
	4,813,142	TRFill	4,766,066	4,767,061	99.02%	99.98%	1.00
	4,813,142	LR_Gapcloser	1,500	1,500	0.03%	100.00%	0.00
	4,813,142	SAMBA	0	0	0.00%	0.00%	0.00
chr2	2,080,300	hifiasm+3DDNA (original)	1,759,085	2,268,935	84.56%	77.53%	0.99
	2,080,300	TRFill	2,080,300	2,098,813	100.00%	99.12%	1.00
	2,080,300	LR_Gapcloser	0	1,302	0.00%	0.00%	0.00
	2,080,300	SAMBA	0	0	0.00%	0.00%	0.00
chr3	4,395,012	hifiasm+3DDNA (original)	448,612	981,280	10.21%	45.72%	1.00
	4,395,012	TRFill	2,286,571	3,450,958	52.03%	66.26%	1.00
	4,395,012	LR_Gapcloser	1,222	1,222	0.03%	100.00%	0.00
	4,395,012	SAMBA	655	755	0.01%	86.75%	0.00
chr4	3,363,140	hifiasm+3DDNA (original)	1,165,957	2,988,348	34.67%	39.02%	0.96
	3,363,140	TRFill	3,316,172	3,351,170	98.60%	98.96%	1.00
	3,363,140	LR_Gapcloser	0	1,338	0.00%	0.00%	0.00
	3,363,140	SAMBA	1,295	1,295	0.04%	100.00%	0.00
chr5	5,037,069	hifiasm+3DDNA (original)	2,106,777	3,349,328	41.83%	62.90%	1.00
	5,037,069	TRFill	1,251,848	3,721,256	24.85%	33.64%	0.99
	5,037,069	LR_Gapcloser	0	1,168	0.00%	0.00%	0.00
	5,037,069	SAMBA	0	0	0.00%	0.00%	0.00
chr6	2,880,583	hifiasm+3DDNA (original)	832,490	851,653	28.90%	97.75%	1.00
	2,880,583	TRFill	728,195	4,007,967	25.28%	18.17%	0.51
	2,880,583	LR_Gapcloser	1,422	1,422	0.05%	100.00%	0.00
	2,880,583	SAMBA	31,989	32,370	1.11%	98.82%	0.00
chr7	6,053,719	hifiasm+3DDNA (original)	2,610,831	6,499,528	43.13%	40.17%	0.85
	6,053,719	TRFill	2,363,108	2,669,863	39.04%	88.51%	1.00
	6,053,719	LR_Gapcloser	1,001	1,001	0.02%	100.00%	0.00
	6,053,719	SAMBA	0	0	0.00%	0.00%	0.00
chr8	2,221,060	hifiasm+3DDNA (original)	1,373,640	1,652,529	61.85%	83.12%	0.99
	2,221,060	TRFill	239,639	239,639	10.79%	100.00%	0.56
	2,221,060	LR_Gapcloser	1,281	1,281	0.06%	100.00%	0.00
	2,221,060	SAMBA	164,075	165,931	7.39%	98.88%	0.00
chr9	3,776,313	hifiasm+3DDNA (original)	433,659	2,480,810	11.48%	17.48%	1.00
	3,776,313	TRFill	1,838,621	2,744,316	48.69%	67.00%	1.00
	3,776,313	LR_Gapcloser	1,038	1,038	0.03%	100.00%	0.00
	3,776,313	SAMBA	27,004	27,012	0.72%	99.97%	0.00
chr10	2,783,251	hifiasm+3DDNA (original)	2,783,249	2,783,326	100.00%	100.00%	1.00
	2,783,251	TRFill	2,783,126	2,783,252	100.00%	100.00%	1.00
	2,783,251	LR_Gapcloser	0	1,232	0.00%	0.00%	0.00
	2,783,251	SAMBA	0	0	0.00%	0.00%	0.00
chr11	3,699,043	hifiasm+3DDNA (original)	655,164	655,164	17.71%	100.00%	1.00
	3,699,043	TRFill	3,670,464	3,670,464	99.23%	100.00%	1.00
	3,699,043	LR_Gapcloser	1,759	1,759	0.05%	100.00%	0.00
	3,699,043	SAMBA	0	0	0.00%	0.00%	0.00
chr12	2,924,269	hifiasm+3DDNA (original)	2,694,194	2,749,363	92.13%	97.99%	1.00
	2,924,269	TRFill	2,908,616	2,909,134	99.46%	99.98%	1.00
	2,924,269	LR_Gapcloser	1,235	1,235	0.04%	100.00%	0.00
	2,924,269	SAMBA	0	0	0.00%	0.00%	0.00
chr16	1,923,048	hifiasm+3DDNA (original)	1,815,597	2,152,563	94.41%	84.35%	1.00
	1,923,048	TRFill	1,923,048	1,938,022	100.00%	99.23%	1.00
	1,923,048	LR_Gapcloser	1,091	1,265	0.06%	86.25%	0.00
	1,923,048	SAMBA	8,150	142,851	0.42%	5.71%	0.00

Table S1

chr17	2,574,968	hifiasm+3DDNA (original)	1,932,958	2,025,882	75.07%	95.41%	1.00
	2,574,968	TRFill	1,740,642	2,079,024	67.60%	83.72%	0.77
	2,574,968	LR_Gapcloser	1,461	1,461	0.06%	100.00%	0.00
	2,574,968	SAMBA	0	325	0.00%	0.00%	0.00
chr18	3,298,595	hifiasm+3DDNA (original)	1,128,643	2,727,742	34.22%	41.38%	0.94
	3,298,595	TRFill	2,269,319	4,871,534	68.80%	46.58%	0.92
	3,298,595	LR_Gapcloser	1,060	1,060	0.03%	100.00%	0.00
	3,298,595	SAMBA	0	51,056	0.00%	0.00%	0.00
chr19	4,698,052	hifiasm+3DDNA (original)	78,908	78,933	1.68%	99.97%	0.98
	4,698,052	TRFill	4,685,747	4,685,964	99.74%	100.00%	1.00
	4,698,052	LR_Gapcloser	0	1,018	0.00%	0.00%	0.00
	4,698,052	SAMBA	0	0	0.00%	0.00%	0.00
chr20	3,064,868	hifiasm+3DDNA (original)	2,835,656	2,896,284	92.52%	97.91%	1.00
	3,064,868	TRFill	3,061,873	3,062,218	99.90%	99.99%	1.00
	3,064,868	LR_Gapcloser	1,337	1,758	0.04%	76.05%	0.00
	3,064,868	SAMBA	0	0	0.00%	0.00%	0.00
chrX	3,088,339	hifiasm+3DDNA (original)	3,087,234	3,092,559	99.96%	99.83%	1.00
	3,088,339	TRFill	3,088,339	3,123,915	100.00%	98.86%	1.00
	3,088,339	LR_Gapcloser	1,233	1,233	0.04%	100.00%	0.00
	3,088,339	SAMBA	1,105	1,497	0.04%	73.81%	0.00

Table S1: Statistics of the assemblies of the centromeric alpha satellite sequences in human HG002 genome from original hifiasm+hic pipeline, and reassembled by TRFill, LR_Gapcloser and SAMBA.
True_lenght: total length of ground truth
Assembly_len: total length of the assembly
Correct_len: total length of the assembly regions appearing in the ground truth
Completeness: Correct_len / Truth_len
Correctness: Correct_len / Assembly_len
Red font identifies assemblies with completeness > 95% with the original hifiasm+hic pipeline
Light blue shows the centromeres of which TRFill successfully improved the assemblies from hifiasm+hic pipeline (completeness and correctness both improved or completeness improved by at least 10% with better or comparable correctness)
Acrocentric chromosomes (Chr13, Chr14, Chr15, Chr21, Chr22) were excluded

Table S1

Paternal							
Chromosome	True length	Assembler	Correct length	Assembly length	Completeness	Correctness	LIS score
chr1	6,713,414	hifiasm+3DDNA (original)	0	0	0.00%	0.00%	0.40
	6,713,414	TRFill	6,708,361	6,738,010	99.92%	99.56%	1.00
	6,713,414	LR_Gapcloser	1,250	1,269	0.02%	98.50%	0.00
	6,713,414	SAMBA	0	0	0.00%	0.00%	0.00
chr2	2,226,870	hifiasm+3DDNA (original)	0	0	0.00%	0.00%	0.94
	2,226,870	TRFill	2,064,985	2,065,086	92.73%	100.00%	0.96
	2,226,870	LR_Gapcloser	1,108	1,111	0.05%	99.73%	0.00
	2,226,870	SAMBA	0	0	0.00%	0.00%	0.00
chr3	3,677,092	hifiasm+3DDNA (original)	533,043	795,665	14.50%	66.99%	0.99
	3,677,092	TRFill	3,435,222	4,864,039	93.42%	70.62%	1.00
	3,677,092	LR_Gapcloser	0	1,000	0.00%	0.00%	0.00
	3,677,092	SAMBA	23,922	180,370	0.65%	13.26%	0.00
chr4	4,296,806	hifiasm+3DDNA (original)	2,439,349	3,130,695	56.77%	77.92%	0.96
	4,296,806	TRFill	4,097,558	4,099,109	95.36%	99.96%	1.00
	4,296,806	LR_Gapcloser	1,122	1,122	0.03%	100.00%	0.00
	4,296,806	SAMBA	0	0	0.00%	0.00%	0.00
chr5	5,193,960	hifiasm+3DDNA (original)	2,692,403	3,792,453	51.84%	70.99%	1.00
	5,193,960	TRFill	203,132	3,799,439	3.91%	5.35%	0.72
	5,193,960	LR_Gapcloser	1,001	1,001	0.02%	100.00%	0.00
	5,193,960	SAMBA	0	0	0.00%	0.00%	0.00
chr6	3,469,334	hifiasm+3DDNA (original)	816,628	816,628	23.54%	100.00%	1.00
	3,469,334	TRFill	1,691,038	3,016,409	48.74%	56.06%	0.99
	3,469,334	LR_Gapcloser	1,285	1,285	0.04%	100.00%	0.00
	3,469,334	SAMBA	22,920	23,221	0.66%	98.70%	0.00
chr7	2,937,372	hifiasm+3DDNA (original)	913,936	1,253,528	31.11%	72.91%	1.00
	2,937,372	TRFill	2,031,773	3,013,357	69.17%	67.43%	0.97
	2,937,372	LR_Gapcloser	1,000	1,132	0.03%	88.34%	0.00
	2,937,372	SAMBA	0	0	0.00%	0.00%	0.00
chr8	2,630,388	hifiasm+3DDNA (original)	1,184,597	1,184,597	45.04%	100.00%	0.74
	2,630,388	TRFill	570,649	1,608,038	21.69%	35.49%	0.99
	2,630,388	LR_Gapcloser	1,095	1,153	0.04%	94.97%	0.00
	2,630,388	SAMBA	14,580	14,580	0.55%	100.00%	0.00
chr9	4,628,848	hifiasm+3DDNA (original)	2,998,762	3,797,390	64.78%	78.97%	1.00
	4,628,848	TRFill	3,629,364	3,629,364	78.41%	100.00%	1.00
	4,628,848	LR_Gapcloser	1,225	1,246	0.03%	98.31%	0.00
	4,628,848	SAMBA	0	0	0.00%	0.00%	0.00
chr10	3,809,359	hifiasm+3DDNA (original)	1,129,485	1,130,082	29.65%	99.95%	1.00
	3,809,359	TRFill	1,129,704	1,880,087	29.66%	60.09%	0.98
	3,809,359	LR_Gapcloser	403	1,058	0.01%	38.09%	0.00
	3,809,359	SAMBA	0	0	0.00%	0.00%	0.00
chr11	2,336,743	hifiasm+3DDNA (original)	1,196,579	1,196,579	51.21%	100.00%	1.00
	2,336,743	TRFill	2,314,530	2,316,155	99.05%	99.93%	1.00
	2,336,743	LR_Gapcloser	1,302	1,302	0.06%	100.00%	0.00
	2,336,743	SAMBA	18,943	18,943	0.81%	100.00%	0.00
chr12	3,118,790	hifiasm+3DDNA (original)	2,668,340	2,668,340	85.56%	100.00%	1.00
	3,118,790	TRFill	3,081,989	3,094,064	98.82%	99.61%	1.00
	3,118,790	LR_Gapcloser	0	1,011	0.00%	0.00%	0.00
	3,118,790	SAMBA	0	0	0.00%	0.00%	0.00
chr16	897,814	hifiasm+3DDNA (original)	704,883	2,154,276	78.51%	32.72%	1.00
	897,814	TRFill	897,814	1,808,058	100.00%	49.66%	1.00
	897,814	LR_Gapcloser	0	0	0.00%	0.00%	0.00
	897,814	SAMBA	0	0	0.00%	0.00%	0.00

Table S1

**chr17**	2,276,421	hifiasm+3DDNA (original)	2,276,421	2,340,387	100.00%	97.27%	1.00
	2,276,421	TRFill	1,872,076	4,167,437	82.24%	44.92%	0.90
	2,276,421	LR_Gapcloser	1,588	1,588	0.07%	100.00%	0.00
	2,276,421	SAMBA	0	0	0.00%	0.00%	0.00
**chr18**	3,670,926	hifiasm+3DDNA (original)	1,965,946	1,965,946	53.55%	100.00%	0.94
	3,670,926	TRFill	3,142,030	3,178,674	85.59%	98.85%	0.93
	3,670,926	LR_Gapcloser	1,239	1,239	0.03%	100.00%	0.00
	3,670,926	SAMBA	11,234	11,234	0.31%	100.00%	0.00
**chr19**	4,727,959	hifiasm+3DDNA (original)	451,096	696,181	9.54%	64.80%	0.91
	4,727,959	TRFill	4,652,211	4,652,838	98.40%	99.99%	1.00
	4,727,959	LR_Gapcloser	0	1,295	0.00%	0.00%	0.00
	4,727,959	SAMBA	0	0	0.00%	0.00%	0.00
**chr20**	3,291,853	hifiasm+3DDNA (original)	1,647,873	1,647,896	50.06%	100.00%	1.00
	3,291,853	TRFill	2,799,520	2,800,184	85.04%	99.98%	1.00
	3,291,853	LR_Gapcloser	1,455	1,663	0.04%	87.49%	0.00
	3,291,853	SAMBA	0	0	0.00%	0.00%	0.00
**chrY**	317,536	hifiasm+3DDNA (original)	0	73,967	0.00%	0.00%	1.00
	317,536	TRFill	317,536	326,075	100.00%	97.38%	1.00
	317,536	LR_Gapcloser	1,554	1,554	0.49%	100.00%	0.00
	317,536	SAMBA	0	0	0.00%	0.00%	0.00

Table S1: Statistics of the assemblies of the centromeric alpha satellite sequences in human HG002 genome from original hifiasm+hic pipeline, and reassembled by TRFill, LR_Gapcloser and SAMBA.
True_lenght: total length of ground truth
Assembly_len: total length of the assembly
Correct_len: total length of the assembly regions appearing in the ground truth
Completeness: Correct_len / Truth_len
Correctness: Correct_len / Assembly_len
Red font identifies assemblies with completeness > 95% with the original hifiasm+hic pipeline
Light blue shows the centromeres of which TRFill successfully improved the assemblies from hifiasm+hic pipeline (completeness and correctness both improved or completeness improved by at least 10% with better or comparable correctness)
Acrocentric chromosomes (Chr13, Chr14, Chr15, Chr21, Chr22) were excluded

Table S2

Example	Peak memory	Peak Storage	CPU time (s)	Wall clock time (h:m:s)	Gap length (bp)	Type	Note
HG002	31.2 G	30 G	1,340,774	10:01:14	6 M	Diploid	Chr1 centromere
TS2	25.84 G	4.4 G	301675	2:21:51	1.1 M	Haploid	Chr1 subtelomere
TS281	25.85 G	4.4 G	110788	1:08:25	110 k	Haploid	Chr1 subtelomere

Table S2: Resource consumption of TRFill. The experiments were conducted on a server running Ubuntu 20.04 (Linux kernel 5.15.0) with dual EPYC 7H12 CPUs (256 threads, x86_64, 3252 MHz) and 1 TB of memory.

Table S3

Maternal						
**HiFi depth**	**Hi-C depth**	**Correct length**	**True length**	**Assembly length**	**Completeness**	**Correctness**
36	69	4,710,499	4,731,260	4,715,602	99.56%	99.89%
36	34	4,710,499	4,731,260	4,715,609	99.56%	99.89%
36	17	4,710,499	4,731,260	4,715,609	99.56%	99.89%
18	17	2,532,537	4,731,260	3,565,228	53.53%	71.03%
18	8	2,549,942	4,731,260	3,198,767	53.90%	79.72%
9	8	57,396	4,731,260	57,403	1.21%	99.99%

Paternal						
**HiFi depth**	**Hi-C depth**	**Correct length**	**True length**	**Assembly length**	**Completeness**	**Correctness**
36	69	4,675,322	4,694,286	4,676,330	99.60%	99.98%
36	34	4,672,122	4,694,286	4,672,629	99.53%	99.99%
36	17	4,672,122	4,694,286	4,672,629	99.53%	99.99%
18	17	242,123	4,694,286	1,768,896	5.16%	13.69%
18	8	242,123	4,694,286	1,768,896	5.16%	13.69%
9	8	360,774	4,694,286	591,287	7.69%	61.02%

Table S3: Statistics of the TRFill assemblies of the centromeric alpha satellite sequences in Chr19 of human HG002 genome with different HiFi and Hi-C sequencing depths.
True length: Total length of the ground truth assembly
Assembly length: Total length of the assembly
Correct length: Total length of the assembly regions appearing in the ground truth
Completeness: Correct length / Truth length
Correctness: Correct length / Assembly length
HiFi depth: Sequencing depth of HiFi reads
Hi-C depth: Sequencing depth of Hi-C reads

Table S4

Subtelomere	Start position	End position	Length
chr01_1	0	360,000	360,000
chr03_1	20,000	490,000	470,000
chr03_2	67,380,000	68,200,000	820,000
chr04_1	40,000	740,000	700,000
chr04_2	67,970,000	68,300,000	330,000
chr05_1	0	730,000	730,000
chr05_2	66,370,000	66,690,000	320,000
chr06_1	0	140,000	140,000
chr07_1	10,000	700,000	690,000
chr07_2	68,940,000	69,340,000	400,000
chr08_1	0	150,000	150,000
chr08_2	67,580,000	68,080,000	500,000
chr09_1	0	470,000	470,000
chr09_2	69,580,000	70,290,000	710,000
chr10_1	70,000	500,000	430,000
chr10_2	66,510,000	66,910,000	400,000
chr11_1	20,000	360,000	340,000
chr12_1	10,000	430,000	420,000
chr12_2	68,320,000	68,600,000	280,000

Table S4: The 19 subtelomeric tandem repeats in the reference tomato genome (Heinz1706)
Start position: starting position on the reference genome
End position: ending position on the reference genome
Length: length of the subtelomeric tandem repeat
Subtelomere: chrX_Y where X is the chromosome number and Y indicates the subtelomeres number

Table S5

TS2							
**Subtelomere**	**True length**	**Correct length**	**Assembly length**	**Completeness**	**Correctness**	**F1**	**LIS score**
chr01_1	1,035,997	488,332	488,332	47.14%	100.00%	0.64	1.00
chr03_1	470,000	469,938	470,000	99.99%	99.99%	1.00	1.00
chr03_2	897,991	42,298	147,078	4.71%	28.76%	0.08	0.27
chr04_1	713,368	712,437	712,437	99.87%	100.00%	1.00	1.00
chr04_2	364,223	252,999	252,999	69.46%	100.00%	0.82	1.00
chr05_1	705,405	62,768	107,160	8.90%	58.57%	0.15	0.64
chr05_2	320,000	319,982	320,000	99.99%	99.99%	1.00	1.00
chr06_1	140,000	140,000	140,000	100.00%	100.00%	1.00	1.00
chr07_1	684,993	636,649	690,000	92.94%	92.27%	0.93	1.00
chr07_2	360,630	360,630	368,608	100.00%	97.84%	0.99	1.00
chr08_1	140,073	61,552	61,552	43.94%	100.00%	0.61	1.00
chr08_2	420,766	409,527	417,249	97.33%	98.15%	0.98	1.00
chr09_1	1,454,538	781,898	781,898	53.76%	100.00%	0.70	1.00
chr09_2	714,121	714,121	714,121	100.00%	100.00%	1.00	1.00
chr10_1	463,783	463,783	463,784	100.00%	100.00%	1.00	1.00
chr10_2	441,612	441,610	441,610	100.00%	100.00%	1.00	1.00
chr11_1	354,433	340,000	340,000	95.93%	100.00%	0.98	1.00
chr12_1	420,000	420,000	507,060	100.00%	82.83%	0.91	1.00
chr12_2	795,943	280,000	280,000	35.18%	100.00%	0.52	1.00

Table S5: A (haploid). Statistics of the assemblies of the subtelomeric tandem repeats in haploid tomato genomes from original hifiasm+hic pipeline

Subtelomere: chrX_Y, where X represents the of chromosome number and Y (1 or 2)
True length: total length of the ground truth assembly
Assembly length: total length of the assembly
Correct length: total length of the assembly regions appearing in the ground truth
Completeness: Correct length / Truth length
Correctness: Correct length / Assembly length
Red indicates completeness < 98% or correctness < 98% (low-quality), green indicates indicates completeness >= 98% and correctness >= 98% (high quality)
F1 score: F1 of Completeness and Correctness

Table S5

TS281							
Subtelomere	True length	Correct length	Assembly length	Completeness	Correctness	F1	LIS score
chr01_1	173,611	173,611	173,611	100.00%	100.00%	1.00	0.96
chr03_1	469,224	364,789	390,089	77.74%	93.51%	0.85	1.00
chr03_2	781,845	781,845	781,845	100.00%	100.00%	1.00	0.87
chr04_1	697,563	697,525	697,525	99.99%	100.00%	1.00	1.00
chr04_2	414,394	414,394	414,394	100.00%	100.00%	1.00	0.97
chr05_1	720,959	720,959	720,959	100.00%	100.00%	1.00	0.93
chr05_2	346,461	346,461	346,471	100.00%	100.00%	1.00	1.00
chr06_1	140,000	140,000	140,000	100.00%	100.00%	1.00	1.00
chr07_1	654,002	242,500	242,500	37.08%	100.00%	0.54	1.00
chr07_2	400,000	400,000	400,000	100.00%	100.00%	1.00	0.98
chr08_1	140,896	140,896	140,896	100.00%	100.00%	1.00	1.00
chr08_2	551,444	534,446	534,446	96.92%	100.00%	0.98	0.79
chr09_1	1,049,074	1,049,074	1,049,074	100.00%	100.00%	1.00	0.99
chr09_2	571,139	565,637	566,643	99.04%	99.82%	0.99	0.80
chr10_1	498,389	498,389	498,389	100.00%	100.00%	1.00	1.00
chr10_2	429,126	429,126	429,126	100.00%	100.00%	1.00	1.00
chr11_1	678,950	674,965	674,965	99.41%	100.00%	1.00	0.23
chr12_1	565,937	564,161	564,161	99.69%	100.00%	1.00	0.99
chr12_2	422,229	422,229	422,229	100.00%	100.00%	1.00	0.86

Table S5: A (haploid). Statistics of the assemblies of the subtelomeric tandem repeats in haploid tomato genomes from original hifiasm+hic pipeline

Subtelomere: chrX_Y, where X represents the of chromosome number and Y (1 or 2)
True length: total length of the ground truth assembly
Assembly length: total length of the assembly
Correct length: total length of the assembly regions appearing in the ground truth
Completeness: Correct length / Truth length
Correctness: Correct length / Assembly length
Red indicates completeness < 98% or correctness < 98% (low-quality), green indicates indicates completeness >= 98% and correctness >= 98% (high quality)
F1 score: F1 of Completeness and Correctness

Table S5

TS2							
**Subtelomere**	**True length**	**Correct length**	**Assembly length**	**Completeness**	**Correctness**	**F1**	**LIS score**
chr01_1	1,035,997	78,306	173,611	7.56%	45.10%	0.13	0.96
chr03_1	470,000	469,989	470,000	100.00%	100.00%	**1.00**	1.00
chr03_2	897,991	712,591	712,591	79.35%	100.00%	**0.88**	1.00
chr04_1	713,368	713,369	713,605	100.00%	99.97%	**1.00**	1.00
chr04_3	364,223	254,549	254,549	69.89%	100.00%	**0.82**	1.00
chr05_1	705,405	128,674	712,256	18.24%	18.07%	0.18	0.93
chr05_2	320,000	0	0	0.00%	0.00%	0.00	0.00
chr06_1	140,000	0	0	0.00%	0.00%	0.00	0.00
chr07_1	684,993	364,457	364,457	53.21%	100.00%	**0.69**	1.00
chr07_4	360,630	360,629	368,607	100.00%	97.84%	0.99	1.00
chr08_1	140,073	0	0	0.00%	0.00%	0.00	0.00
chr08_3	420,766	76,317	551,444	18.14%	13.84%	**0.16**	0.83
chr09_1	1,454,538	470,000	470,000	32.31%	100.00%	**0.49**	1.00
chr09_5	714,121	183,304	565,228	25.67%	32.43%	0.29	0.80
chr10_1	463,783	9,531	498,389	2.06%	1.91%	0.02	1.00
chr10_2	441,612	0	0	0.00%	0.00%	0.00	1.00
chr11_1	354,433	24,223	588,406	6.83%	4.12%	0.05	0.24
chr12_1	420,000	420,000	507,348	100.00%	82.78%	**0.91**	1.00
chr12_2	795,943	5,274	5,274	0.66%	100.00%	**0.01**	1.00

Table S5: B (diploid). Statistics of the assemblies of the subtelomeric tandem repeats in diploid tomato genomes from original hifiasm+hic pipeline

Subtelomere: chrX_Y, where X represents the of chromosome number and Y (1 or 2) represents the ID of the two subtelomeres in each chromosome

True length: total length of the ground truth assembly
Assembly length: total length of the assembly
Correct length: total length of the assembly regions appearing in the ground truth
Completeness: Correct length / Truth length
Correctness: Correct length / Assembly length
Red indicates completeness < 98% or correctness < 98% (low-quality), green indicates indicates completeness >= 98% and correctness >= 98% (high quality)
F1 score: F1 of Completeness and Correctness

Table S5

TS281							
**Subtelomere**	**True length**	**Correct length**	**Assembly length**	**Completeness**	**Correctness**	**F1**	**LIS score**
chr01_1	173,611	0	0	0.00%	0.00%	0.00	0.00
chr03_1	469,224	449,542	449,542	95.81%	100.00%	**0.98**	1.00
chr03_2	781,845	781,845	781,845	100.00%	100.00%	**1.00**	1.00
chr04_1	697,563	697,491	697,497	99.99%	100.00%	**1.00**	1.00
chr04_3	414,394	414,394	414,394	100.00%	100.00%	**1.00**	1.00
chr05_1	720,959	114,273	815,283	15.85%	14.02%	0.15	0.93
chr05_2	346,461	316,953	320,000	91.48%	99.05%	0.95	1.00
chr06_1	140,000	140,000	140,000	100.00%	100.00%	**1.00**	1.00
chr07_1	654,002	629,951	654,002	96.32%	96.32%	**0.96**	1.00
chr07_4	400,000	400,000	400,000	100.00%	100.00%	**1.00**	1.00
chr08_1	140,896	140,896	140,896	100.00%	100.00%	**1.00**	1.00
chr08_3	551,444	104,419	420,695	18.94%	24.82%	0.21	0.83
chr09_1	1,049,074	1,049,074	1,049,074	100.00%	100.00%	**1.00**	1.00
chr09_5	571,139	157,920	714,121	27.65%	22.11%	0.25	0.80
chr10_1	498,389	35,735	430,000	7.17%	8.31%	0.08	1.00
chr10_2	429,126	429126	441,429	100.00%	97.21%	**0.99**	1.00
chr11_1	678,950	51,023	340,000	7.51%	15.01%	0.10	0.24
chr12_1	565,937	565,937	573,575	100.00%	98.67%	**0.99**	1.00
chr12_2	422,229	50,975	280,000	12.07%	18.21%	0.15	0.92

Table S5: B (diploid). Statistics of the assemblies of the subtelomeric tandem repeats in diploid tomato genomes from original hifiasm+hic pipeline

Subtelomere: chrX_Y, where X represents the of chromosome number and Y (1 or 2) represents the ID of the two subtelomeres in each chromosome

True length: total length of the ground truth assembly
Assembly length: total length of the assembly
Correct length: total length of the assembly regions appearing in the ground truth
Completeness: Correct length / Truth length
Correctness: Correct length / Assembly length
Red indicates completeness < 98% or correctness < 98% (low-quality), green indicates indicates completeness >= 98% and correctness >= 98% (high quality)
F1 score: F1 of Completeness and Correctness

Table S6

Subtelomere	True length		Correct length	Assembly length	Completeness	Correctness
**TS2**						
chr01_1	1,035,997	Original	488,332	488,332	47.14%	100.00%
	1,035,997	TRFill	852,330	852,349	82.27%	100.00%
chr03_2	897,991	Original	42,298	147,078	4.71%	28.76%
	897,991	TRFill	889,247	937,890	99.03%	94.81%
chr04_2	364,223	Original	252,999	252,999	69.46%	100.00%
	364,223	TRFill	254,545	384,716	69.89%	66.16%
chr05_1	705,405	Original	62,768	107,160	8.90%	58.57%
	705,405	TRFill	631,765	631,765	89.56%	100.00%
chr07_1	684,993	Original	332,175	690,000	48.49%	48.14%
	684,993	TRFill	364,457	696,844	53.21%	52.30%
chr07_2	360,630	Original	360,630	368,608	100.00%	97.84%
	360,630	TRFill	360,632	361,328	100.00%	99.81%
chr08_1	140,073	Original	61,552	61,552	43.94%	100.00%
	140,073	TRFill	140,073	140,362	100.00%	99.79%
chr08_2	420,766	Original	409,527	417,249	97.33%	98.15%
	420,766	TRFill	409,621	728,537	97.35%	56.23%
chr09_1	1,454,538	Original	781,898	781,898	53.76%	100.00%
	1,454,538	TRFill	152,317	477,846	10.47%	31.88%
chr11_1	354,433	Original	340,000	340,000	95.93%	100.00%
	354,433	TRFill	354,432	354,522	100.00%	99.97%
chr12_1	420,000	Original	420,000	507,060	100.00%	82.83%
	420,000	TRFill	419,999	420,046	100.00%	99.99%
chr12_2	795,943	Original	280,000	280,000	35.18%	100.00%
	795,943	TRFill	304,171	304,235	38.22%	99.98%
**TS281**						
**Subtelomere**	**True_length**		**Correct length**	**Assembly length**	**Completeness**	**Correctness**
chr03_1	469,224	Original	364,789	390,089	77.74%	93.51%
	469,224	TRFill	469,247	469,993	100.00%	99.84%
chr07_1	654,002	Original	242,500	242,500	37.08%	100.00%
	654,002	TRFill	383,956	745,932	58.71%	51.47%
chr08_2	551,444	Original	534,446	534,446	96.92%	100.00%
	551,444	TRFill	551,444	552,039	100.00%	99.89%

Table S6: A (haploid). Statistics of the assemblies of the subtelomeric tandem repeats in haploid tomato genomes from original hifiasm+hic pipeline and TRFill

Subtelomere: chrX_Y, where X represents the of chromosome number and Y (1 or 2) represents the ID of the two subtelomeres in each chromosome

True length: total length of the ground truth assembly

Assembly length: total length of the assembly

Correct length: total length of the assembly regions appearing in the ground truth

Completeness: Correct length / Truth length

Correctness: Correct length / Assembly length

Green color shows the subtelomeric repeats that TRFill successfully improved

Table S6

TS2							
**Subtelomere**	**True length**		**Correct length**	**Assembly length**	**Completeness**	**Correctness**	**F1**
chr01_1	1,035,997	Original	78,306	173,611	7.56%	45.10%	0.13
	1,035,997	TRFill	87,570	181,676	8.45%	48.20%	0.14
chr03_2	897,991	Original	712,591	712,591	79.35%	100.00%	0.88
	897,991	TRFill	168,493	759,529	18.76%	22.18%	0.20
chr04_2	364,223	Original	254,549	254,549	69.89%	100.00%	0.82
	364,223	TRFill	254,544	384,212	69.89%	66.25%	0.68
chr05_1	705,405	Original	128,674	712,256	18.24%	18.07%	0.18
	705,405	TRFill	576,255	576,255	81.69%	100.00%	0.90
chr05_2	320,000	Original	0	0	0.00%	0.00%	0.00
	320,000	TRFill	320,000	320,175	100.00%	99.95%	1.00
chr06_1	140,000	Original	0	0	0.00%	0.00%	0.00
	140,000	TRFill	59,901	60,009	42.79%	99.82%	0.60
chr07_1	684,993	Original	364,457	364,457	53.21%	100.00%	0.69
	684,993	TRFill	443,793	819,230	64.79%	54.17%	0.59
chr07_2	360,630	Original	360,629	368,607	100.00%	97.84%	0.99
	360,630	TRFill	360,631	361,327	100.00%	99.81%	1.00
chr08_1	140,073	Original	0	0	0.00%	0.00%	0.00
	140,073	TRFill	125,166	125,174	89.36%	99.99%	0.94
chr08_2	420,766	Original	76,317	551,444	18.14%	13.84%	0.16
	420,766	TRFill	43,670	170,740	10.38%	25.58%	0.15
chr09_1	1,454,538	Original	470,000	470,000	32.31%	100.00%	0.49
	1,454,538	TRFill	20,809	303,309	1.43%	6.86%	0.02
chr09_2	714,121	Original	183,304	565,228	25.67%	32.43%	0.29
	714,121	TRFill	161,426	245,053	22.60%	65.87%	0.34
chr10_1	463,783	Original	9,531	498,389	2.06%	1.91%	0.02
	463,783	TRFill	16,236	538,315	3.50%	3.02%	0.03
chr10_2	441,612	Original	0	0	0.00%	0.00%	0.00
	441,612	TRFill	331,836	380,327	75.14%	87.25%	0.81
chr11_1	354,433	Original	24,223	588,406	6.83%	4.12%	0.05
	354,433	TRFill	354,432	354,522	100.00%	99.97%	1.00
chr12_1	420,000	Original	420,000	507,348	100.00%	82.78%	0.91
	420,000	TRFill	240,943	541,844	57.37%	44.47%	0.50
chr12_2	795,943	Original	5,274	5,274	0.66%	100.00%	0.01
	795,943	TRFill	304,173	329,043	38.22%	92.44%	0.54

Table S6: B (diploid). Statistics of the assemblies of the subtelomeric tandem repeats in the synthetic diploid tomato genome (TS2 and TS281 as two haploytpes) from original hifiasm+hic pipeline and TRFill

Subtelomere: chrX_Y, where X represents the of chromosome number and Y (1 or 2) represents the ID of the two subtelomeres in each chromosome

True length: total length of the ground truth assembly

Assembly length: total length of the assembly

Correct length: total length of the assembly regions appearing in the ground truth

Completeness: Correct length / Truth length

Correctness: Correct length / Assembly length

F1 score: F1 of Completeness and Correctness

Green color shows the subtelomeric repeats that TRFill successfully improved

Table S6

TS281							
Subtelomere	True length		Correct length	Assembly length	Completeness	Correctness	F1
chr01_1	173,611	Original	0	0	0.00%	0.00%	0.00
	173,611	TRFill	99,150	522,225	57.11%	18.99%	0.19
chr03_1	469,224	Original	449,542	449,542	95.81%	100.00%	1.00
	469,224	TRFill	459,148	477,575	97.85%	96.14%	0.96
chr05_1	720,959	Original	114,273	815,283	15.85%	14.02%	0.14
	720,959	TRFill	408,319	408,365	56.64%	99.99%	1.00
chr05_2	346,461	Original	316,953	320,000	91.48%	99.05%	0.99
	346,461	TRFill	346,461	346,532	100.00%	99.98%	1.00
chr07_1	654,002	Original	629,951	654,002	96.32%	96.32%	0.96
	654,002	TRFill	152,529	313,611	23.32%	48.64%	0.49
chr08_2	551,444	Original	104,419	420,695	18.94%	24.82%	0.25
	551,444	TRFill	551,444	552,039	100.00%	99.89%	1.00
chr09_2	571,139	Original	157,920	714,121	27.65%	22.11%	0.22
	571,139	TRFill	566,173	732,318	99.13%	77.31%	0.77
chr10_1	498,389	Original	35,735	430,000	7.17%	8.31%	0.08
	498,389	TRFill	59,926	425,016	12.02%	14.10%	0.14
chr10_2	429,126	Original	429,126	441,429	100.00%	97.21%	0.97
	429,126	TRFill	423,256	423,835	98.63%	99.86%	1.00
chr11_1	678,950	Original	51,023	340,000	7.51%	15.01%	0.15
	678,950	TRFill	70,284	370,488	10.35%	18.97%	0.19
chr12_2	422,229	Original	50,975	280,000	12.07%	18.21%	0.18
	422,229	TRFill	422,229	422,229	100.00%	100.00%	1.00

Table S6: B (diploid). Statistics of the assemblies of the subtelomeric tandem repeats in the synthetic diploid tomato genome (TS2 and TS281 as two haploytpes) from original hifiasm+hic pipeline and TRFill

Subtelomere: chrX_Y, where X represents the of chromosome number and Y (1 or 2) represents the ID of the two subtelomeres in each chromosome

True length: total length of the ground truth assembly

Assembly length: total length of the assembly

Correct length: total length of the assembly regions appearing in the ground truth

Completeness: Correct length / Truth length

Correctness: Correct length / Assembly length

F1 score: F1 of Completeness and Correctness

Green color shows the subtelomeric repeats that TRFill successfully improved

Table S7

Accession	Name	Species
TS9	Ailsa Craig	*Solanum lycopersicum*
TS80	-	*Solanum lycopersicum*
TS692	Wheatleys Frost Resistant	*Solanum lycopersicum*
TS629	Yellow Pear	*Solanum lycopersicum*
TS60	New Yorker	*Solanum lycopersicum*
TS331	-	*Solanum lycopersicum*
TS3	M-82	*Solanum lycopersicum*
TS204	Florida 7060	*Solanum lycopersicum*
TS2	Moneymaker	*Solanum lycopersicum*
TS185	B-L-35	*Solanum lycopersicum*
TS166	Piura	*Solanum lycopersicum*
TS12	yoku improvement	*Solanum lycopersicum*
TS117	Scatolone di bolsena	*Solanum lycopersicum*
TS112	-	*Solanum lycopersicum*
SL5.0	Heinz 1706	*Solanum lycopersicum*
TS96	-	*Solanum lycopersicum* var. *cerasiforme*
TS623	Sugar Lump	*Solanum lycopersicum* var. *cerasiforme*
TS545	Peacevine Cherry	*Solanum lycopersicum* var. *cerasiforme*
TS39	Cerise Gold	*Solanum lycopersicum* var. *cerasiforme*
TS281	San Martin de Pangoa	*Solanum lycopersicum* var. *cerasiforme*
TS280	Cerise du sud ouest N 2	*Solanum lycopersicum* var. *cerasiforme*
TS238	Macas	*Solanum lycopersicum* var. *cerasiforme*
TS118	Santa Cruz, Galapagos	*Solanum lycopersicum* var. *cerasiforme*
TS439	-	*Solanum pimpinellifolium*
TS421	-	*Solanum pimpinellifolium*
TS413	-	*Solanum pimpinellifolium*
TS265	Hacienda Buenos Aires	*Solanum pimpinellifolium*
TS222	Wva 700	*Solanum pimpinellifolium*
TS22	Pisiquillo	*Solanum pimpinellifolium*
TS156	Balsa Huaico	*Solanum pimpinellifolium*

Table S7: The 29 tomato genomes included in population-level analysis

Table S8

Accession	Subtelomere	Ref length	TRFill length	Original length	Common	Common / TRFill	Common / Original	Partition
M82	1_1	360,000	391,550	103,171	103,139	26.34%	99.97%	1
	3_1	470,000	498,487	470000	470,000	94.29%	100.00%	1
	3_2	820,000	785,992	94,372	94,172	11.98%	99.79%	1
	4_1	700,000	747,610	366,356	366,311	49.00%	99.99%	1
	4_3	330,000	283,651	77,640	72,949	25.72%	93.96%	1
	5_1	730,000	711,913	96,244	93,344	13.11%	96.99%	1
	5_2	320,000	364,552	176,038	175,684	48.19%	99.80%	1
	6_1	140,000	211,294	140000	139,914	66.22%	99.94%	1
	7_1	690,000	832,286	373,308	373,272	44.85%	99.99%	1
	7_4	400,000	385,497	85,611	84,790	21.99%	99.04%	1
	8_1	150,000	263,408	150000	142,127	53.96%	94.75%	1
	8_3	500,000	576,195	106,987	106,961	18.56%	99.98%	1
	9_1	470,000	988,304	207,404	207,392	20.98%	99.99%	1
	9_5	710,000	808,826	96,625	93,402	11.55%	96.66%	1
	10_1	430,000	510,142	248,079	248,028	48.62%	99.98%	1
	10_2	400,000	433,342	196,729	196,729	45.40%	100.00%	1
	11_1	340,000	411,867	208,001	193,856	47.07%	93.20%	1
	12_1	420,000	452,988	172,428	167,859	37.06%	97.35%	1
	12_2	280,000	225,490	269,652	92,574	41.05%	34.33%	1
Moneymaker	1_1	360,000	876,716	488,332	488,306	55.70%	99.99%	1
	3_1	470,000	493660	470000	469,969	95.20%	99.99%	1
	3_2	820,000	1,042,260	147,078	85,959	8.25%	58.44%	1
	4_1	700,000	743,905	712,437	707,845	95.15%	99.36%	1
	4_3	330,000	400,812	252,999	252,957	63.11%	99.98%	1
	5_1	730,000	868,762	107,160	107,054	12.32%	99.90%	1
	5_2	320,000	411,733	320000	317,995	77.23%	99.37%	1
	6_1	140,000	242,217	140000	139,035	57.40%	99.31%	1
	7_1	690,000	766,807	690000	689,922	89.97%	99.99%	1
	7_4	400,000	393,906	368,608	368,574	93.57%	99.99%	1
	8_1	150,000	263,511	61,552	61,512	23.34%	99.94%	1
	8_3	500,000	748,138	417,249	411,118	54.95%	98.53%	1
	9_1	470,000	492,223	781,898	317,541	64.51%	40.61%	1
	9_5	710,000	831,735	714,121	704,070	84.65%	98.59%	1
	10_1	430,000	534,723	463,784	463,769	86.73%	100.00%	1
	10_2	400,000	523,039	441,610	441,597	84.43%	100.00%	1
	11_1	340,000	388887	340000	339,938	87.41%	99.98%	1
	12_1	420,000	527,440	507,060	507,060	96.14%	100.00%	1
	12_2	280,000	320,895	280000	279,257	87.02%	99.73%	1
TS9	1_1	360,000	556,926	324,838	324,812	58.32%	99.99%	1
	3_1	470,000	502,512	470000	464,478	92.43%	98.83%	1
	3_2	820,000	999,177	897,308	897,308	89.80%	100.00%	1
	4_1	700,000	721,203	703,963	702,975	97.47%	99.86%	1
	4_3	330,000	393,699	210,193	210,118	53.37%	99.96%	1
	5_1	730,000	865,432	213,261	212,797	24.59%	99.78%	1
	5_2	320,000	419775	320000	313,521	74.69%	97.98%	1
	6_1	140,000	201,688	140000	139,576	69.20%	99.70%	1
	7_1	690,000	757,171	756,278	739,796	97.71%	97.82%	1
	7_4	400,000	671,662	332,559	331,083	49.29%	99.56%	1
	8_1	150,000	311208	150000	149,925	48.18%	99.95%	1
	8_3	500,000	380,788	388,586	330,865	86.89%	85.15%	1
	9_1	470,000	480,629	982,987	329,850	68.63%	33.56%	1
	9_5	710,000	573,015	568,351	562,782	98.21%	99.02%	1
	10_1	430,000	538,805	463,808	463,762	86.07%	99.99%	1
	10_2	400,000	480,194	423,460	423,406	88.17%	99.99%	1
	11_1	340,000	387,613	203,442	189,610	48.92%	93.20%	1
	12_1	420,000	589,138	557,955	557,838	94.69%	99.98%	1
	12_2	280,000	292,149	501,691	165,952	56.80%	33.08%	1
TS12	1_1	360,000	896,461	225,457	225,420	25.15%	99.98%	1
	3_1	470,000	577,108	470000	464,357	80.46%	98.80%	1
	3_2	820,000	1,048,792	965,493	965,432	92.05%	99.99%	1
	4_1	700,000	736,923	711,735	711,696	96.58%	99.99%	1
	4_3	330,000	476,095	336,658	336,745	70.73%	100.03%	1
	5_1	730,000	928,094	-	-	0.00%	#DIV/0!	1
	5_2	320,000	398,658	320000	310,653	77.92%	97.08%	1
	6_1	140,000	256,799	140000	139,348	54.26%	99.53%	1
	7_1	690,000	753,018	738,894	732,135	97.23%	99.09%	1
	7_4	400,000	469,873	400000	399,005	84.92%	99.75%	1
	8_1	150,000	270,410	413,475	250,342	92.58%	60.55%	1
	8_3	500,000	615,783	70,233	70,173	11.40%	99.91%	1
	9_1	470,000	544,274	259,733	247,438	45.46%	95.27%	1
	9_5	710,000	812,843	742,875	561,073	69.03%	75.53%	1
	10_1	430,000	527,205	485,442	485,442	92.08%	100.00%	1
	10_2	400,000	506,341	416,948	414,526	81.87%	99.42%	1
	11_1	340,000	795,723	340000	334,681	42.06%	98.44%	1
	12_1	420,000	706,017	558,858	558,799	79.15%	99.99%	1
	12_2	280,000	561,470	498,164	498,164	88.72%	100.00%	1
TS22	1_1	360,000	136,894	197,304	114,802	83.86%	58.19%	1
	3_1	470,000	218,952	351,258	186,674	85.26%	53.14%	1
	3_2	820,000	976,167	243,680	193,904	19.86%	79.57%	1
	4_1	700,000	662,136	281,835	191,011	28.85%	67.77%	1
	4_3	330,000	343,639	148,264	147,483	42.92%	99.47%	1
	5_1	730,000	371,299	205,006	70,720	19.05%	34.50%	1
	5_2	320,000	389,008	104,115	103,575	26.63%	99.48%	1

Table S8

	6_1	140,000	146,696	232,925	11,023	7.51%	4.73%	1
	7_1	690,000	421,121	355,714	184,616	43.84%	51.90%	1
	7_4	400,000	117,047	81,155	53,461	45.67%	65.88%	1
	8_1	150,000	201,459	51,543	51,543	25.58%	100.00%	1
	8_3	500,000	434,908	75,667	75,667	17.40%	100.00%	1
	9_1	470,000	486,205	265,326	249,054	51.22%	93.87%	1
	9_5	710,000	1,047,469	33,558	33,209	3.17%	98.96%	1
	10_1	430,000	285,723	233,690	219,587	76.85%	93.97%	1
	10_2	400,000	615,891	122,609	122,609	19.91%	100.00%	1
	11_1	340,000	277,270	122,656	114,210	41.19%	93.11%	1
	12_1	420,000	386,439	235,370	234,742	60.74%	99.73%	1
	12_2	280,000	240,451	29,501	29,054	12.08%	98.48%	1
TS39	1_1	360,000	1,335,318	483,997	483,672	36.22%	99.93%	1
	3_1	470,000	193,462	901,078	165,595	85.60%	18.38%	2
	3_2	820,000	829,147	239,227	234,288	28.26%	97.94%	1
	4_1	700,000	872,166	499,522	499,483	57.27%	99.99%	1
	4_3	330,000	66,920	330,000	55,496	82.93%	16.82%	2
	5_1	730,000	845,955	698,289	696,501	82.33%	99.74%	1
	5_2	320,000	426,406	320,000	318,642	74.73%	99.58%	1
	6_1	140,000	199,575	176,780	176,711	88.54%	99.96%	1
	7_1	690,000	463,825	699,847	302,228	65.16%	43.18%	2
	7_4	400,000	114,306	213,747	75,057	65.66%	35.11%	2
	8_1	150,000	-	49,669	-	#DIV/0!	0.00%	2
	8_3	500,000	648,939	384,776	376,906	58.08%	97.95%	1
	9_1	470,000	723,915	692,178	364,793	50.39%	52.70%	1
	9_5	710,000	560,120	561,174	555,890	99.24%	99.06%	1
	10_1	430,000	304,089	722,021	304,089	100.00%	42.12%	2
	10_2	400,000	132,951	360,868	132,951	100.00%	36.84%	2
	11_1	340,000	374,689	1,399,575	283,524	75.67%	20.26%	1
	12_1	420,000	532,816	369,450	369,438	69.34%	100.00%	1
	12_2	280,000	283,452	695,284	283,452	100.00%	40.77%	1
TS60	1_1	360,000	339,998	44,881	44,352	13.04%	98.82%	1
	3_1	470,000	392,172	336,323	333,943	85.15%	99.29%	1
	3_2	820,000	446,576	21,550	20,671	4.63%	95.92%	1
	4_1	700,000	581,420	210,894	210,167	36.15%	99.66%	1
	4_3	330,000	209,337	29,872	29,782	14.23%	99.70%	1
	5_1	730,000	373,939	37,158	37,110	9.92%	99.87%	1
	5_2	320,000	183,743	10,260	10,260	5.58%	100.00%	1
	6_1	140,000	153,092	39,224	37,992	24.82%	96.86%	1
	7_1	690,000	301,768	250,071	250,035	82.86%	99.99%	1
	7_4	400,000	197,723	23,300	22,160	11.21%	95.11%	1
	8_1	150,000	64,715	54,312	48,312	74.65%	88.95%	1
	8_3	500,000	205,375	8,692	8,582	4.18%	98.73%	1
	9_1	470,000	437,365	139,695	139,687	31.94%	99.99%	1
	9_5	710,000	325,367	17,427	17,364	5.34%	99.64%	1
	10_1	430,000	301,471	175,843	175,322	58.16%	99.70%	1
	10_2	400,000	213,902	54,589	53,839	25.17%	98.63%	1
	11_1	340,000	234,213	3,171	3,044	1.30%	95.99%	1
	12_1	420,000	286,148	110,454	110,401	38.58%	99.95%	1
	12_2	280,000	198,907	46,145	46,060	23.16%	99.82%	1
TS80	1_1	360,000	350,653	508,102	333,383	95.07%	65.61%	2
	3_1	470,000	414,493	437,222	344,434	83.10%	78.78%	1
	3_2	820,000	853,460	306,656	269,103	31.53%	87.75%	1
	4_1	700,000	802,759	627,347	413,241	51.48%	65.87%	1
	4_3	330,000	364,251	330,000	313,529	86.07%	95.01%	1
	5_1	730,000	872,606	157,142	157,078	18.00%	99.96%	1
	5_2	320,000	250,761	315779	161,888	64.56%	51.27%	1
	6_1	140,000	287,915	140,000	139,742	48.54%	99.82%	1
	7_1	690,000	314,796	368395	178,439	56.68%	48.44%	1
	7_4	400,000	177,911	184677	132,915	74.71%	71.97%	1
	8_1	150,000	55,915	150,000	44,610	79.78%	29.74%	2
	8_3	500,000	611,588	519,770	518,956	84.85%	99.84%	1
	9_1	470,000	463,558	562063	318,230	68.65%	56.62%	1
	9_5	710,000	899,758	710,289	542,034	60.24%	76.31%	1
	10_1	430,000	573,985	435,478	433,111	75.46%	99.46%	1
	10_2	400,000	499,810	459,451	459,451	91.93%	100.00%	1
	11_1	340,000	457,033	340,000	281,732	61.64%	82.86%	1
	12_1	420,000	515,460	483,122	478,695	92.87%	99.08%	1
	12_2	280,000	532,469	499,075	498,975	93.71%	99.98%	1
TS96	1_1	360,000	77,949	353,478	59,917	76.87%	16.95%	2
	3_1	470,000	371,529	342,764	342,764	92.26%	100.00%	1
	3_2	820,000	269,355	24,120	24,023	8.92%	99.60%	1
	4_1	700,000	406,149	210,767	196,413	48.36%	93.19%	1
	4_3	330,000	109,117	51,333	50,623	46.39%	98.62%	1
	5_1	730,000	485,313	70,814	68,383	14.09%	96.57%	1
	5_2	320,000	-	16,394	-	#DIV/0!	0.00%	2
	6_1	140,000	28,319	13,593	-	0.00%	0.00%	1
	7_1	690,000	568,077	235,949	235,890	41.52%	99.97%	1
	7_4	400,000	-	58,390	-	#DIV/0!	0.00%	2
	8_1	150,000	171,683	135,845	135,818	79.11%	99.98%	1
	8_3	500,000	101,615	16,596	16,418	16.16%	98.93%	1
	9_1	470,000	146,436	190,905	146,436	100.00%	76.71%	2
	9_5	710,000	493,988	20,694	19,688	3.99%	95.14%	1
	10_1	430,000	343,071	231,198	230,284	67.12%	99.60%	1
	10_2	400,000	309,613	83,121	83,065	26.83%	99.93%	1

Table S8

	11_1	340,000	47,255	-	-	0.00%	#DIV/0!	1
	12_1	420,000	168,568	132,291	127,697	75.75%	96.53%	1
	12_2	280,000	-	17,356	-	#DIV/0!	0.00%	2
TS112	1_1	360,000	619,134	180,352	176,560	28.52%	97.90%	1
	3_1	470,000	493,170	426,219	413,814	83.91%	97.09%	1
	3_2	820,000	888,116	102,130	101,325	11.41%	99.21%	1
	4_1	700,000	337,318	415,169	146,863	43.54%	35.37%	1
	4_3	330,000	427,271	214,070	214,005	50.09%	99.97%	1
	5_1	730,000	813,034	208,829	208,816	25.68%	99.99%	1
	5_2	320,000	393285	320000	319,035	81.12%	99.70%	1
	6_1	140,000	232,562	140,000	133,743	57.51%	95.53%	1
	7_1	690,000	764,442	731,966	731,955	95.75%	100.00%	1
	7_4	400,000	371,563	106,281	106,194	28.58%	99.92%	1
	8_1	150,000	222,748	370,562	204,113	91.63%	55.08%	1
	8_3	500,000	256,814	374,009	178,371	69.46%	47.69%	1
	9_1	470,000	507,751	470,000	319,477	62.92%	67.97%	1
	9_5	710,000	733,573	150,465	141,299	19.26%	93.91%	1
	10_1	430,000	525,564	471,771	471,737	89.76%	99.99%	1
	10_2	400,000	484,592	159,289	159,239	32.86%	99.97%	1
	11_1	340,000	282,245	392,828	244,557	86.65%	62.26%	1
	12_1	420,000	497,293	473,218	473,218	95.16%	100.00%	1
	12_2	280,000	324,773	279,823	275,603	84.86%	98.49%	1
TS117	1_1	360,000	-	69,413	-	#DIV/0!	0.00%	2
	3_1	470,000	310,253	353,730	276,760	89.20%	78.24%	1
	3_2	820,000	771,384	76,375	76,375	9.90%	100.00%	1
	4_1	700,000	618,471	261,016	243,031	39.30%	93.11%	1
	4_3	330,000	196,478	31,468	30,526	15.54%	97.01%	1
	5_1	730,000	-	134,290	-	#DIV/0!	0.00%	2
	5_2	320,000	93,221	23,385	22,830	24.49%	97.63%	1
	6_1	140,000	96,897	140,000	90,664	93.57%	64.76%	2
	7_1	690,000	185,065	313,450	153,820	83.12%	49.07%	2
	7_4	400,000	-	38,297	-	#DIV/0!	0.00%	2
	8_1	150,000	71,588	11,002	10,934	15.27%	99.38%	1
	8_3	500,000	186,688	17,604	17,449	9.35%	99.12%	1
	9_1	470,000	630,940	222,940	219,815	34.84%	98.60%	1
	9_5	710,000	590,443	14,203	14,161	2.40%	99.70%	1
	10_1	430,000	163,028	218,785	162,959	99.96%	74.48%	2
	10_2	400,000	65,073	48,861	41,448	63.69%	84.83%	1
	11_1	340,000	168,611	17,345	16,689	9.90%	96.22%	1
	12_1	420,000	169,513	114,107	114,063	67.29%	99.96%	1
	12_2	280,000	-	73,228	-	#DIV/0!	0.00%	2
TS118	1_1	360,000	461,371	39,925	38,248	8.29%	95.80%	1
	3_1	470,000	590,296	319,942	311,812	52.82%	97.46%	1
	3_2	820,000	320,944	29,944	29,910	9.32%	99.89%	1
	4_1	700,000	445,312	215,323	215,253	48.34%	99.97%	1
	4_3	330,000	270,338	18,163	18,089	6.69%	99.59%	1
	5_1	730,000	226,437	33,969	33,961	15.00%	99.98%	1
	5_2	320,000	234,624	17,697	17,611	7.51%	99.51%	1
	6_1	140,000	46,353	7,278	7,244	15.63%	99.53%	1
	7_1	690,000	449,220	223,287	223,118	49.67%	99.92%	1
	7_4	400,000	180,330	27,348	26,852	14.89%	98.19%	1
	8_1	150,000	91,958	36,718	36,664	39.87%	99.85%	1
	8_3	500,000	243,782	20,650	19,906	8.17%	96.40%	1
	9_1	470,000	199,420	146,602	145,576	73.00%	99.30%	1
	9_5	710,000	463,661	103,214	102,572	22.12%	99.38%	1
	10_1	430,000	292,773	174,310	174,284	59.53%	99.99%	1
	10_2	400,000	277,749	28,912	25,441	9.16%	87.99%	1
	11_1	340,000	98,597	15,999	14,588	14.80%	91.18%	1
	12_1	420,000	292,481	119,357	118,782	40.61%	99.52%	1
	12_2	280,000	198,180	9,101	8,754	4.42%	96.19%	1
TS156	1_1	360,000	317,830	18,700	15,875	4.99%	84.89%	1
	3_1	470,000	533,153	430,485	375,806	70.49%	87.30%	1
	3_2	820,000	1,311,185	174,731	171,897	13.11%	98.38%	1
	4_1	700,000	958,170	233,860	218,453	22.80%	93.41%	1
	4_3	330,000	234702	313669	83,305	35.49%	26.56%	2
	5_1	730,000	753,358	135,195	127,354	16.90%	94.20%	1
	5_2	320,000	319,284	34,618	34,547	10.82%	99.79%	1
	6_1	140,000	357,916	140,000	106,646	29.80%	76.18%	1
	7_1	690,000	533,129	343,638	274,760	51.54%	79.96%	1
	7_4	400,000	503,154	107,446	107,389	21.34%	99.95%	1
	8_1	150,000	143,308	79,653	72,676	50.71%	91.24%	1
	8_3	500,000	383,826	17,492	17,452	4.55%	99.77%	1
	9_1	470,000	389,208	213,221	213,140	54.76%	99.96%	1
	9_5	710,000	605,606	382,279	368,789	60.90%	96.47%	1
	10_1	430,000	507,149	174,540	174,039	34.32%	99.71%	1
	10_2	400,000	409,938	63,072	63,072	15.39%	100.00%	1
	11_1	340,000	359,827	52,568	52,568	14.61%	100.00%	1
	12_1	420,000	507,295	122,626	122,561	24.16%	99.95%	1
	12_2	280,000	267,410	223,362	165,819	62.01%	74.24%	1
TS166	1_1	360,000	829,392	689,364	682,509	82.29%	99.01%	1
	3_1	470,000	492,006	403,309	403,179	81.95%	99.97%	1
	3_2	820,000	993,392	103,435	103,281	10.40%	99.85%	1
	4_1	700,000	1,513,670	243,755	243,387	16.08%	99.85%	1
	4_3	330,000	238,704	256,803	106,234	44.50%	41.37%	2
	5_1	730,000	766,978	652,980	647,107	84.37%	99.10%	1

Table S8

	5_2	320,000	448,558	280,892	280,801	62.60%	99.97%	1
	6_1	140,000	212,763	140,000	137,988	64.86%	98.56%	1
	7_1	690,000	759,052	370,870	370,802	48.85%	99.98%	1
	7_4	400,000	427,864	379,558	378,432	88.45%	99.70%	1
	8_1	150,000	245,456	260,039	232,861	94.87%	89.55%	1
	8_3	500,000	385,113	50,851	50,813	13.19%	99.93%	1
	9_1	470,000	488,069	226,382	226,296	46.37%	99.96%	1
	9_5	710,000	791,431	517,778	510,583	64.51%	98.61%	1
	10_1	430,000	555,329	463,405	463,388	83.44%	100.00%	1
	10_2	400,000	418,009	317,253	279,800	66.94%	88.19%	1
	11_1	340,000	265,206	262,212	238,437	89.91%	90.93%	1
	12_1	420,000	540,750	507,952	507,916	93.93%	99.99%	1
	12_2	280,000	270,441	277,706	225,971	83.56%	81.37%	1
TS185	1_1	360,000	1,419,662	477,919	476,532	33.57%	99.71%	1
	3_1	470,000	435,590	425,749	398,880	91.57%	93.69%	1
	3_2	820,000	917,098	879,145	877,873	95.72%	99.86%	1
	4_1	700,000	746,409	666,228	661,190	88.58%	99.24%	1
	4_3	330,000	496,656	404,447	403,347	81.21%	99.73%	1
	5_1	730,000	869,391	802,799	802,754	92.34%	99.99%	1
	5_2	320,000	340,342	267,382	265,757	78.09%	99.39%	1
	6_1	140,000	287,840	140,000	139,081	48.32%	99.34%	1
	7_1	690,000	781,434	762,066	762,002	97.51%	99.99%	1
	7_4	400,000	475,820	400,000	398,597	83.77%	99.65%	1
	8_1	150,000	314,844	150,000	149,918	47.62%	99.95%	1
	8_3	500,000	597,857	153,219	153,160	25.62%	99.96%	1
	9_1	470,000	793,786	233,968	233,479	29.41%	99.79%	1
	9_2	210,000	243,809	210,000	209,962	86.12%	99.98%	1
	10_1	430,000	552,848	521,275	521,262	94.29%	100.00%	1
	10_2	400,000	298875	376203	268,531	89.85%	71.38%	2
	11_1	340,000	385,612	340,000	339,968	88.16%	99.99%	1
	12_1	420,000	490,866	420,000	419,103	85.38%	99.79%	1
	12_2	280,000	365,464	499,068	363,241	99.39%	72.78%	1
TS204	1_1	360,000	435,872	44,060	44,011	10.10%	99.89%	1
	3_1	470,000	383,355	366,343	365,189	95.26%	99.68%	1
	3_2	820,000	757,141	65,083	65,014	8.59%	99.89%	1
	4_1	700,000	581,725	228,920	228,140	39.22%	99.66%	1
	4_3	330,000	305,728	32,518	32,459	10.62%	99.82%	1
	5_1	730,000	516,312	102,038	101,914	19.74%	99.88%	1
	5_2	320,000	322,036	31,409	30,314	9.41%	96.51%	1
	6_1	140,000	147,571	55,522	55,452	37.58%	99.87%	1
	7_1	690,000	643,205	240,217	240,151	37.34%	99.97%	1
	7_4	400,000	357,960	60,508	60,198	16.82%	99.49%	1
	8_1	150,000	147,339	49,494	49,422	33.54%	99.85%	1
	8_3	500,000	328,944	39,573	39,523	12.02%	99.87%	1
	9_1	470,000	540,906	215,564	215,508	39.84%	99.97%	1
	9_5	710,000	727,865	82,158	73,935	10.16%	89.99%	1
	10_1	430,000	438,172	243,990	242,880	55.43%	99.55%	1
	10_2	400,000	302,280	65,009	64,596	21.37%	99.36%	1
	11_1	340,000	194,379	70,414	69,378	35.69%	98.53%	1
	12_1	420,000	428,390	122,191	122,174	28.52%	99.99%	1
	12_2	280,000	301,921	16,367	16,289	5.40%	99.52%	1
TS222	1_1	360,000	498,178	360,000	2,210	0.44%	0.61%	2
	3_1	470,000	506,184	470,000	469,957	92.84%	99.99%	1
	3_2	820,000	944,490	761,234	759,878	80.45%	99.82%	1
	4_1	700,000	544,711	620,478	239,895	44.04%	38.66%	1
	4_3	330,000	406,638	326,950	292,833	72.01%	89.57%	1
	5_1	730,000	784,347	318,620	312,388	39.83%	98.04%	1
	5_2	320,000	216,639	940,150	271,220	125.19%	28.85%	2
	6_1	140,000	141,003	140,000	137,086	97.22%	97.92%	1
	7_1	690,000	738,479	706,772	706,737	95.70%	100.00%	1
	7_4	400,000	338,085	259,250	221,562	65.53%	85.46%	1
	8_1	150,000	354,469	197,621	197,598	55.74%	99.99%	1
	8_3	500,000	572,788	553,745	553,708	96.67%	99.99%	1
	9_1	470,000	515,796	282,989	242,742	47.06%	85.78%	1
	9_5	710,000	711,342	655,678	638,074	89.70%	97.32%	1
	10_1	430,000	388,904	230,299	230,230	59.20%	99.97%	1
	10_2	400,000	460,058	337,333	302,759	65.81%	89.75%	1
	11_1	340,000	170,393	600,588	70,353	41.29%	11.71%	2
	12_1	420,000	500,944	242,894	233,037	46.52%	95.94%	1
	12_2	280,000	228,878	420,336	182,110	79.57%	43.32%	2
TS238	1_1	360,000	171,013	167,833	123,852	72.42%	73.79%	1
	3_1	470,000	373,565	599,173	346,015	92.63%	57.75%	2
	3_2	820,000	893,351	151,090	81,191	9.09%	53.74%	1
	4_1	700,000	1,042,631	650,276	650,260	62.37%	100.00%	1
	4_3	330,000	314,073	376,648	291,955	92.96%	77.51%	2
	5_1	730,000	933,698	259,141	252,279	27.02%	97.35%	1
	5_2	320,000	437,408	320,000	319,942	73.14%	99.98%	1
	6_1	140,000	163,108	144,109	144,064	88.32%	99.97%	1
	7_1	690,000	898,398	528,384	524,869	58.42%	99.33%	1
	7_4	400,000	559,519	343,964	338,255	60.45%	98.34%	1
	8_1	150,000	231,645	44,464	44,389	19.16%	99.83%	1
	8_3	500,000	543,626	402,844	338,438	62.26%	84.01%	1
	9_1	470,000	732234	465,462	184,252	25.16%	39.58%	2
	9_5	710,000	578,524	31,431	31,360	5.42%	99.77%	1
	10_1	430,000	597,278	490,096	489,508	81.96%	99.88%	1

Table S8

	10_2	400,000	472,601	401,289	400,152	84.67%	99.72%	1
	11_1	340,000	571,534	340,000	333,403	58.33%	98.06%	1
	12_1	420,000	510,569	474,285	474,214	92.88%	99.99%	1
	12_2	280,000	402,237	31,829	31,751	7.89%	99.75%	1
TS265	1_1	360,000	67,113	52,441	52,406	78.09%	99.93%	1
	3_1	470,000	379,561	312,037	280,269	73.84%	89.82%	1
	3_2	820,000	744,690	64,233	63,821	8.57%	99.36%	1
	4_1	700,000	739,349	236,577	225,230	30.46%	95.20%	1
	4_3	330,000	196,430	36,208	36,152	18.40%	99.85%	1
	5_1	730,000	210,345	51,489	22,877	10.88%	44.43%	1
	5_2	320,000	222,130	26,572	26,556	11.96%	99.94%	1
	6_1	140,000	166,535	106,970	72,266	43.39%	67.56%	1
	7_1	690,000	415,527	238,142	190,168	45.77%	79.85%	1
	7_4	400,000	-	49,951	-	#DIV/0!	0.00%	2
	8_1	150,000	-	30,571	-	#DIV/0!	0.00%	2
	8_3	500,000	387,001	21,367	21,260	5.49%	99.50%	1
	9_1	470,000	-	312,872	-	#DIV/0!	0.00%	2
	9_5	710,000	585,610	-	-	0.00%	#DIV/0!	1
	10_1	430,000	285,613	248,176	193,051	67.59%	77.79%	1
	10_2	400,000	127,438	52,995	48,666	38.19%	91.83%	1
	11_1	340,000	166,740	10,878	10,808	6.48%	99.36%	1
	12_1	420,000	309,521	121,110	120,607	38.97%	99.58%	1
	12_2	280,000	118,838	17,493	17,447	14.68%	99.74%	1
TS280	1_1	360,000	213,532	150,748	145,217	68.01%	96.33%	1
	3_1	470,000	485,398	388,359	339,023	69.84%	87.30%	1
	3_2	820,000	2,011,773	177,235	170,928	8.50%	96.44%	1
	4_1	700,000	789,844	486,969	241,395	30.56%	49.57%	1
	4_3	330,000	430,625	114,042	114,003	26.47%	99.97%	1
	5_1	730,000	780,217	159,774	159,727	20.47%	99.97%	1
	5_2	320,000	473,227	299,960	294,183	62.17%	98.07%	1
	6_1	140,000	208,569	144,103	144,054	69.07%	99.97%	1
	7_1	690,000	774,306	835,623	762,661	98.50%	91.27%	2
	7_4	400,000	369,654	212,395	212,328	57.44%	99.97%	1
	8_1	150,000	256,407	149,879	149,733	58.40%	99.90%	1
	8_3	500,000	390,284	143,158	108,083	27.69%	75.50%	1
	9_1	470,000	482,338	470,000	289,773	60.08%	61.65%	2
	9_5	710,000	632,764	645,558	361,030	57.06%	55.93%	1
	10_1	430,000	536,640	389,791	388,868	72.46%	99.76%	1
	10_2	400,000	156,551	400,000	123,091	78.63%	30.77%	2
	11_1	340,000	415,437	340,000	339,930	81.82%	99.98%	1
	12_1	420,000	504,535	456,210	456,189	90.42%	100.00%	1
	12_2	280,000	280,612	215,191	161,054	57.39%	74.84%	1
TS281	1_1	360,000	241,701	173,611	173,602	71.83%	99.99%	1
	3_1	470,000	496,357	390,089	390,058	78.58%	99.99%	1
	3_2	820,000	850,776	781,845	781,548	91.86%	99.96%	1
	4_1	700,000	814,786	697,525	626,844	76.93%	89.87%	1
	4_3	330,000	481,071	414,394	326,030	67.77%	78.68%	1
	5_1	730,000	814,166	720,959	720,892	88.54%	99.99%	1
	5_2	320,000	385,963	346,471	346,395	89.75%	99.98%	1
	6_1	140,000	289,038	140,000	139,961	48.42%	99.97%	1
	7_1	690,000	745,932	242,500	219,882	29.48%	90.67%	1
	7_4	400,000	412,067	400,000	395,141	95.89%	98.79%	1
	8_1	150,000	311,019	140,896	140,854	45.29%	99.97%	1
	8_3	500,000	573,927	534,446	534,051	93.05%	99.93%	1
	9_1	470,000	928,939	1,049,074	169,263	18.22%	16.13%	1
	9_5	710,000	850,224	566,643	563,904	66.32%	99.52%	1
	10_1	430,000	597,115	498,389	498,340	83.46%	99.99%	1
	10_2	400,000	538,942	429,126	429,108	79.62%	100.00%	1
	11_1	340,000	394,705	674,965	294,991	74.74%	43.70%	1
	12_1	420,000	436,012	564,161	427,664	98.09%	75.81%	1
	12_2	280,000	529,949	422,229	324,355	61.20%	76.82%	1
TS331	1_1	360,000	154,253	574,734	136,855	88.72%	23.81%	2
	3_1	470,000	481,287	470,000	469,950	97.64%	99.99%	1
	3_2	820,000	991,782	534,229	529,944	53.43%	99.20%	1
	4_1	700,000	729,560	710,714	710,667	97.41%	99.99%	1
	4_3	330,000	344,416	127,665	127,566	37.04%	99.92%	1
	5_1	730,000	266,116	554,359	159,149	59.80%	28.71%	2
	5_2	320,000	410,435	379,605	360,434	87.82%	94.95%	1
	6_1	140,000	200,690	140,000	133,756	66.65%	95.54%	1
	7_1	690,000	866,189	762,399	762,353	88.01%	99.99%	1
	7_4	400,000	480,562	400,000	332,857	69.26%	83.21%	1
	8_1	150,000	66,527	74,099	66,467	99.91%	89.70%	2
	8_3	500,000	575,043	574,212	563,849	98.05%	98.20%	1
	9_1	470,000	549,890	470,000	446,290	81.16%	94.96%	1
	9_5	710,000	737,623	725,433	717,560	97.28%	98.91%	1
	10_1	430,000	540,207	389,722	387,229	71.68%	99.36%	1
	10_2	400,000	465,710	433,223	430,812	92.51%	99.44%	1
	11_1	340,000	376,986	340,000	339,944	90.17%	99.98%	1
	12_1	420,000	508,398	442,633	431,478	84.87%	97.48%	1
	12_2	280,000	296,352	504,759	279,514	94.32%	55.38%	2
TS413	1_1	360,000	121,613	25,270	25,248	20.76%	99.91%	1
	3_1	470,000	259,146	479,252	154,821	59.74%	32.30%	2
	3_2	820,000	1,054,369	857,037	639,786	60.68%	74.65%	1
	4_1	700,000	805,589	505,727	352,658	43.78%	69.73%	1
	4_3	330,000	427,028	185,223	182,750	42.80%	98.66%	1

Table S8

	5_1	730,000	459,466	378,370	339,867	73.97%	89.82%	1
	5_2	320,000	192,789	309,614	169,583	87.96%	54.77%	2
	6_1	140,000	144,275	161,035	88,311	61.21%	54.84%	1
	7_1	690,000	545,639	205,497	198,860	36.45%	96.77%	1
	7_4	400,000	156,200	400,000	148,140	94.84%	37.04%	2
	8_1	150,000	99,294	56,836	51,218	51.58%	90.12%	1
	8_3	500,000	968,629	503,859	296,787	30.64%	58.90%	1
	9_1	470,000	1,786,460	733,624	570,812	31.95%	77.81%	1
	9_5	710,000	893,101	824,476	824,624	92.33%	100.02%	1
	10_1	430,000	461,880	260,874	254,879	55.18%	97.70%	1
	10_2	400,000	264,663	79,463	79,387	30.00%	99.90%	1
	11_1	340,000	211,498	70,929	65,162	30.81%	91.87%	1
	12_1	420,000	442,423	919,717	270,015	61.03%	29.36%	1
	12_2	280,000	411,486	98,909	94,751	23.03%	95.80%	1
TS421	1_1	360,000	389,053	114,227	104,676	26.91%	91.64%	1
	3_1	470,000	513,226	352,151	351,043	68.40%	99.69%	1
	3_2	820,000	1,304,831	350,200	350,141	26.83%	99.98%	1
	4_1	700,000	690,356	877,717	513,936	74.45%	58.55%	2
	4_3	330,000	241,845	40,064	15,757	6.52%	39.33%	1
	5_1	730,000	429,516	92,383	78,633	18.31%	85.12%	1
	5_2	320,000	281,728	234,985	215,517	76.50%	91.72%	1
	6_1	140,000	99,020	140,000	64,009	64.64%	45.72%	2
	7_1	690,000	766,084	743,935	743,900	97.10%	100.00%	1
	7_4	400,000	454,187	400,000	204,275	44.98%	51.07%	2
	8_1	150,000	130,192	150,000	117,631	90.35%	78.42%	2
	8_3	500,000	621,691	38,135	38,098	6.13%	99.90%	1
	9_1	470,000	487,234	602,170	279,966	57.46%	46.49%	1
	9_5	710,000	755,584	710,000	308,642	40.85%	43.47%	1
	10_1	430,000	525,538	462,487	462,438	87.99%	99.99%	1
	10_2	400,000	271,134	400,000	139,963	51.62%	34.99%	1
	11_1	340,000	385,657	198,630	179,966	46.66%	90.60%	1
	12_1	420,000	448,178	208,140	208,102	46.43%	99.98%	1
	12_2	280,000	531,475	496,172	496,119	93.35%	99.99%	1
TS439	1_1	360,000	434,251	38,857	34,463	7.94%	88.69%	1
	3_1	470,000	377,926	345,363	340,396	90.07%	98.56%	1
	3_2	820,000	984,593	228,948	204,554	20.78%	89.35%	1
	4_1	700,000	765,292	250,990	249,978	32.66%	99.60%	1
	4_3	330,000	388,868	151,805	108,914	28.01%	71.75%	1
	5_1	730,000	804,502	700,000	98,592	12.26%	14.08%	1
	5_2	320,000	362,016	21,475	21,427	5.92%	99.78%	1
	6_1	140,000	183,999	140,000	139,095	75.60%	99.35%	1
	7_1	690,000	626,548	332,523	332,477	53.06%	99.99%	1
	7_4	400,000	462,128	400,000	366,390	79.28%	91.60%	1
	8_1	150,000	100,908	150,000	70,414	69.78%	46.94%	2
	8_3	500,000	590,109	290,026	271,040	45.93%	93.45%	1
	9_1	470,000	445,415	140,888	140,838	31.62%	99.96%	1
	9_5	710,000	816,707	462,491	462,449	56.62%	99.99%	1
	10_1	430,000	435,250	272,302	239,637	55.06%	88.00%	1
	10_2	400,000	296,362	372,417	274,359	92.58%	73.67%	1
	11_1	340,000	381,840	82,343	81,836	21.43%	99.38%	1
	12_1	420,000	617,003	172,405	172,340	27.93%	99.96%	1
	12_2	280,000	321,817	276,831	275,747	85.68%	99.61%	1
TS545	1_1	360,000	331,764	30,579	29,260	8.82%	95.69%	1
	3_1	470,000	308,020	288,875	288,827	93.77%	99.98%	1
	3_2	820,000	420,124	38,548	37,725	8.98%	97.86%	1
	4_1	700,000	366,989	201,641	200,636	54.67%	99.50%	1
	4_3	330,000	146,890	33,926	32,881	22.38%	96.92%	1
	5_1	730,000	162,085	54,299	54,231	33.46%	99.87%	1
	5_2	320,000	223,452	9,909	9,765	4.37%	98.55%	1
	6_1	140,000	129,905	39,637	39,145	30.13%	98.76%	1
	7_1	690,000	365,863	232,609	232,552	63.56%	99.98%	1
	7_4	400,000	267,059	23,259	23,212	8.69%	99.80%	1
	8_1	150,000	71,839	42,101	42,047	58.53%	99.87%	1
	8_3	500,000	-	21,413	-	#DIV/0!	0.00%	2
	9_1	470,000	613,221	154,056	137,124	22.36%	89.01%	1
	9_5	710,000	-	9,206	-	#DIV/0!	0.00%	2
	10_1	430,000	110,287	206,923	110,205	99.93%	53.26%	2
	10_2	400,000	221,840	32,456	32,105	14.47%	98.92%	1
	11_1	340,000	222,467	6,038	5,859	2.63%	97.04%	1
	12_1	420,000	219,135	101,280	101,248	46.20%	99.97%	1
	12_2	280,000	166,542	33,391	33,369	20.04%	99.93%	1
TS623	1_1	360,000	542,645	100,863	92,399	17.03%	91.61%	1
	3_1	470,000	259,372	697,659	261,094	100.66%	37.42%	2
	3_2	820,000	1,000,335	926,223	926,142	92.58%	99.99%	1
	4_1	700,000	564,315	565,607	451,908	80.08%	79.90%	1
	4_3	330,000	246,431	162,076	161,966	65.72%	99.93%	1
	5_1	730,000	233,169	554,355	212,214	91.01%	38.28%	2
	5_2	320,000	428,600	320,000	318,668	74.35%	99.58%	1
	6_1	140,000	198,293	140,000	139,293	70.25%	99.50%	1
	7_1	690,000	766,189	751,364	748,064	97.63%	99.56%	1
	7_4	400,000	368,493	335,745	335,701	91.10%	99.99%	1
	8_1	150,000	181,618	150,000	149,924	82.55%	99.95%	1
	8_3	500,000	577,759	26,793	25,565	4.42%	95.42%	1
	9_1	470,000	398,038	470,000	139,978	35.17%	29.78%	1
	9_5	710,000	761,024	269,931	220,441	28.97%	81.67%	1

Table S8

	10_1	430,000	305,680	236,198	216,017	70.67%	91.46%	1
	10_2	400,000	487,963	400,000	399,438	81.86%	99.86%	1
	11_1	340,000	370,558	340,000	339,950	91.74%	99.99%	1
	12_1	420,000	588,529	556,179	554,367	94.20%	99.67%	1
	12_2	280,000	287,064	225,110	117,722	41.01%	52.30%	1
TS629	1_1	360,000	492,962	761,210	489,680	99.33%	64.33%	2
	3_1	470,000	481,933	470,000	469,947	97.51%	99.99%	1
	3_2	820,000	852,329	868,546	822,307	96.48%	94.68%	1
	4_1	700,000	718,295	699,903	699,039	97.32%	99.88%	1
	4_3	330,000	441,712	219,473	215,857	48.87%	98.35%	1
	5_1	730,000	837,382	361,448	359,580	42.94%	99.48%	1
	5_2	320,000	421,362	320,000	319,443	75.81%	99.83%	1
	6_1	140,000	126,954	133,988	105,689	83.25%	78.88%	1
	7_1	690,000	773,972	759,329	757,283	97.84%	99.73%	1
	7_4	400,000	387,357	336,223	336,155	86.78%	99.98%	1
	8_1	150,000	251,720	140,896	140,620	55.86%	99.80%	1
	8_3	500,000	549,790	25,154	25,105	4.57%	99.81%	1
	9_1	470,000	438,579	1,055,277	139,148	31.73%	13.19%	1
	9_5	710,000	791,698	710,000	709,959	89.68%	99.99%	1
	10_1	430,000	547,653	505,591	505,228	92.25%	99.93%	1
	10_2	400,000	488,979	457,654	457,611	93.59%	99.99%	1
	11_1	340,000	413,855	340,000	338,657	81.83%	99.61%	1
	12_1	420,000	783,897	550,036	525,894	67.09%	95.61%	1
	12_2	280,000	304,116	520,443	304,061	99.98%	58.42%	2
TS692	1_1	360,000	350,383	242,220	236,872	67.60%	97.79%	1
	3_1	470,000	473,036	445,271	445,208	94.12%	99.99%	1
	3_2	820,000	1,002,878	626,199	586,903	58.52%	93.72%	1
	4_1	700,000	957,584	705,789	705,729	73.70%	99.99%	1
	4_3	330,000	616,714	411,638	411,575	66.74%	99.98%	1
	5_1	730,000	809,534	284,358	284,254	35.11%	99.96%	1
	5_2	320,000	463,360	320,000	307,151	66.29%	95.98%	1
	6_1	140,000	239,883	140,000	139,325	58.08%	99.52%	1
	7_1	690,000	801,449	750,432	745,090	92.97%	99.29%	1
	7_4	400,000	372,913	355,948	355,274	95.27%	99.81%	1
	8_1	150,000	322,635	150,000	149,935	46.47%	99.96%	1
	8_3	500,000	609,178	174,191	171,130	28.09%	98.24%	1
	9_1	470,000	457,391	365,329	331,143	72.40%	90.64%	1
	9_5	710,000	837,907	124,420	124,418	14.85%	100.00%	1
	10_1	430,000	533,759	505,103	500,115	93.70%	99.01%	1
	10_2	400,000	221,744	400,000	209,986	94.70%	52.50%	2
	11_1	340,000	261,156	635,213	78,920	30.22%	12.42%	2
	12_1	420,000	537,187	457,362	452,732	84.28%	98.99%	1
	12_2	280,000	308,372	303,614	256,569	83.20%	84.50%	1

Table S8: The tomato subtelomeres included in the population-level analysis.

Subtelomere: the IDs of the subtelomeres tested in the experiments; X_Y shows the format of ID; X represents the ID of chromosome and Y (1 or 2) shows the ID of each subtelomere for each chromosome
Ref length: the length of the corresponding tandem repeat in reference genome
TRFill length: the length of TRFill assembly
Original length: the length of original (hifiasm+3ddna) assembly
Common: the total length of the common regions of TRFill assembly and original assembly
Common/TRFill: the total length of the common regions / the length of TRFill assembly
Common/Original: the total length of the common regions / the length of original assembly
Partition: 1 represents the subtelomeres with TRFill assembly length > original assembly length; 2 represents the other subtelomeres
